# New Transferrin Receptor‐Targeting Conjugate Effectively Delivers DNA to Mouse Brain

**DOI:** 10.1002/anie.202500247

**Published:** 2025-04-10

**Authors:** Min‐sun Song, Adrian H. Bustos, Lise Bastue, Justas Mikutavicius, Piotr Swiderski, Kelly J. Clemens, Nagy Habib, John Rossi, Kira Astakhova

**Affiliations:** ^1^ Department of Molecular and Cellular Biology City of Hope National Medical Center City of Hope, 1500 East Duarte Road Duarte 91010 USA; ^2^ Department of Chemistry Organic and Inorganic Chemistry Technical University of Denmark Kemitorvet, Building 207 Lyngby 2800 Kgs Denmark; ^3^ School of Psychology Faculty of Science University New South Wales Kensington New South Wales 2052 Australia; ^4^ Department of Surgery & Cancer Imperial College London Hammersmith Campus Du Cane Road London W12 0NN UK

**Keywords:** Anti‐miR, Aptamer, BBB, Nucleic acid delivery, Transferrin receptor

## Abstract

Delivery across the blood–brain barrier (BBB) is one of the most challenging tasks for modern biopharmaceutics. Many attempts have been taken, with only low delivery efficacies achieved so far. We report a new transferrin receptor‐targeting (TfR) RNA aptamer conjugated to DSPE lipid that leads to an unprecedented effective uptake in the brain, with brain‐to‐serum ratios up to 6.5 in mice. This result is superior to recently published values of < 1 for antibody conjugates and nanovesicles, pointing to a successful combined effect of the increased lipophilicity and TfR targeting with the new RNA aptamer that our conjugate provides. Using fluorescence whole body imaging, polymerase‐chain reaction (PCR) and fluorescence in situ hybridization, we confirm that the new conjugate delivers high amounts of DNA oligonucleotide to brains of Balb/cJ mice, and it is effective in human cells. There is no acute toxicity as verified with histopathological assessment of mice organs. The combination of properties demonstrated by our new conjugate makes it a highly potent delivery tool that can be applied in therapy of brain diseases incl. glioblastoma, neurogenerative diseases, and a broad range of brain infections.

## Graphical abstract

We present a novel RNA aptamer targeting the transferrin receptor (TfR), conjugated with DSPE lipid, which achieves remarkably efficient brain uptake, reaching brain‐to‐serum ratios as high as 6.5. Through fluorescence imaging and polymerase‐chain reaction analyses, we verify that this new conjugate successfully delivers a substantial amount of DNA oligonucleotide to the brains of Balb/cJ mice and demonstrates effectiveness in a human blood–brain barrier cell line model.

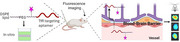

The blood–brain barrier (BBB) is formed by tight junctions of epithelial cells, with limited and strictly controlled transport of molecules and cells across this interface.^[^ ^1^ ^]^ The brain‐to‐blood ratio is a critical parameter in evaluating the ability of drugs, including antibody‐small molecule conjugates and nucleic acids, to cross the BBB and reach the brain after systemic administration.

Conjugation strategies can enhance brain delivery. Reported brain‐to‐blood ratios for such conjugates vary depending on the nature of the antibody, the small molecule, and the targeting mechanism (e.g., receptor‐mediated transcytosis), but typically are in the range of 0.01 to 0.1.^[^ ^2^, ^3^ ^]^ This is because antibodies are large, hydrophilic molecules that do not easily cross the BBB. Conjugation to small molecules and targeting receptor‐mediated transcytosis pathways effectively increases these ratios to 0.2 to 0.5.^[^ ^4^, ^5^ ^]^

Nucleic acids, such as small interfering RNA (siRNA), antisense oligonucleotides (ASOs), and mRNA, face significant challenges in crossing the BBB.^[^ ^6^ ^]^ Like antibodies, these molecules are typically large and hydrophilic, which limits their brain penetration and leads to brain‐to‐blood ratios below 0.01. Lipid Nanoparticles (LNPs) and other delivery systems designed to enhance BBB penetration can increase the brain‐to‐serum ratio of nucleic acids to the range of 0.01 to 0.05.^[^ ^7^, ^8^ ^]^ While this is an improvement, it still represents limited brain exposure relative to blood levels.

Aptamer technology is a promising way to increase BBB delivery for a wide range of compounds. Aptamers are short, single‐stranded oligonucleotides (DNA or RNA) or peptides that can fold into specific three‐dimensional shapes, allowing them to bind to a variety of target molecules with high affinity and specificity. They are often compared to antibodies in their ability to bind targets, but aptamers have an advantage of smaller size and easier synthesis compared to the antibodies. Aptamers can be designed to target and cross the BBB, either by binding to transport proteins naturally expressed on the BBB or by binding to receptors that mediate transcytosis (a process that shuttles molecules across BBB).^[^ ^9^, ^10^ ^]^

Transferrin is a glycoprotein that binds and transports iron throughout the body. The transferrin receptor (TfR) is widely expressed on BBB.^[^ ^11^ ^]^ In this work, we report a new effective reagent for delivery of oligonucleotides across BBB that combines a new TfR‐targeting RNA aptamer and lipophilicity‐enhancing lipid. We used the TAT peptide as a control due to its well‐documented ability to penetrate cell membranes, which has made it a standard comparison in delivery studies of nucleic acids.^[^ ^12^, ^13^ ^]^ The TAT peptide is derived from the trans‐activating transcriptional activator (TAT) protein of HIV‐1, known for its cell‐penetrating properties.^[^ ^14^ ^]^

Our method is illustrated in Scheme 1. TfR‐targeting RNA aptamer was generated with systematic evolution of ligands by exponential enrichment (SELEX).^[^ ^15^, ^16^ ^]^ The aptamer library selection utilized 2′‐fluoro modified ribonucleoside triphosphates (NTPs), specifically 2′‐fluoro‐2′‐deoxyuridine‐5′‐triphosphate (2′F dUTP), 2′‐fluoro‐2′‐deoxycytidine‐5′‐triphosphate (2′F dCTP), guanosine triphosphate (GTP), and adenosine triphosphate (ATP), in the in vitro transcription RNA synthesis process. Upon successful selection, the aptamer sequence was resynthesized using standard solid‐phase RNA synthesis (see Supporting Information).^[^ ^17^ ^]^

**Scheme 1:**
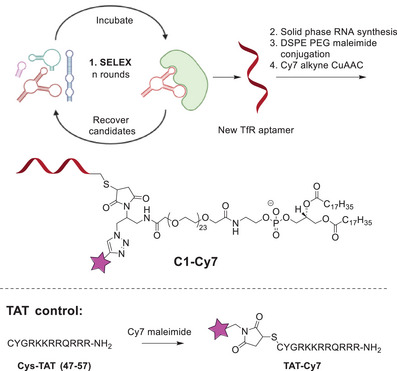
Main steps of this study: 1) SELEX provides new TfR aptamer. Each incubation round is performed under more stringent conditions, reduced TfR concentration details provided in Figure S1. Binding properties of TfR aptamers and hTfR protein determined with Biacore software are given in Table S1; *K*_D_ (M) 3.17E‐11 for full length aptamer, 2.20E‐11 for truncated aptamer; 2) Solid‐phase RNA synthesis with required modifications; sequence: *5′‐ThioMC6‐ iSpC3 iSpC3 i2FU i2FU i2FU rA i2FU i2FU i2FC rA i2FC rA i2FU i2FU i2FU i2FU i2FU rGrArA i2FU i2FU rG rA ‐3′*, where *ThioMC6 = *thiol modifier C6 linker, iSpC3 = spacer C3, i2F = 2′F‐2′‐deoxy; 3) Bioconjugation to DSPE‐PEG with copper‐catalyzed alkyne‐azide cycloaddition (CuAAC); 4) Labelling with Cy7. TAT peptide control is labelled with Cy7 maleimide providing TAT‐Cy7.

Binding properties of TfR aptamers and target protein determined with Biacore software are given in Table S1; *K*_D_ (M) 3.17E‐11 for full length aptamer, 2.20E‐11 for truncated aptamer. A *K*_D_ in the low nanomolar to picomolar range is lower than that of other reported monoclonal antibodies,^[^ ^18^ ^]^ and can result from our SELEX conditions. While high‐affinity binding is generally advantageous for target specificity and stability, extremely low *K*_D_ values can reduce the efficiency of receptor‐mediated transcytosis, as tight binding may impede receptor recycling and cargo release.^[^ ^19^ ^]^ In antibody‐based delivery systems, an intermediate *K*_D_ is often preferred to balance target engagement with efficient intracellular trafficking. Future studies could explore modifications to the aptamer structure to fine‐tune its affinity for optimal transcytosis efficiency.

The aptamer was further modified with 5′‐terminal sulfide group that allowed for orthogonal bioconjugation to maleimide‐DSPE‐PEG reagent. At the final step, the conjugate (C1) was labelled with cyanine 7 (Cy7) using click chemistry to generate conjugate C1‐Cy7.^[^ ^20^ ^]^ As a control, we used TAT peptide that was labelled with Cy7 as well. The products were purified and characterized with high‐performance liquid chromatography (HPLC) and mass spectrometry (Supporting Information). Further, the half‐lifetime of the product RNA conjugate in mouse serum was measured to be 3.9 h, which was improved from 1.0 h for unconjugated RNA aptamer (Figure S4). According to previous papers this is due to increased hydrophobicity of the conjugate versus unmodified RNA.^[^ ^21^, ^22^ ^]^

We tested the new conjugate and TAT peptide control in a cell model of the human BBB.^[^ ^23^ ^]^ Human neurons were grown with a layer of hcmec/d3 epithelial cells above them. With this assay, we confirmed high passage efficacy for both C1‐Cy7 and TAT‐Cy7 through the hcmec/d3 (Supporting Information). This relates to previous results by us and others showing that TAT and other peptides enhance uptake of nanoparticles and biomolecular analogues in cellular BBB models by more than two‐fold compared to unmodified controls.^[^ ^24^, ^25^, ^26^ ^]^

Cellular tests alone cannot provide information on organ targeting and biodistribution. We therefore proceeded testing the conjugates in vivo. Male and female Balb/cJ mice received i.v. injection of C1‐Cy7 or TAT‐Cy7 (see Supporting Information for details on in vivo experiments). The homology of human and mouse transferrin receptors is >90% analyzed with Clustal Omega^[^ ^27^ ^]^; this makes aptamer suitable for targeting both species. In the preliminary study 20 and 10 mg kg^−1^ of C1‐C7 were evaluated; with the former dose giving sufficient signal in subsequent live mice fluorescence imaging, and lower dose being weaker detected in the whole‐body imaging experiments (Figures S10 and S11). We therefore proceeded with the dose 20 mg kg^−1^. Biodistribution of compounds in mice was evaluated longitudinally with fluorescence imaging.^[^ ^28^ ^]^ As can be seen in Figures 1,S8 and S9, C1‐Cy7 effectively accumulated in mice brain, whereas TAT remained in liver of the mice. Accumulation of C1‐Cy7 in the brain was further confirmed after dissection of the mice (Figure 1c). Pre‐injection data points and group treated with phosphate‐buffered saline (PBS) showed no signal in similar microscopy setting (Figure S12).

**Figure 1:**
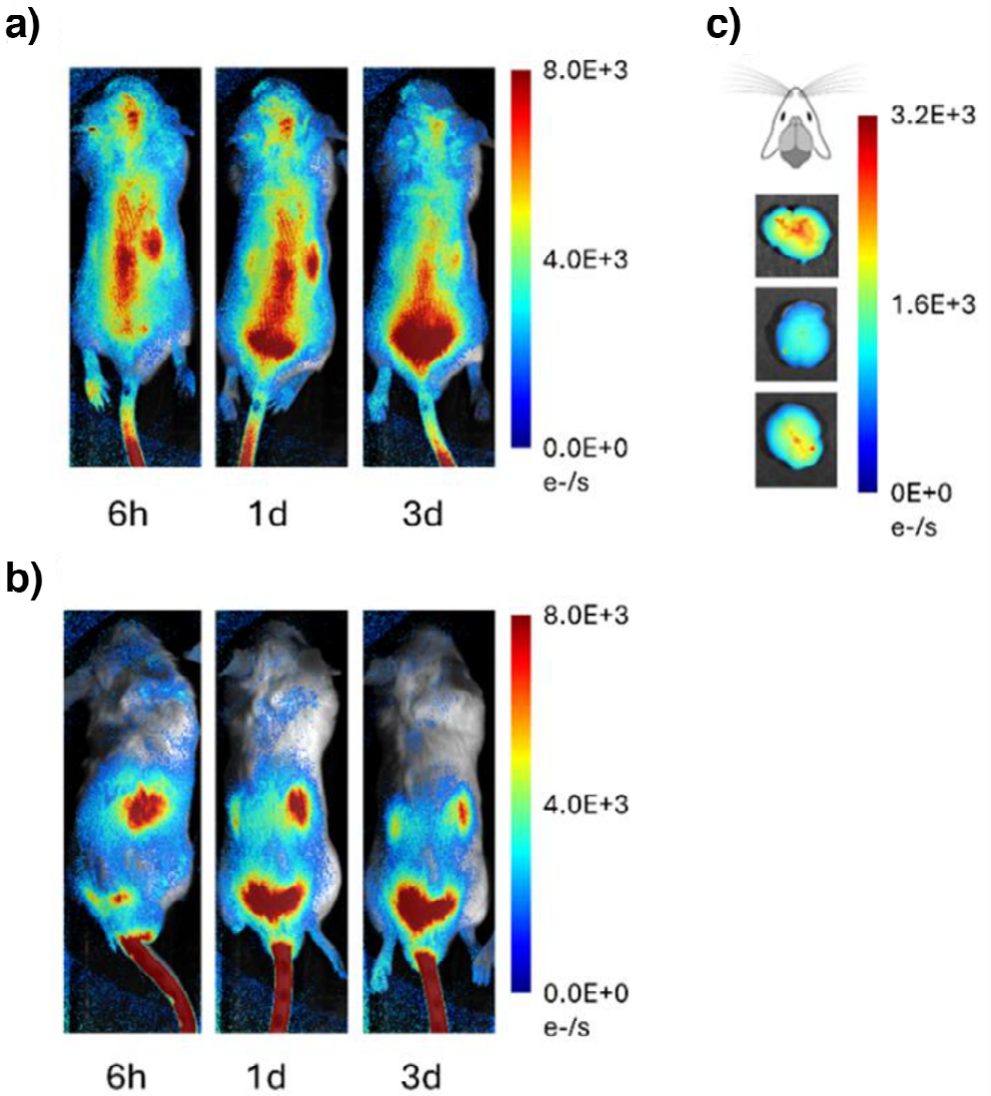
Whole‐body fluorescence images for C1‐Cy7 a) and TAT‐Cy7 b) administered i.v. into male Balb/cJ mice. c) Images of fluorescence in harvested brains of male mice 1 day post‐i.v. injection of C1‐Cy7, 20 mg kg^−1^. Three mice were used per group for whole body imaging experiments.

We analyzed biodistribution in male Balb/cJ (Figures 2a and S8–S12). The brain, liver, kidney, and lungs take up significant amounts of C1‐Cy7, with comparatively less in the spleen. Harvesting followed by brain dissection and fluorescence imaging also revealed that the areas with highest uptake of C1‐Cy7 were the frontal lobes, and less the cerebellum and brainstem (Figure 2b).

**Figure 2:**
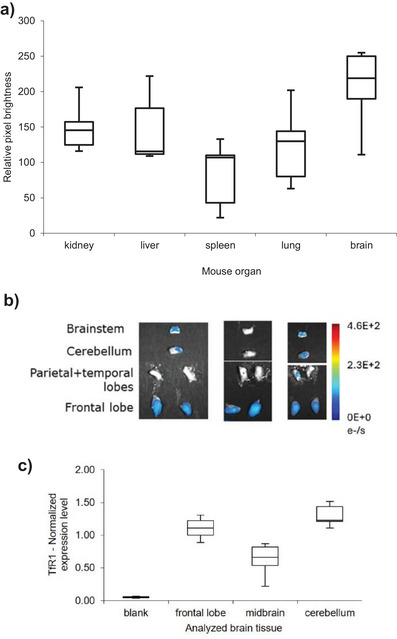
Key results of in vivo study in male Balb/cJ mice treated with C1–Cy7, 20 mg kg^−1^: a) Boxplot for mice 2 days post‐administration of C1‐Cy7 (*n* = 3). Pixel brightness analyzed for harvested organs (Figure S9), 5 random points per mouse organ assessed with ImageJ; *p* = 1.3e‐10 (**, 99% significance; ANOVA). b) Fluorescence images of harvested mice brains, 2 days post‐administration of C1‐Cy7. Images shown for biological triplicates. c) Boxplot for the normalized expression levels of TfR1 in 7‐week‐old healthy untreated Balb/cJ mice brains, determined with RT‐qPCR. Analyses done in intra‐plate duplicates for five female and five male mice.

We examined the correlation between TfR1 expression and biodistribution results in the brains of untreated Balb/cJ mice. Tissue samples were collected from distinct brain regions, processed, and analyzed using RT‐qPCR (see Supporting Information for details). TfR1 expression was indeed highest in the cerebellum and frontal lobes, showing an average two‐fold increase in these areas compared to the midbrain (Figure 2c). This pattern potentially explains the biodistribution data observed in Figure 2b.

In the next step we investigated the localization of the test compound C1‐Cy7 in the brain tissue following systemic administration in male mice. Two mice were treated with 20 mg kg^−1^ of C1‐Cy7, and brain tissue samples were subsequently analyzed using fluorescence in situ hybridization (FISH) with specific markers for blood vessels (*CD31*) and neurons (*NeuN*) (Figure  3 and Supporting Information). The markers were labeled with Alexa Fluor 488 (green) for *CD31* and Alexa Fluor 555 (red) for *NeuN* (Figure 3a and Supporting Information), while the test compound was visualized in the Cy7 channel (near‐infrared emission, ∼775 nm; white signal in Figure 3). The results demonstrated that the Cy7 signal of C1‐Cy7 was located both near the neuronal marker (red) and the blood vessels (dark green). This finding confirms that C1‐Cy7 does not only remain in blood vessels but also is taken up by neurons. Location in neurons was further confirmed by images taken at increased magnification (Figure 3c). Control images were taken for mice treated with TAT Cy7 and PBS and showed no signal in the similar microscopy settings (Figure S17).

**Figure 3:**
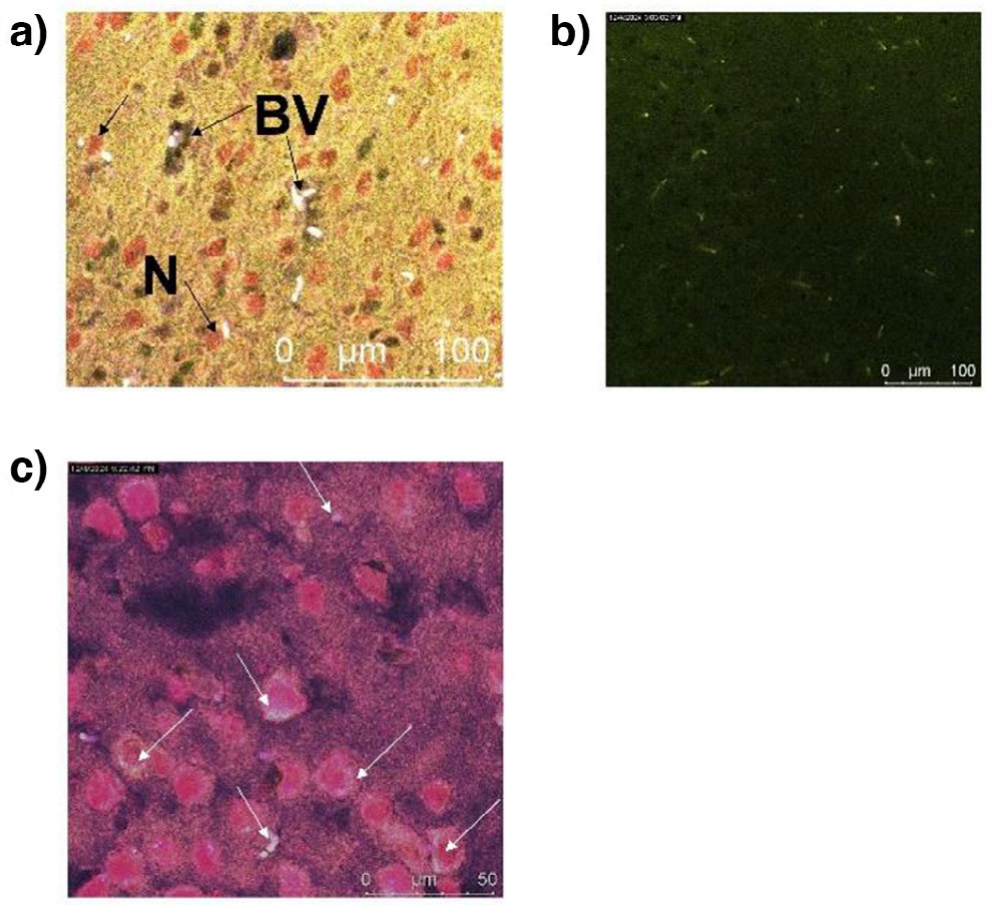
Fluorescence in situ hybridization results for brain tissue of male mice treated with C1 Cy7, 20 mg kg^−1^. Cy7 (conjugate) is seen in white; CD31 (blood vessels, BV) and NeuN (neurons, N) are visualized in dark green and red, respectively. a) Complete staining of the sample imaged in a multiplex setting. b) Unstained tissue showing exclusively Cy7 signal (light dots). c) 40x Magnification image shows location of C1 Cy7 (white) near neurons (red).

To further investigate the brain delivery capability of the new conjugate, we linked it to a DNA strand targeting human miR‐125b1 (Figure 4a). miR‐125b1 is a microRNA—a type of small, non‐coding RNA involved in gene regulation of inflammatory responses and oxidative stress, thereby affecting neuronal growth and apoptosis. Specifically, miR‐125b1 targets Sphingosine Kinase 1 (SphK1), a critical cell‐signaling enzyme that converts sphingosine to sphingosine‐1‐phosphate (S1P), a bioactive lipid.^[^ ^29^ ^]^ This enzyme plays a role in regulating cell growth, survival, migration, and immune response. High levels of miR‐125b are observed in Alzheimer's disease, where it has been associated with cognitive impairment and contributes to abnormal cell proliferation, differentiation, and apoptosis in cancer and inflammatory diseases.^[^ ^30^ ^]^ Targeting miR‐125b1 with an anti‐miR may offer a therapeutic strategy to address these debilitating effects. To stabilize DNA molecule of anti‐miR in vivo, we incorporated locked nucleic acids (LNA).^[^ ^31^ ^]^ At this stage, Cy7 label was not applied, and instead anti‐miR was “clicked” to the aptamer‐DSPE‐PEG (C1) (Figure 4a). The product conjugate C2 was tested in human neuroblastoma cells SH‐SY5Y where it proved to reduce cell viability in a dose‐dependent manner. 34% Reduction of cell viability at 450 nM concentration of C2 correlated with high expression level of miR‐125b1 in SH‐SY5Y cells (Supporting Information). Viability of healthy epithelial control cells BEAS‐2B which expressed lower level of miR‐125b1, was less affected, with only 13% reduced viability at 450 nM concentration of C2 (Supporting Information).

**Figure 4:**
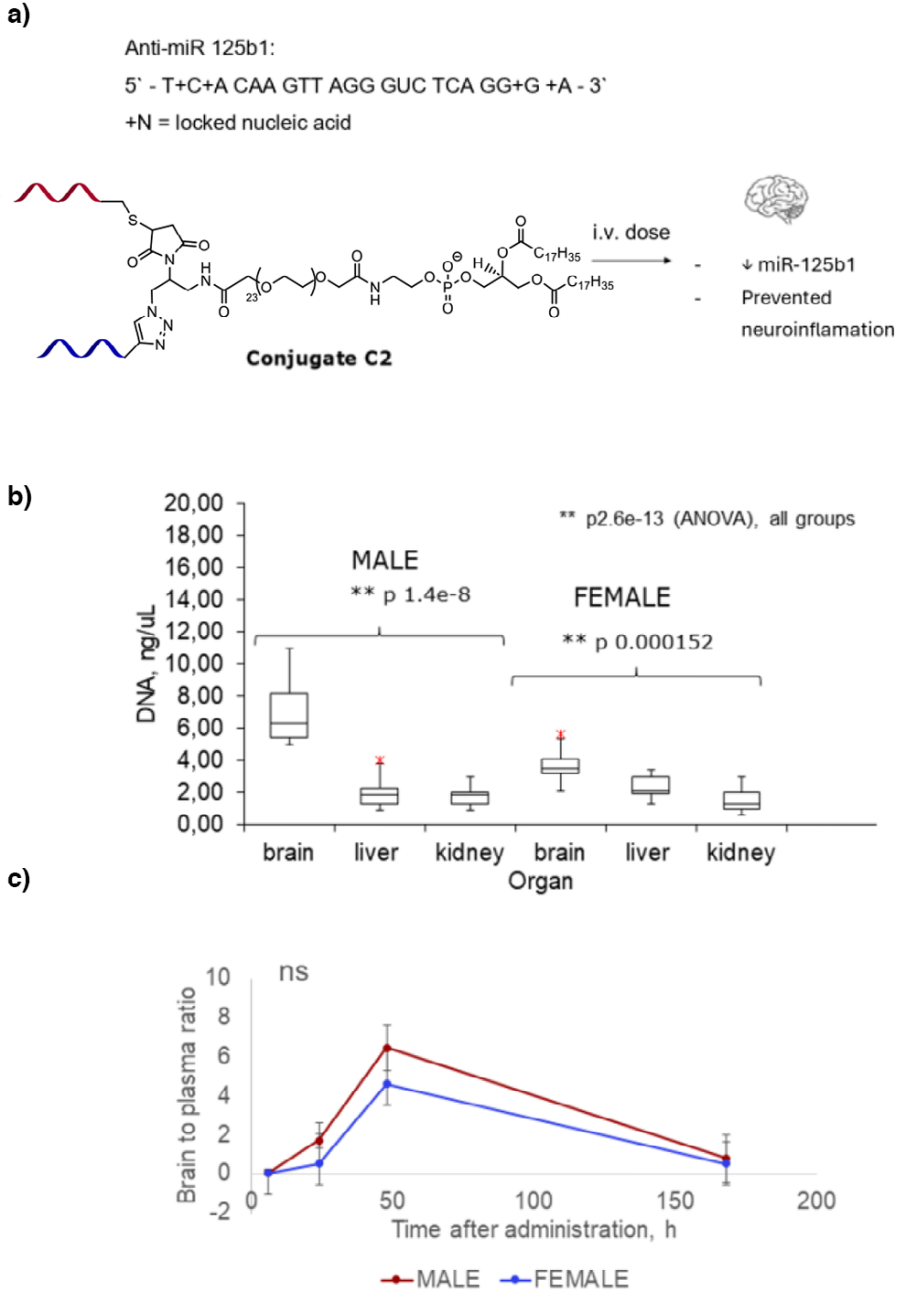
C2 effectively delivers anti‐miR to mouse brain. a) Sequence of anti‐miR 125b1; b) qPCR levels of anti‐miR 125b1 in brain, liver and kidney of mice (*n* = 3); C) Brain to serum ratio for anti‐miR 125b1. ** 99% significance level, ns = not significant (ANOVA). c) *p* = 0.44 (ANOVA). Error bars show coefficient of variance (CV%) for biological triplicates.

For brain targeting study, C2 was injected i.v. in male and female Balb/cJ mice at 5 mg.kg^−1^. The dose was deliberately reduced compared to C1‐Cy7, to reduce accumulation in other organs such as kidney and liver. After 2 days mice were harvested, their organs were processed, and DNA levels were analyzed with qPCR (Supporting Information). Remarkably, levels of DNA in the mice brain were higher than in kidney and liver (Figure 4b). Brain‐to‐serum ratios were also remarkably high, reaching 4.6–6.5 within 2 days of post‐injection. To the best of our knowledge, there is no precedent in the literature on achieving this high level of DNA delivery through the BBB.^[^ ^2^, ^3^, ^4^, ^5^, ^6^, ^7^, ^8^ ^]^ For example, for the commercially available monoclonal anti‐transferrin antibody Ox26‐76, the reported brain‐to‐plasma ratio range is only 0.01–0.1.^[^ ^18^ ^]^ This confirms that the combination of hydrophilicity and BBB‐targeting aptamer at small conjugate size, i.e., without nanoparticle formulation, is a potent design for brain targeting. One‐week post‐injection anti‐miR was cleared from both brain and serum (Figure 4c).

Safety of treatment is an important consideration. We analyzed histopathology of both male and female mice 1 week after receiving C1‐Cy7, TAT‐Cy7, and PBS (negative control). Nucleic acid molecules have reported toxicity, affecting mainly liver and kidney.^[^ ^32^, ^33^, ^34^ ^]^ Therefore, in addition to the targeted organ, the brain, we analyzed kidney, liver, spleen, and lung pathology. These organs have different regeneration properties.^[^ ^29^ ^]^ Liver and spleen cells have high propensity of mitosis and recover faster after chemical or mechanical injury. Kidney and brain cells, on the contrary do not divide actively through mitosis, making them a target for acute and prolonged pathology. Lung cells have some ability to divide through mitosis and therefore are moderately subjected to persistent pathology.^[^ ^29^ ^]^ We found that indeed, kidney is the organ that is most negatively affected with the TAT treatment (Figures S18 and S19). Moreover, lungs showed high pathology as well, especially after receiving TAT (Figure S15). Spleen and liver were not affected (Figures S20–S23). In the brain, we observed reduced cell counts and small number of infiltrates; only in the case of mice receiving TAT (Figures S27–S32).

In conclusion, we synthesized and tested a new TfR‐targeting conjugate that demonstrates both high efficacy and low acute toxicity in Balb/cJ mice. The conjugate does not exclusively target the brain. It also affects the kidney, liver, and spleen. Nevertheless, it has an unprecedentedly high brain‐to‐serum ratio that opens the path for further developments in this direction. The combination of high brain targeting ability upon systemic administration and no acute toxicity is unique and makes our conjugate a potent new delivery tool for the brain.

## Supporting Information

The authors have cited additional references within the Supporting Information.^[^ ^35^, ^36^, ^37^, ^38^, ^39^, ^40^, ^41^, ^42^, ^43^, ^44^, ^45^, ^46^, ^47^ ^]^

## Conflict of Interests

The authors declare no conflict of interest.

## Supporting information



Supporting Information

## Data Availability

The data that support the findings of this study are available in the Supporting Information of this article.
