# Supplementary material for: New Transferrin Receptor‐Targeting Conjugate Effectively Delivers DNA to Mouse Brain
Source: Angew Chem Int Ed Engl. 2025 Apr 10;64(24):e202500247. doi: 10.1002/anie.202500247 (PMC12144868; doi:10.1002/anie.202500247)
Supplement: Supplementary file 1 — Supporting Information [file ANIE-64-e202500247-s001.pdf]

Supporting information for the paper:

New Transferrin Receptor-Targeting Conjugate Effectively Delivers DNA to Mouse Brain

Min-sun Song,[a],γ Adrian H. Bustos,[b],γ Lise Bastue,[b] Justas Mikutavicius,[b] Piotr Swiderski,[a] Kelly J. Clemens,[c] Nagy Habib,[d] John Rossi,[a]* and Kira Astakhova[b]*

---

[a] Dr. M-s. Song,γ Prof. P. Swiderski, Prof. J. Rossi*
Department of Molecular and Cellular Biology
City of Hope National Medical Center
City of Hope, 1500 East Duarte Road, Duarte, CA 91010
E-mail: jrossi@coh.org* shared corresponding authors

[b] A. Bustos,γ L. Bastue, J. Mikutavicius, Assoc. Prof. K. Astakhova*
Department of Chemistry, Organic and Inorganic Chemistry
Technical University of Denmark
Kemitorvet, Building 207, 2800 Kgs. Lyngby, Denmark
Email: kiraas@kemi.dtu.dk* shared corresponding authors
γ shared first authorship

[c] Prof. K. Clemens
School of Psychology
Faculty of Science
University New South Wales
Kensington, New South Wales 2052, Australia

[d] Prof. N. Habib
Department of Surgery & Cancer
Imperial College London
Hammersmith Campus, Du Cane Road, London W12 0NN, UK

Supporting information for this article is given via a link at the end of the document.

Contents

1. General 3

2. RNA Systematic Evolution of Ligands by EXponential enrichment (SELEX) 3

3. Solid-phase RNA synthesis 6

4. Binding Affinity of TfR Aptamer 6

5. Bioconjugations and product characterization 8

6. Tests in human cells 17

7. Studies in vivo 21

8. qPCR measurements 27

9. Histopathology 33

10. Statistical analyses 72

11. References 72

1. General

Cysteine- (Cys-)TAT(47-57) was purchased from Anaspec/Bionordika Denmark (cat no AS-61212), at HPLC purity > 95%, and used as received.

Cy7 reagents were purchased from Lumiprobe: alkyne (cat no A50B0); maleimide (cat no 15080). 1,2-Distearoyl-sn-glycero-3-phosphoethanolamine-N-[amino(polyethylene glycol) (DSPE PEG) 1k *N*-Hydroxysuccinimide (NHS) ester (cat no PLS-9916) was purchased from Creative PEG works and used as received.

Linker compound for bioconjugations was kindly provided by MRC Labs, UK, and used as received.

Anti-microRNA (anti-miR) molecule was designed with Python script using miR base for target miR 125b1 sequence. Conservation was checked for human vs mice miR. The resulting sequence of the applied anti-miR reagent was as follows:

/5Hexynyl//iSp9/T+C+ACAAGTTAGGGUCTCAGG+G+A

Anti-miR reagent was purchased from IDT and used as received (HPLC purity > 93%).

Cell lines were purchased from ATCC and Sigma.

All other reagents and kits were purchased from commercial vendors (Sigma, ThermoFisher), and used as received.

Fluorescence read out was done using microplate reader Magellan Tecan.

2. RNA Systematic Evolution of Ligands by EXponential enrichment (SELEX)

In vitro selection was enacted as previously described[35]. The RNA library was produced via in vitro transcription of randomized synthetic DNA oligonucleotides using nucleoside triphosphates (NTPs) and T7 RNA polymerase. The sequence of the RNA library is 5'-GGGAGAGCGGAAGCGUGCUGGGCC-N40-CAUAACCCAGAGGUCGAUGGAUCCCCCC -3', where N40 represents 40 nucleotides with equimolar incorporation of A, G, C, and U at each position. Human transferrin receptor (hTfR) was purchased from Sino Biological (11020-H07H; Beijing, P.R. China). The extracellular domain of hTfR (NP_003225.2) (Cys 89-Phe 760) was expressed with a Histidine^6 (His^6)-tag at the N terminus in human cells (HEK293). 2'F-RNA aptamers which bound with hTfR protein were nominated from 40-nt randomized sequences constructed by in vitro transcription of synthetic with DNA templates, NTPs (2'F dUTP [2'-Fluoro-2'-deoxyuridine-5'-triphosphate], 2'F dCTP [2'-Fluoro-2'-deoxycytidine-5'-triphosphate], GTP, ATP; Epicenter Biotechnologies, Madison, WI, USA) and T7 RNA polymerase. To remove RNAs that bound nonspecifically to agarose beads, 1.44 µM of the RNA library was pre-incubated with 20 µL of Ni-NTA agarose beads in 100 µL of binding buffer (30 mM Tris-HCl [pH 7.5]; 150 mM NaCl; 5 mM $MgCl_2$; 2 mM dithiothreitol; 100 µg/mL yeast tRNA; 1% bovine serum albumin BSA) for 30 min at room temperature with shaking, precipitated by centrifugation, and discarded. The precleared

supernatant was collected and reacted with 300 nM His^6-tagged hTfR for 30 min at room temperature. RNAs bound to hTfR were recovered, amplified by reverse transcription polymerase chain reaction (RT-PCR) and in vitro transcription, and used in subsequent selection rounds. In subsequent rounds, the hTfR concentration was reduced by 2-fold every three rounds for more stringent conditions. After nine rounds of SELEX, the resulting cDNA was amplified. The amplified DNA was cloned, and individual clones were identified by DNA sequencing.

Aptamer structures were predicted using Mfold, employing a salt correction algorithm and temperature correction for 25°C, or predicted using NUPACK online software. Based on structural analysis using computational prediction, the TfR aptamer was truncated into the smallest functional unit expected to maintain binding to hTfR, generating a truncated TfR aptamer with 22 nucleotides.

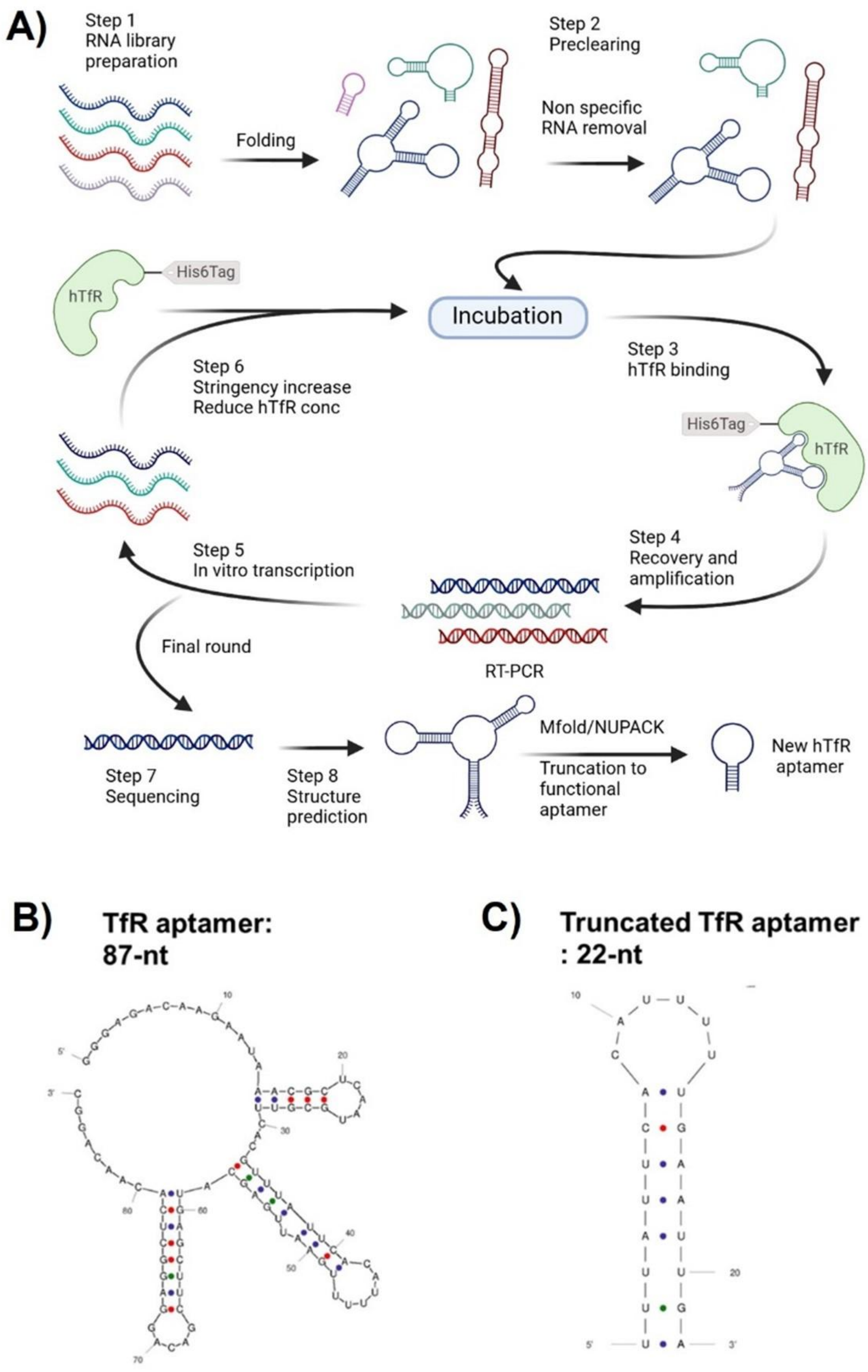

Figure S1. A) Workflow to obtain new TfR aptamer: 1) library preparation with automated solid-phase RNA synthesis; 2) precleaning; 3) incubation with human transferrin receptor (hTfR); 4) recovery of binders and amplification with RT-PCR; 5) in vitro transcription; 6) stringency increase procedure; 7) after n rounds of steps 3-6, sequencing of lead RNA; 8) structure analyses and optimization; B) Secondary structure of full length hTfR aptamer obtained with SELEX; C) Secondary structure of truncated hTfR aptamer. RNA structures were predicted using the mFold RNA structure program.

3. Solid-phase RNA synthesis

The resulting truncated RNA aptamer sequence was as follows:

tTfR aptamer (22-nt) :

5' i2FU i2FU i2FU rA i2FU i2FU i2FC rA i2FC rA i2FU i2FU i2FU i2FU i2FU rGrArA i2FU i2FU rG rA-3'

With solid-phase synthesis, **tTfR aptamer** and **RNA sequence NH726** were synthesized. **NH726** contained additional thiol group on the 5' end for upcoming bioconjugation:

5'- 5ThioMC6-D iSpC3 iSpC3 i2FU i2FU i2FU rA i2FU i2FU i2FC rA i2FC rA i2FU i2FU i2FU i2FU i2FU rGrArA i2FU i2FU rG rA

where 5ThioMC6 = thiol modifier C6 linker, iSpC3 = spacer C3, i2F = 2'F-2'-deoxy.

4. Binding Affinity of TfR Aptamer

The binding affinity of the TfR aptamer was measured using a surface plasmon resonance (SPR)-based biosensor assay, as previously described.[35] The Biacore T100 (GE Healthcare, Sweden) was employed to monitor real-time, label-free interactions between the TfR aptamer and hTfR protein. A biotinylated aptamer was coupled to a streptavidin-coated Biacore chip (SensorChip SA, BR-1003-98; General Electric Company) by injection in binding buffer (30 mM Tris-HCl [pH 7.5], 150 mM NaCl, 5 mM $MgCl_2$) at a concentration of 25 µg/mL and a flow rate of 10 µL/min. The RNA was refolded by heating to 65°C, followed by cooling to 37°C before immobilization. To measure binding kinetics, five different concentrations of purified hTfR protein were injected at a flow rate of 10 µL/min. Following binding, the surface was regenerated by injecting a dissociation buffer of 50 mM NaOH at a flow rate of 15 µL/min for 20 seconds. Data from the control surface was removed. BIAevaluation software (GE Healthcare) was utilized for analysis. The binding data were fitted to a one-to-one ratio binding model with mass transfer to calculate kinetic parameters.

Kinetic parameters for the interaction between the TfR aptamer and hTfR protein were calculated using a 1:1 binding model with mass transfer. The association rate constant (K_a) was determined by fitting the association phase of the binding curve, representing the rate at which the aptamer binds to the hTfR protein. The dissociation rate constant (K_d) was determined by fitting the dissociation phase of the binding curve, representing the rate at which the aptamer dissociates from the hTfR protein. The equilibrium dissociation constant (KD) was calculated as the ratio of K_d to K_a (KD = K_d / K_a), indicating the affinity of the aptamer for the hTfR protein. Lower KD values correspond to higher binding affinity.

Table S1. Binding properties of TfR aptamers and tTfR Protein determined with Biacore software.

	Size	K_a ($M^{-1}s^{-1}$)	K_d (S^{-1})	K_D (M)
TfR aptamer	87-nt	6.78E+03	1.66E-06	3.17E-11
tTfR aptamer	22-nt	6.30E+06	1.38E-04	2.20E-11

5. Bioconjugations and product characterization

Maleimide-azido-DSPE (**7**) reagent was prepared by reaction of DSPE-PEG 1k NHS ester (6) with 1-amino-3-azidopropan-2-ol (**5**) (Scheme S1).

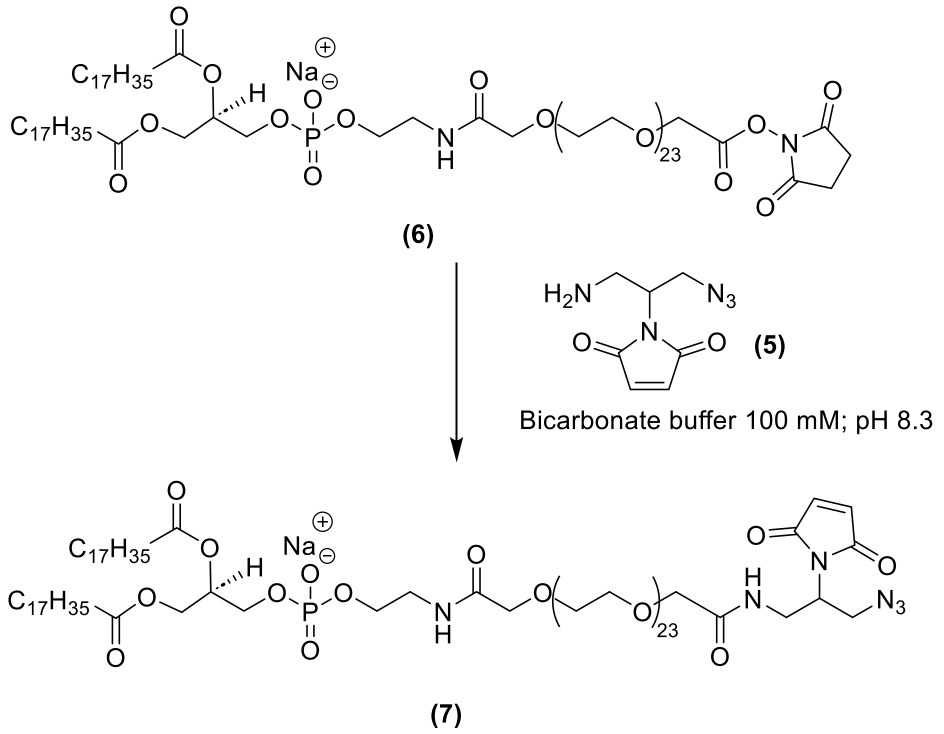

Scheme S1. Synthesis of azido-DSPE-PEG 1k maleimide reagent (**7**).

General procedure for maleimide conjugations

Sulfhydryl-containing compound (50 nmol; RNA aptamer-SH or Cys-TAT) in 110 mM sterile-filtered phosphate-buffered saline (PBS) pH 7.0 was incubated with 20 mM tris(2-carboxyethyl)phosphine (TCEP) (final volume 200 μL) for 30 min in argon atmosphere, followed by adding maleimide reagent (20 nmol; azido-DSPE-PEG conjugate (**7**) or Cy7-maleimide) to afford C1 or TAT-Cy7 conjugates, respectively (Scheme S2).

pH of TCEP stock was adjusted to 7.0 prior to use. Incubation was kept at room temperature for 3 hours followed by purification with MWKO 10k Amicon device centrifugation and washing 2 times 1x PBS.

General procedure for CuAAC conjugations

Azide-containing product C1 (50 nmol) was dissolved in 0.1M TEAA, pH 7.2, and mixed subsequently with alkyne-reagent (200 nmol; Cy7 alkyne or anti-miR alkyne), Cu-Tris((1-benzyl-4-triazolyl)methyl)amine (TBTA) (10 nmol) and freshly ascorbic acid (5 nmol), in a total volume of 200 μL. The reaction was degassed with argon and kept at room temperature for 6h, followed by purification with MWKO 10k Amicon device centrifugation and washing 2 times 1x PBS to afford conjugates C1-Cy7 and C2 (Scheme S2).

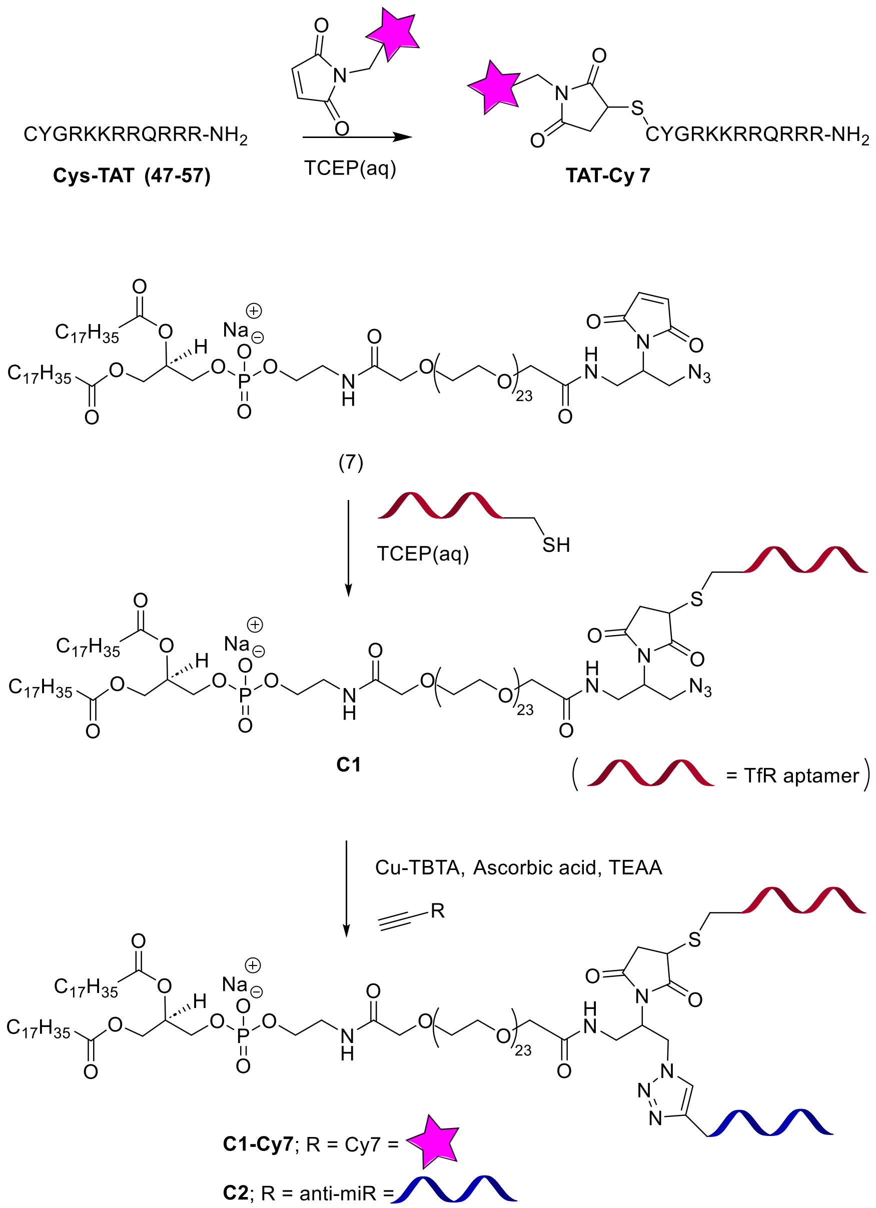

Scheme S2. Cy7-labelling of Cys-TAT peptide and conjugation of TfR aptamer and anti-miR. TCEP = tris(2-carboxyethyl)phosphine; TBTA = Tris((1-benzyl-4-triazolyl)methyl)amine; TEAA = triethylammonium acetate

Conjugate characterization

For purification and characterization of conjugates, HPLC was used with the following conditions:

Stationary phase: Poly(styrene-divinylbenzene)

Column ultra-high-performance liquid chromatography (UHPLC)+ focused Dionex Ultimate 3000 equipped with a DNAPac reversed phase (RP), Analytical, 4 µm, 3.0 × 100 mm

Buffers: Buffer A (triethylammonium acetate (TEAA) in MQ water, 0.05 M, pH 7.4) and buffer B (MeCN 75% in TEEA 0.0125 M)

Gradient: 5-60% of buffer B in a total run time of 30 min. Flow rate was 0.5 mL/min and UV for collection was 260 nm. Applied injection volume for analytical method was 12 µL

Matrix-Assisted Laser Desorption/Ionization - Time of Flight Mass Spectrometry (MALDI-TOF MS): was obtained on Bruker Autoflex Speed with TOF mass analyzer using a matrix of 3-hydroxypicolinic acid and MQ water. To determine masses using MALDI-TOF MS, 0.5 µL matrix and 0.5 µL sample were spotted.

Unconjugated RNA aptamer with a sulfide modification (**NH726)** and product C1 (0.5 µM each) were incubated with mouse serum (90 v %) in 1x HBSS at 37 °C, at final sample volume 0.5 mL. Aliquots were taken at different time points and directly resolved by HPLC. Each sample was analyzed in duplicate. Resulting areas under HPLC curve were plotted, and half-lifetimes were calculated using the following equation:

$N\ (t) = N\ (0) \times 0.5^{(t/T)}$,

Where:

N (*t*) – Remaining quantity of a substance after time *t* has elapsed;

N (0) - Initial quantity of this substance; and

T – half-life.

$$N(t) = N(0) \times 0.5^{(t/T)},$$

Where:

- $N(t)$ – Remaining quantity of a substance after time t has elapsed;
- $N(0)$ – Initial quantity of this substance; and
- T – half-life.

Characterization of conjugation products

Table S2. Characterization of conjugation products used in this study.

Conjugate	Ret time HPLC, min	Purity HPLC	Calcd MS Na^+ (phosphate)	MALDI TOF MS found
C1	6.25	95%	9599	9658 (+2K^+)
C1-Cy7	7.00	89%	10221	10348 (+3K^+)
C2	16.24	88%	16883	17002 (+3K^+)

A) C1

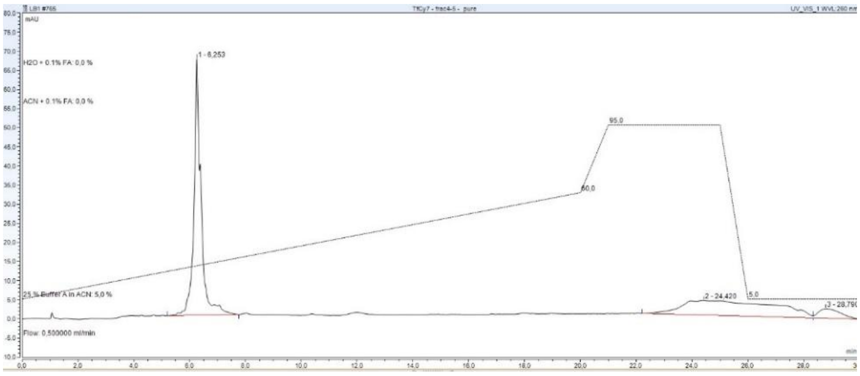

B) C1-Cy7

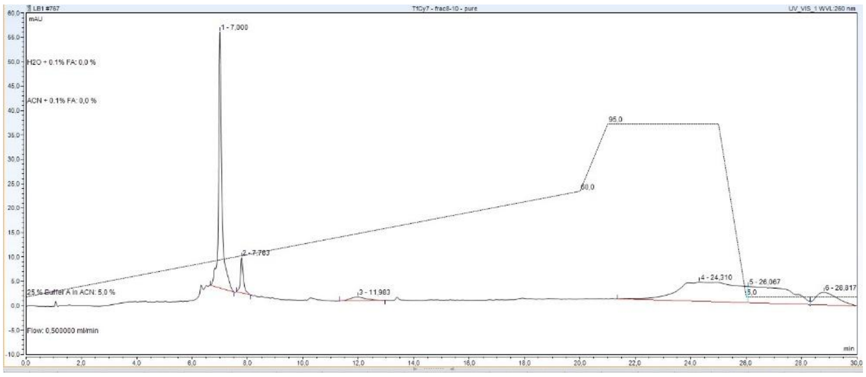

C) C2

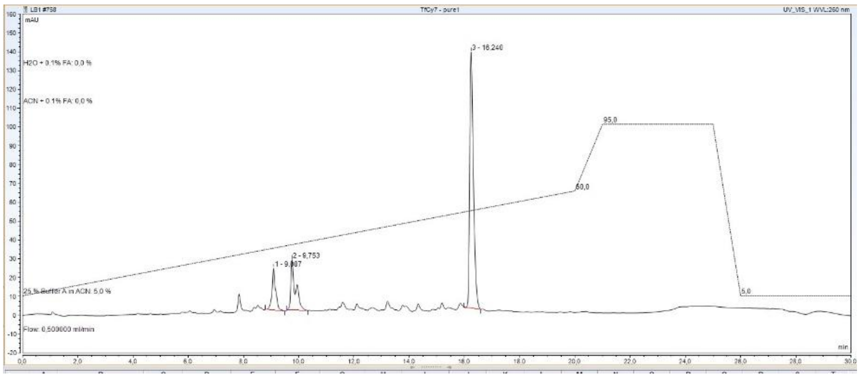

Figure S2. HPLC spectra of product conjugates used in this study: C1 (A), C1-Cy7 (B) and C2 (C).

A) C1

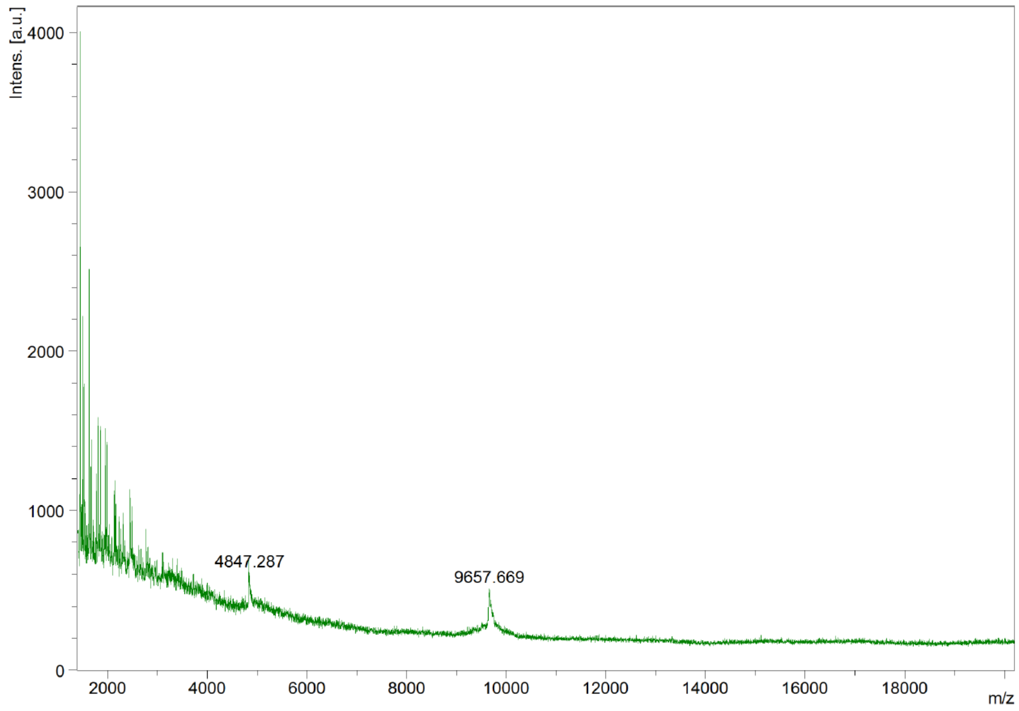
Intens. [a.u.]
4000
3000
2000
1000
0
4847.287
9657.669
2000
4000
6000
8000
10000
12000
14000
16000
18000
m/z

B) C1-Cy7

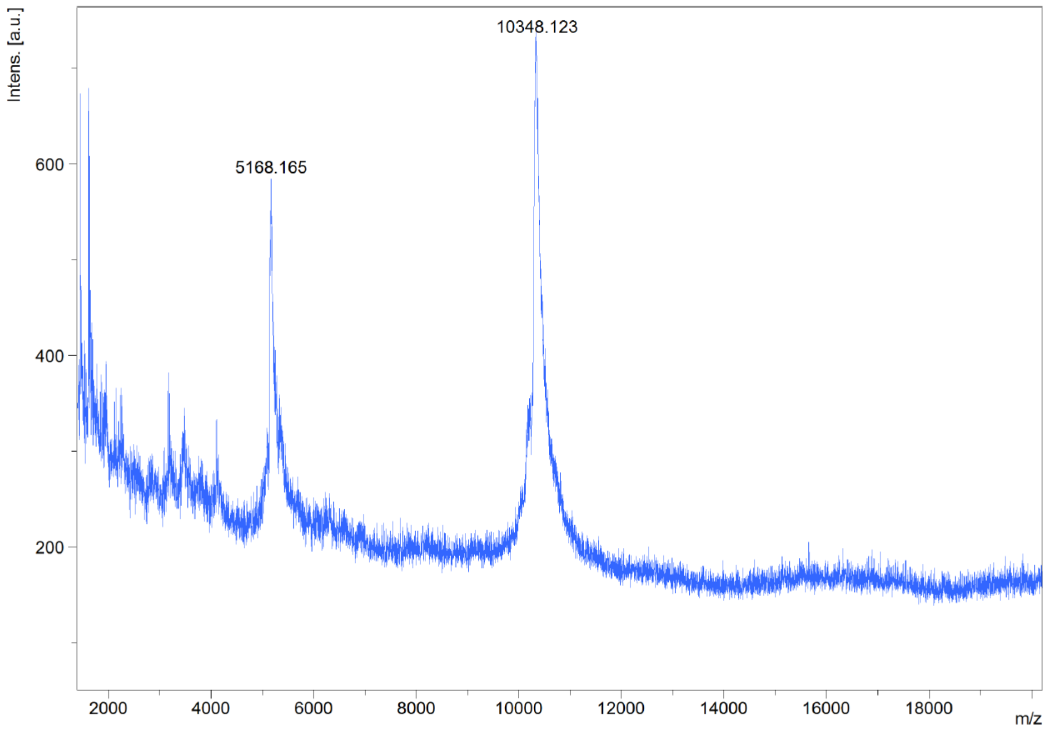
Intens. [a.u.]
600
400
200
10348.123
5168.165
2000
4000
6000
8000
10000
12000
14000
16000
18000
m/z

C) C2

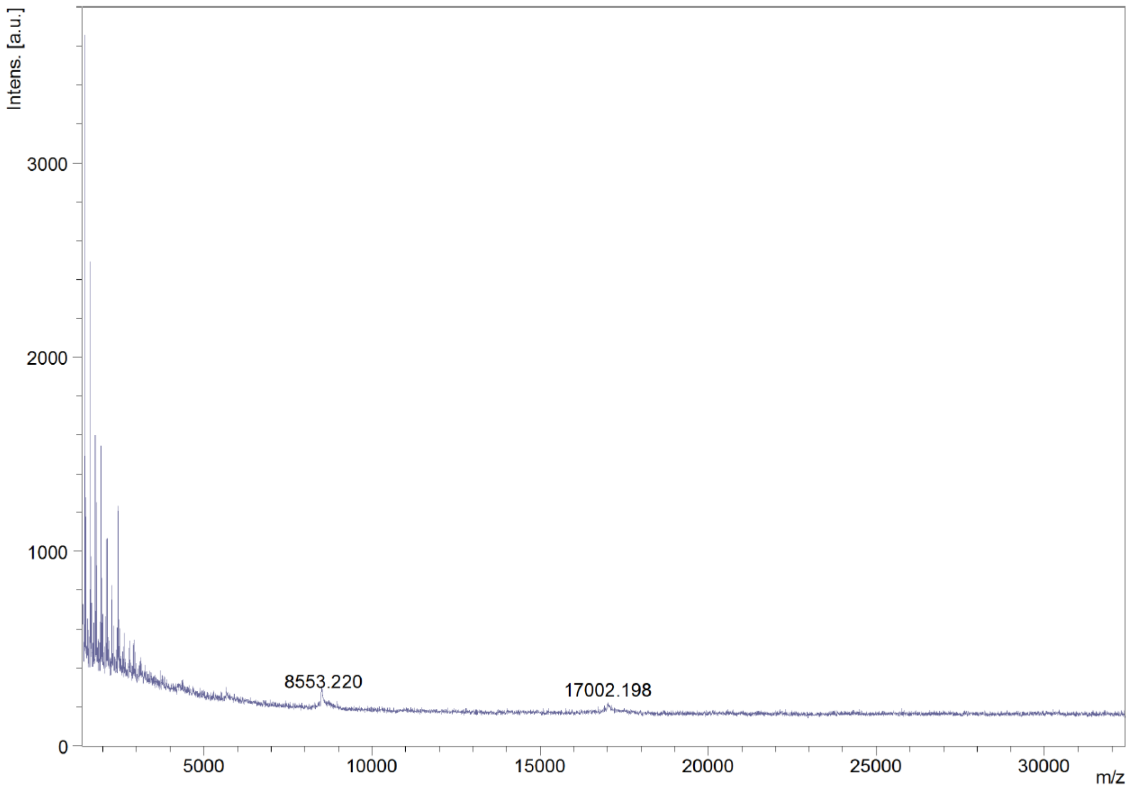

Figure S3. MALDI-TOF MS spectra of conjugates used in this study: C1 (A), C1-Cy7 (B) and C2 (C).

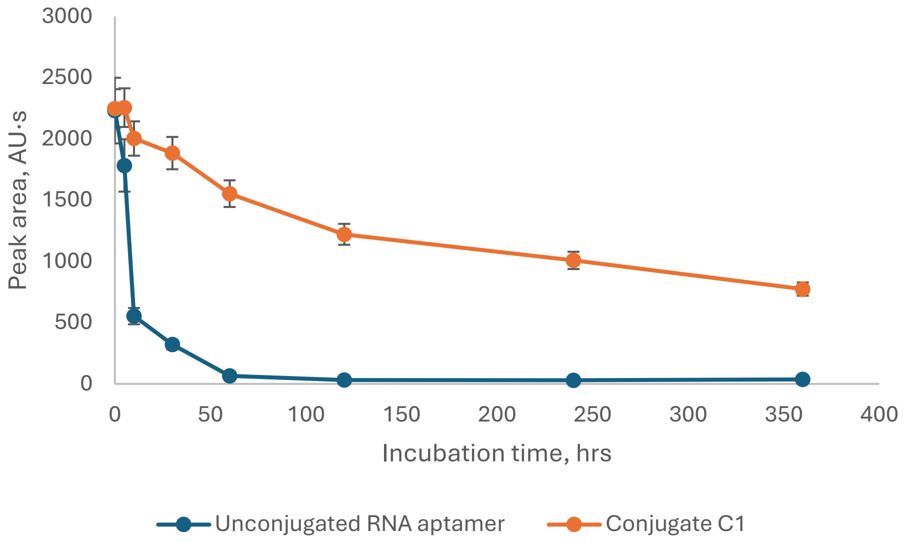

Figure S4. Half-lifetime study of RNA aptamer and C1 conjugate in mouse serum. Each sample was analyzed in duplicate; with mean values for each data point being plotted. Error bar is a CV% between duplicated measurements. AU·s = absorbance units × time.

Critical micelle formation (CMC) concentrations for C1-Cy7 and C2 were determined with dynamic light scattering (DLS). A serial dilution of each conjugate in fresh MQ water was analyzed at 25 °C. Mean particle size values were plotted against sample concentration, and CMC values were determined with first derivative method as 373 μM and 588 μM for C1-Cy7 and C2, respectively.

A)

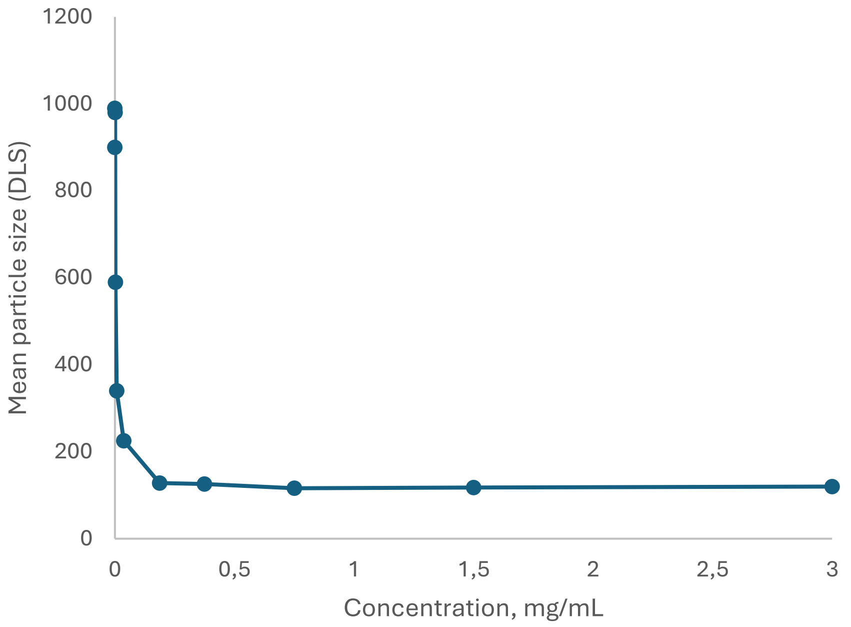

B)

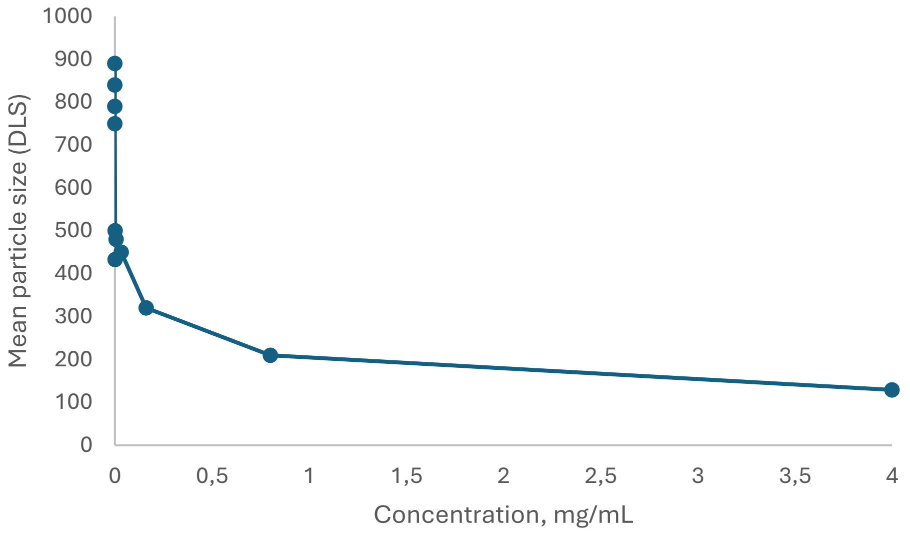

Figure S5. DLS results for C1-Cy7 (A) and C2 (B). CMC concentrations were determined with first derivative method.

6. Tests in human cells

Protocols for the performed cell studies are given below.

Cell Culture

hCMEC/D3 Cell culture:

1. Thaw hCMEC/D3 cells quickly in a 37°C water bath.

2. Transfer cells to a 15 mL conical tube with 10 mL of pre-warmed (Endothelial Cell Growth Medium) EGM-2 medium.

3. Centrifuge at 200 x g for 5 min.

4. Discard the supernatant and resuspend the cell pellet in EGM-2 medium.

5. Plate cells in a culture flask pre-coated with attachment factor (e.g., Poly-L-Lysine).

6. Incubate at 37°C with 5% CO_2, changing the medium every 2 days.

Neuron Cell Line Culture:

1. Thaw SH-SY5Y cells quickly in a 37°C water bath.

2. Transfer cells to a 15 mL conical tube with 10 mL of pre-warmed growth medium.

3. Centrifuge at 200 x g for 5 min.

4. Discard the supernatant and resuspend the cell pellet in growth medium.

5. Plate cells in a culture flask pre-coated with attachment factor.

6. Incubate at 37°C with 5% CO_2, changing the medium every 2 days.

hCMEC/D3 Barrier growing on top of neurons:

1. When hCMEC/D3 and SH-SY5Y cells reach 80-90% confluence, passage cells and seed hCMEC/D3 on top of plates containing SH-SY5Y cells.

2. Replace EGM-2 medium with neuronal differentiation medium (Neurobasal medium with B-27 supplement).

3. Add differentiation factors (Nerve Growth Factor (NGF)).

4. Maintain cells in differentiation medium for 1 week, changing medium every 2 days.

Barrier integrity analysis

Confluent monolayer of hCMEC/D3 was tested with fluorescein isothiocyanate (FITC) dextran fluorescent tracer (Sigma). Solution of FITC dextran (10mM, in 1xPBS) was added to cells in millicell two-chamber wells. Cells were incubated for 2 hours at 37 °C, upper layer was removed; and lower layer was analyzed with fluorescence reader.

Data was processed as follows:

$$P = \frac{(dQ/dt)}{(A \cdot C_0)}$$

Where:

P = permeability coefficient

dQ/dt = amount of tracer passing through the monolayer per unit time

A = surface area of the insert

C_0 = initial concentration of the tracer in the upper chamber

Resulting P values for triplicate measurements were as follows:

1.56 e-6 cm/s

1.60 e-6 cm/s

1.52 e-6 cm/s

hCMEC/D3 passage test

Milli well two chamber plates were used for the assays. TAT-Cy7 and C1-Cy7 samples were added to neuron cells with and without hCMEC/D3 layer in concentration 2 nM, sterile 1xPBS, pH 7.0 (Thermo Fisher). After incubation for 30 min plates were washed 3 times with 100 µL 1xPBS and analyzed with microplate fluorescence reader.

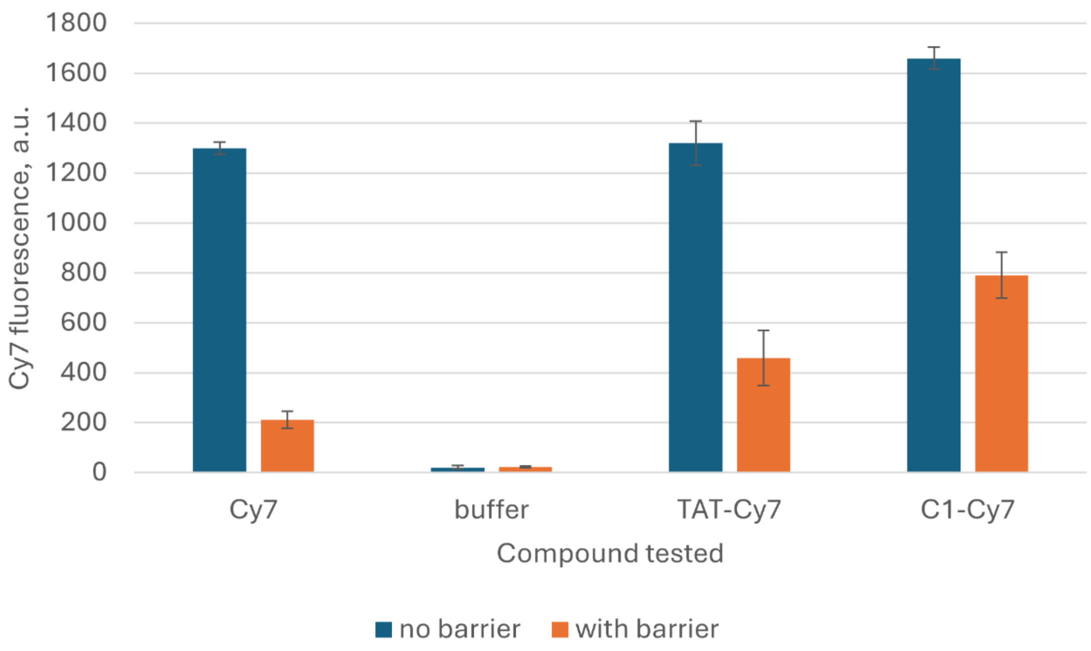

Figure S6. Results of human cell line study for Cy7 and conjugates TAT-Cy7 and C1-Cy7. Bars represent mean values for intra-plate duplicates. p barrier-no barrier = 0.01; p groups = 0.78 (ANOVA). Error bars show CV% for intra-plate triplicate measurements; a.u. = arbitrary units.

Viability assay and miR-125b1 expression in human cells

Efficacy of conjugate was determined in SH-SY5Y and BEAS-2B (Sigma) cells using the tetrazolium microculture (MTT) assay (Sigma Aldrich). Cells were seeded at 4×10^3 cells/well in Falcon TM 96-well plates. Cells were incubated with C2 at desired concentration (50 nM, 150 nM or 450 nM) or PBS for 24h, in intra-plate triplicates. After adding 20 µM MTT solution to each well containing 200 µM medium, the cells were incubated at 37 °C for additional 4 hours. Next, 50 µL DMSO was added to each well and incubated for 30 minutes to stop the reaction. A microplate reader was used to measure the formazan produced by the cells at 570 nm. The percentage of absorbance of the sample cells divided by that of the control cells was used to estimate cell viability.

MiR-125b1 expression in cells was tested with RT-qPCR using Thermo Fisher kit (ID 000449), following the manufacturer's protocol and with the provided calibrator. Reactions were performed using Roche Light Cycler 480. Each reaction was carried out in intra-plate duplicate.

Table S3. Expression levels of miR-125b1 in cells.

Cell line	miR-125b1, ng/mL cDNA equivalents; mean of duplicate measurement/CV, %
SH-SY5Y	32.1/5.8
BEAS-2B	7.4/6.5

A)

B)

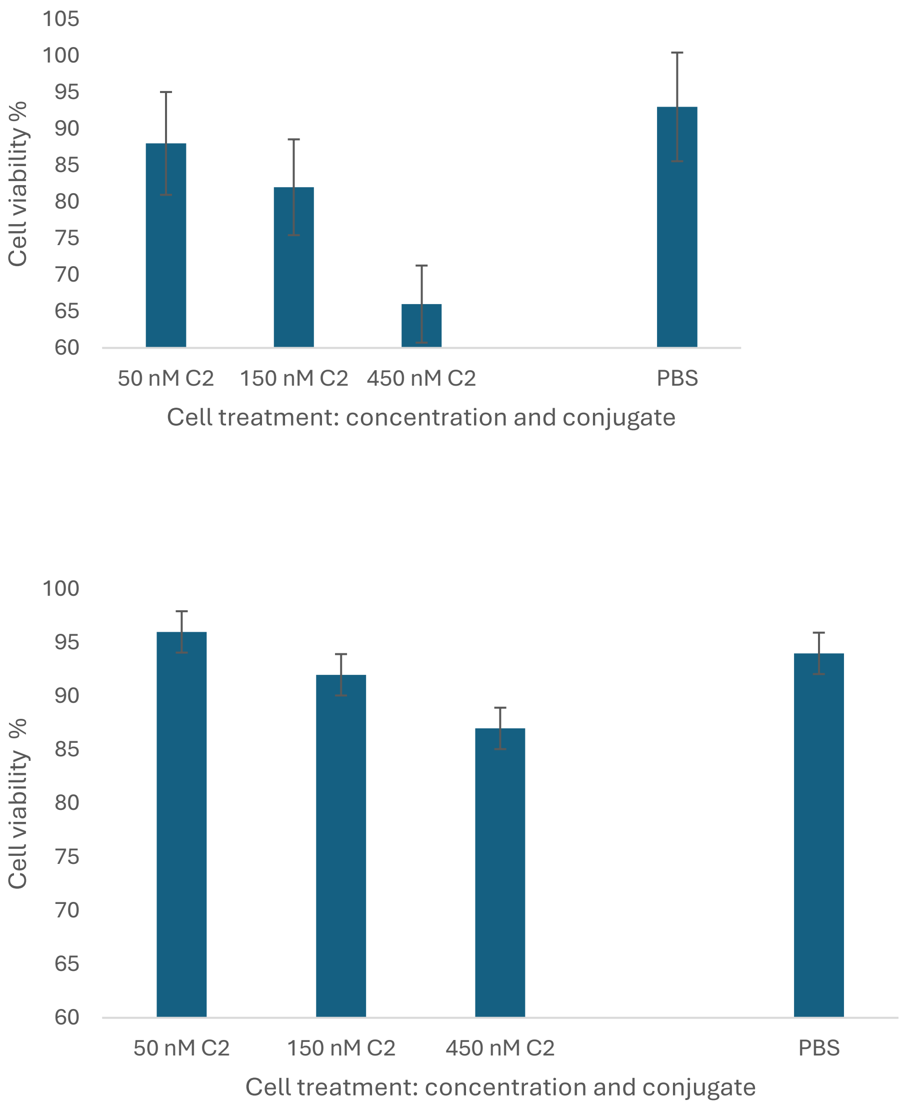

Figure S7. Results of cell viability tests (MTT): A) SH-SY5Y; B) BEAS-2B. Each data point is a mean value of intra-plate triplicate. Error bars show CV% for intra-plate triplicate measurements.

7. Studies in vivo

Studies were conducted in accordance with EU FELASA legislation for ethical animal research and under permit 2023-15-0201-01479 by AIRD Denmark to PI Astakhova.

Federation of European Laboratory Animal Science Associations (FELASA) trained personnel conducted in vivo studies in accordance with EU legislation for ethical animal research.

7-Week old Balb/cJ mice (male and female) were purchased from Janvier, France. Mice were randomized at arrival and accommodated for 1 week prior to study initiation with unrestricted access to water and standard diet. Study groups were as follows:

- C1-Cy7, 20 mg/kilo, n=5 male, n=2 female
- TAT-Cy7, 20 mg/kilo, n=2 female
- C2, n=3 male, n = 3 female
- Unconjugated anti-miR 125b1, n=3 male, n = 3 female
- Not treated controls, n= 3 male, n= 3 female

For injections, stocks of conjugates and controls were prepared and sterile filtered in 1xPBS at concentrations 4mg/mL. Volumes injected were adjusted to mice weight and the required final dose were approx. 100 µL/mouse.

Mice were dosed i.v. into tail vein, anesthetized with isoflurane/oxygen, shaved and analyzed longitudinally with whole-body fluorescence imaging.

Fluorescence imaging was conducted for Cy7-labelled compounds and 1x PBS control of each sex using U-CT(UHR)-OI(FLT/BLT) scan system from MILabs (Utrecht, The Netherlands). Ultra-high-resolution computed tomography optical imaging (U-CT-OI) device was used under fluorescence mode and the following parameters: exposure time, 60000 ms; iris setting, f/2.0; Preamplification, 2x; Binning, 2x2; Emission, 785 nm; Excitation, 710 nm. Fluorescence units are given in to electron flux units (e-/s). After injection, mice were scanned at points 0 , 1, 3, 6 hours and 1, 2, 3, 4, 7 days.

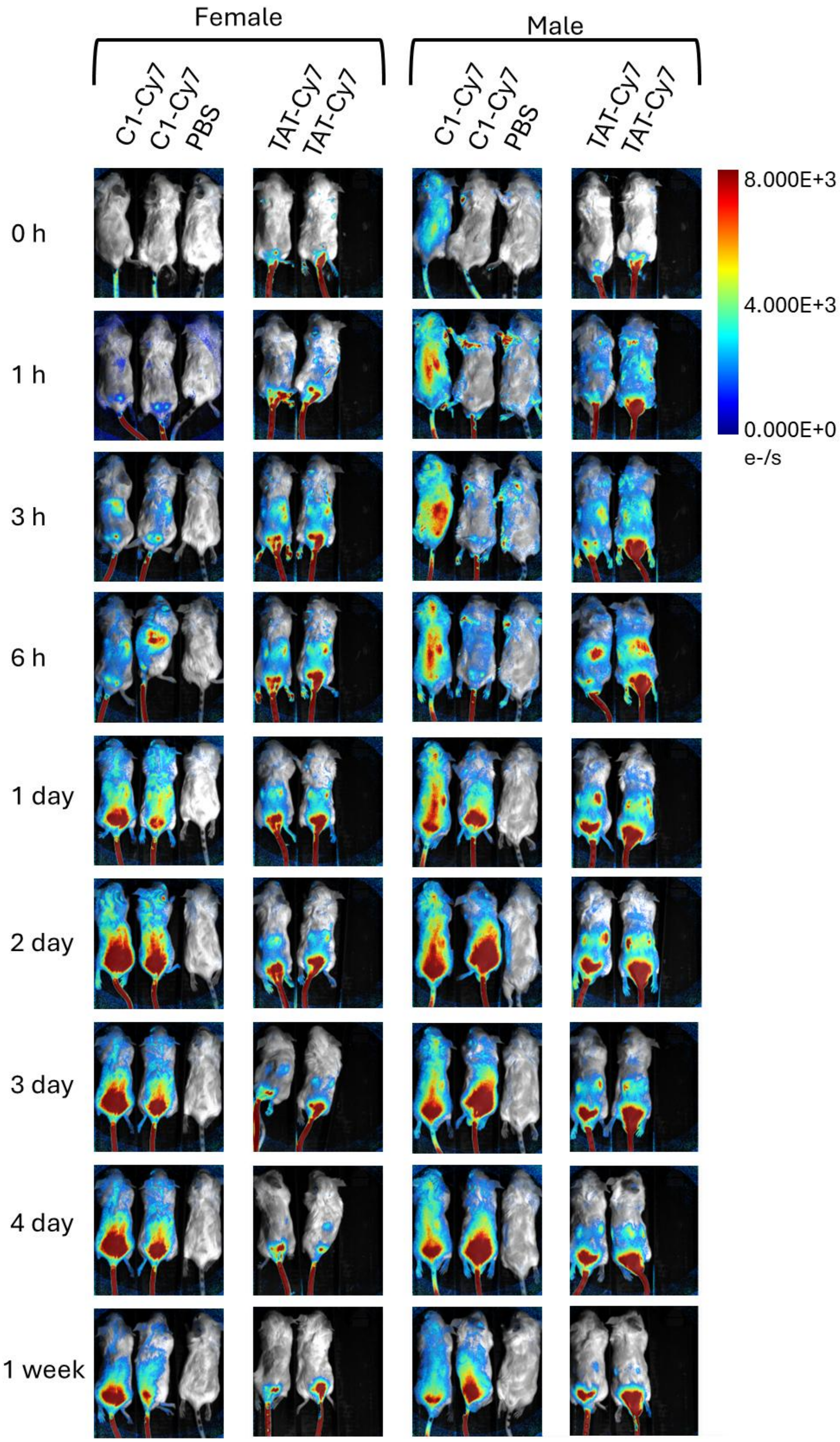

Figure S8. Longitudinal fluorescence scanning for C1-Cy7 (female, n = 2; male, n= 2, along first and third column, respectively), TAT-Cy7 (female, n = 2; male, n= 2, in second

and fourth column, respectively) and phosphate buffer saline (PBS) control groups (female, n = 1; male, n= 1, in first and third column, respectively). Both C1-Cy7 and TAT-Cy7 groups dosed 20 mg/kg. Fluorescence intensity normalized to 8000 e-/s. e-/s = electron flux.

Three mice from the C1-Cy7 male group were scanned at points 1, 6, 24 and 48 hours, then terminated after data point 48 h and their organs scanned after dissection for biodistribution studies.

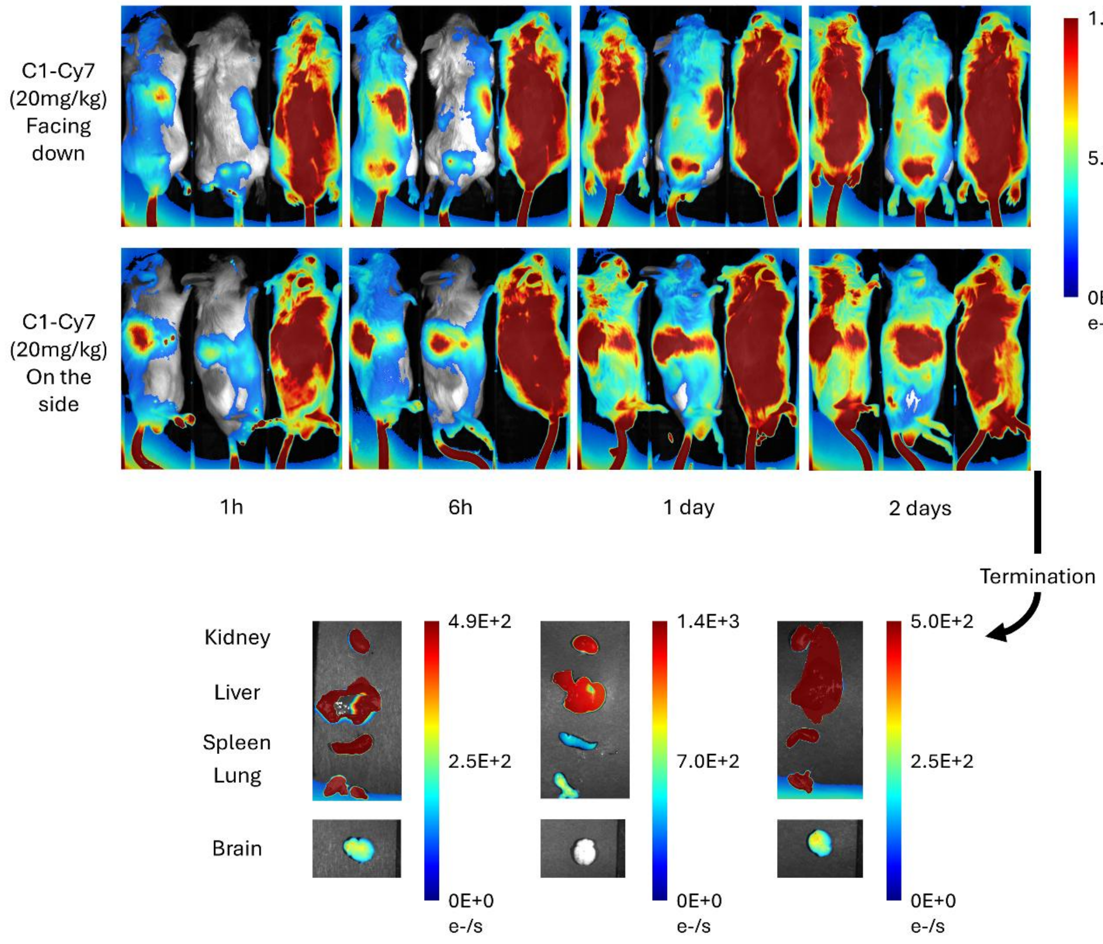

Figure S9. Longitudinal distribution of C1-Cy7 administered at 20 mg/kg dose including harvested organs. Measured by whole body fluorescence scanning, displaying n= 3 male mice laying on their stomach (top row) and on their side (bottom row). The disposition of the anaesthetized mice (fluorescence intensity normalized to 1000 e-/s) is not changed along the data points or orientation display. After termination, organs of each mouse were extracted and arranged in the same order than in the anaesthetized scans (lower section of the figure).

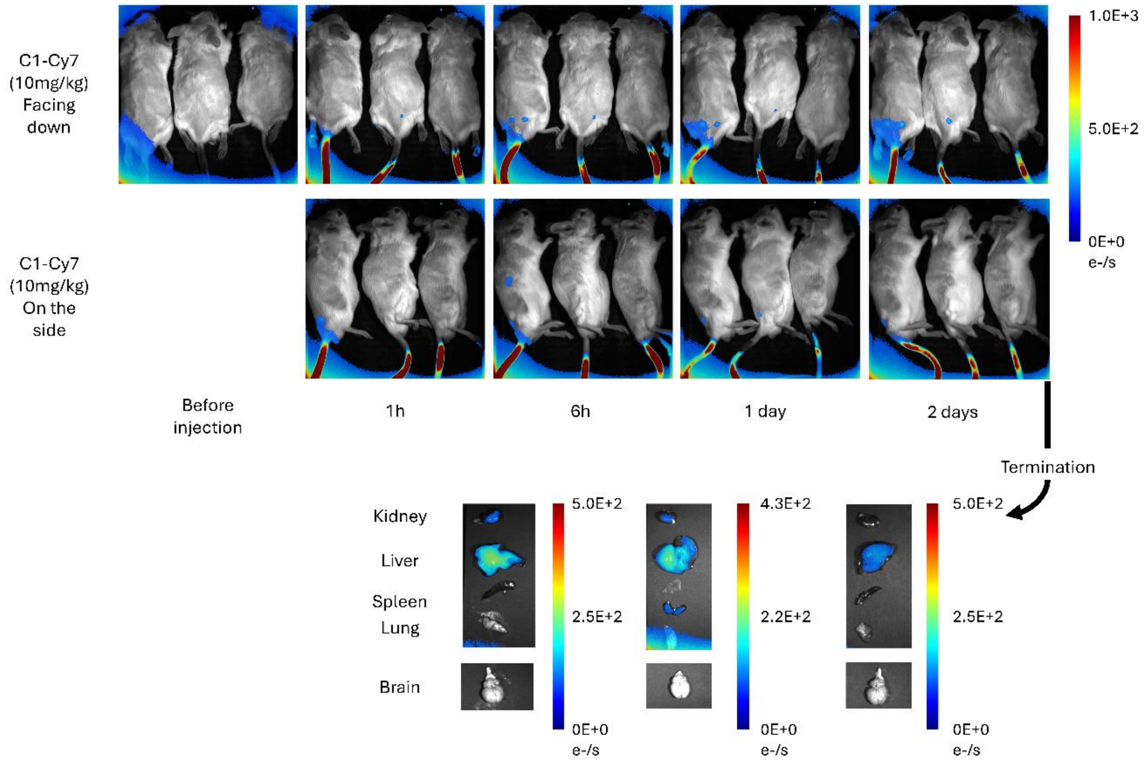

Figure S10. Control images: longitudinal distribution of C1-Cy7 administered at 10mg/kg dose including harvested organs, measured by whole body fluorescence scanning, displaying n = 3 male mice laying on their stomach (top row) and on their side (bottom row). The disposition of the anaesthetized mice (fluorescence intensity normalized to 1000 e-/s) is not changed along the data points or orientation display. After termination, organs of each mouse were extracted and arranged in the same order than in the anaesthetized scans (lower section of the figure).

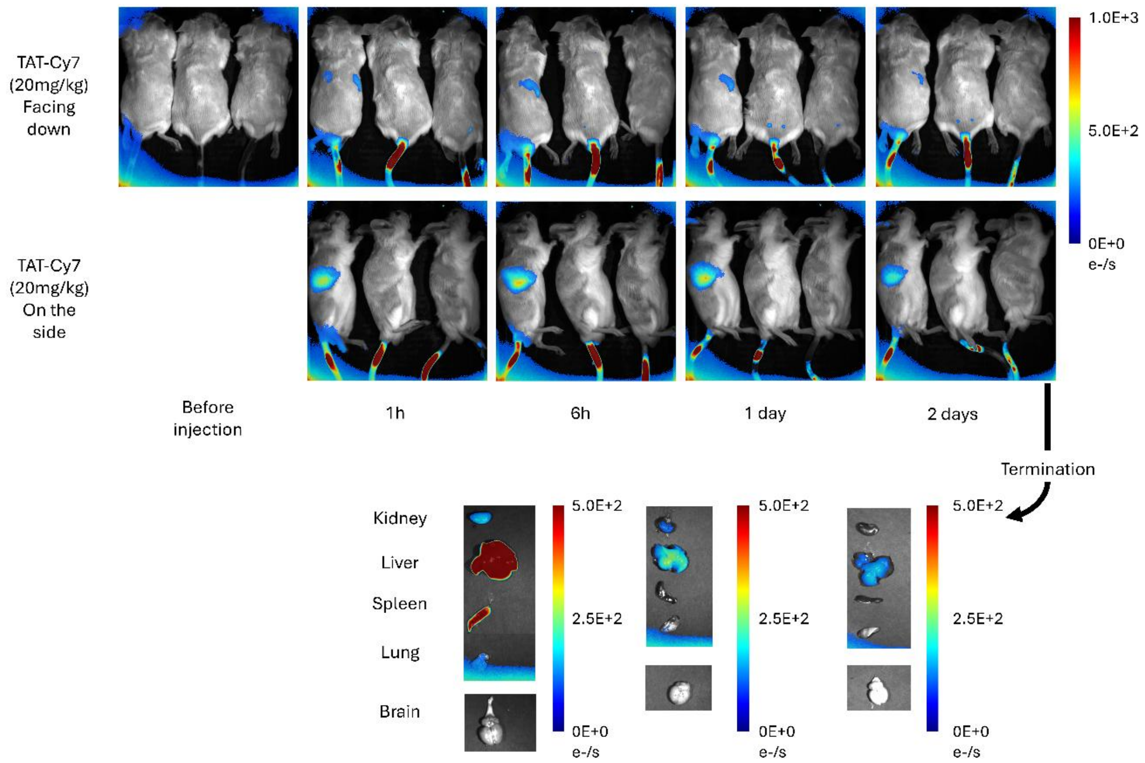

Figure S11. Control images: longitudinal distribution of TAT-Cy7 administered at 20mg/kg dose including harvested organs. Measured by whole body fluorescence scanning, displaying n = 3 male mice laying on their stomach (top row) and on their side (bottom row). The disposition of the anaesthetized mice (fluorescence intensity normalized to 1000 e-/s) is not changed along the data points or orientation display. After termination, organs of each mouse were extracted and arranged in the same order than in the anaesthetized scans (lower section of the figure).

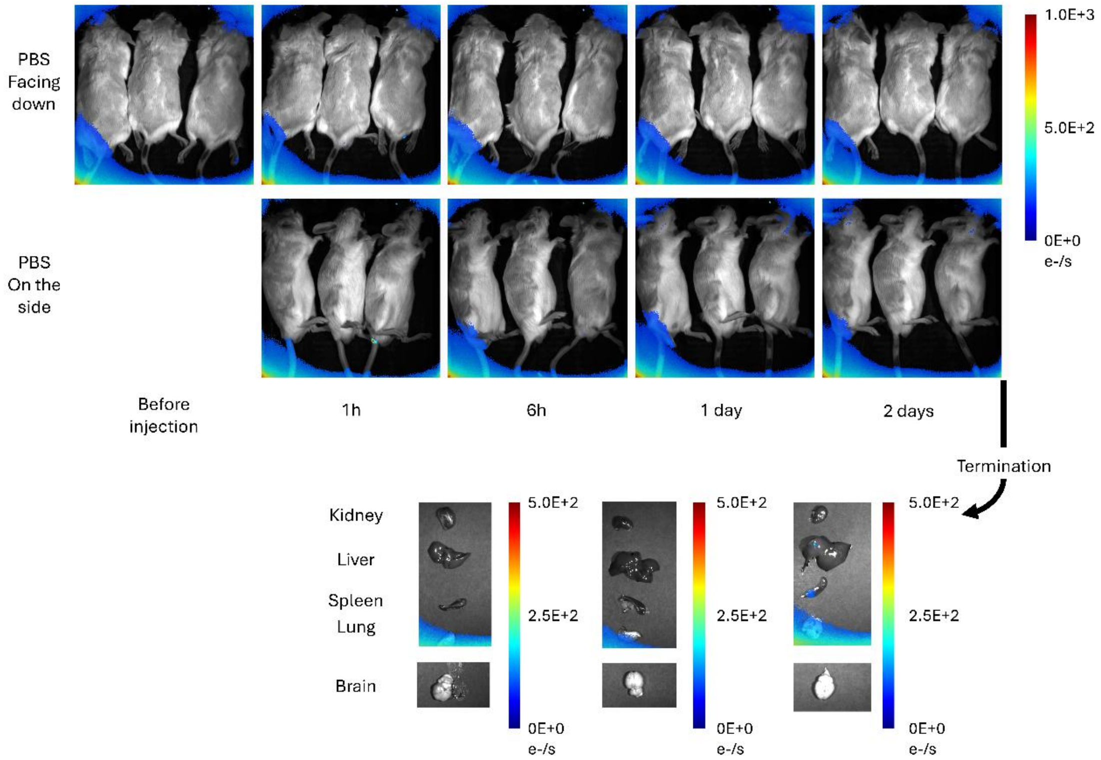

Figure S12. Control images: Pre-injection scanning and mice treated with PBS; incl. harvested organs. Measured by whole body fluorescence scanning, displaying n = 3 male mice laying on their stomach (top row) and on their side (bottom row). The disposition of the anaesthetized mice (fluorescence intensity normalized to 1000 e-/s) is not changed along the data points or orientation display. After termination, organs of each mouse were extracted and arranged in the same order as in the anaesthetized scans (lower section of the figure).

Blood samples were taken longitudinally from tongue vein. Serum was obtained as follows:

1. Whole blood collected in a covered test tube
2. Blood let clot by leaving it undisturbed at room temperature for about 15–30 minutes.
3. Blood centrifuged at 2,000 x g for 10 minutes in a refrigerated centrifuge
4. The resulting supernatant is carefully removed using a Pasteur pipette
5. Serum samples stored in aliquots at –20°C for 2-month time and at –80°C for prolonged time

Mice were sacrificed with CO_2 followed by cervical dislocation.

Mice were harvested; spleen, liver, kidney, lungs and brain were weight in. Samples of organs were transferred into formaldehyde for further paraffin embedding.

Paraffin embedded organs were spleen, liver, kidney, lungs and brain.

Samples of brain (frontal lobe areas) were cryo-preserved in sterile tubes with liquid nitrogen and stored at – 80 °C.

8. qPCR measurements

Cryo-preserved tissue samples were processed with tissue homogenizer. DNA was extracted with Qiagen DNeasy Blood & Tissue Kit. DNA from sera samples was obtained with same kit.

Extended qPCR primers were designed with Python script and obtained from IDT. Primer sequences were as follows:

Forward: 5'-TCA CAA GTT AGG GTC TCA G-3'

Reverse: 5'- TCT C+AG GGA TCC C+AG-3'

Where +N is locked nucleic acid modification to increase specificity and affinity.

SYBR Green PCR Master Mix was applied (ThermoFisher). Reactions were performed using Roche Light Cycler 480. Each reaction was carried out in intra-plate duplicate.

Serial dilution of unmodified DNA anti-miR was used to generate standard curve.

Following protocol was used for qPCR quantification of DNA from mouse tissue samples and sera:

1. Prepare qPCR Reaction Mix

10 μL of SYBR Green PCR Master Mix

1 μL of forward primer (10 μM)

1 μL of reverse primer (10 μM)

1 μL of template DNA (2 ng/μL)

Nuclease-free water to a final volume of 20 μL

2. qPCR Cycling Conditions

Following conditions were used:

- Initial denaturation: 95°C for 2 minutes
- Denaturation: 95°C for 20 seconds
- Annealing: 58°C for 20 seconds
- Extension: 72°C for 30 seconds
- Number of cycles: 38

3. After the final extension, perform a melt curve analysis by gradually increasing the temperature from 65°C to 95°C and measuring fluorescence continuously.

4. Cycle threshold (ct) values obtained and converted to ng/μL DNA using calibration curve; CV% determined from intra-plate duplicates.

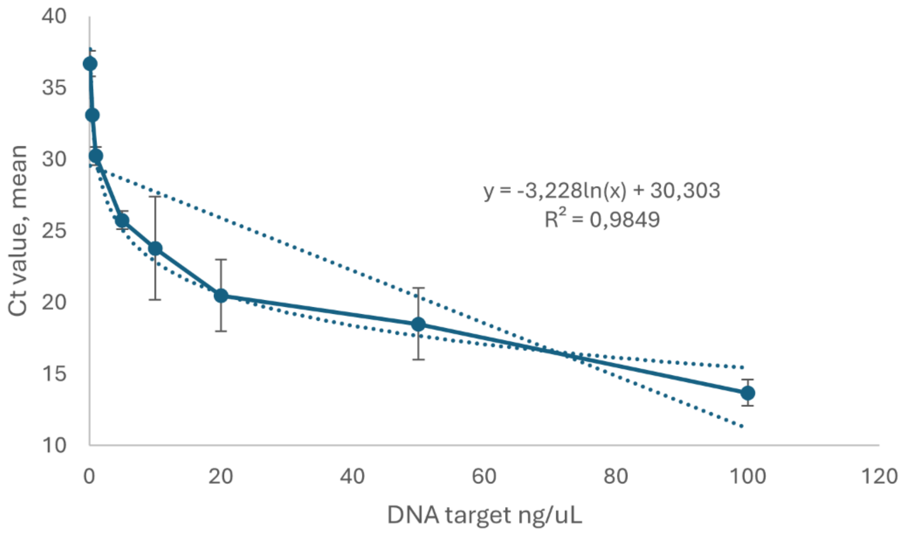

Figure S13. Calibration curve for anti-miR detection with qPCR protocol; Ct = cycle threshold Error bars show CV% for intra-plate duplicate measurements. Linear curve fitting is shown.

Table S4. DNA levels (ng/µL) determined in mouse organs 2d post-administration of C2. Data shown for biological triplicates (1,2,3) and assay triplicates (1,2,3).

	Male brain	Male liver	Male kidney	Female brain	Female liver	Female kidney
1-1	6	0.6	0.6	4.3	1.3	3.1
1-2	5	0.9	0.9	3.5	2.2	1.2
1-3	6.6	1.1	1.1	5.6	2	0.6
2-1	11	1.3	1.3	3.3	1.3	0.8
2-2	5.4	1.7	1.7	4.3	1.7	1.3
2-3	7.8	2	2	2.1	2	2
3-1	5.4	3	3	3.5	3	3
3-2	6	2	2	3	3.4	2
3-3	9.2	4	2	4	3	1

Table S5. Anti-miR levels for conjugate C2 determined longitudinally in brain and serum samples: qPCR results for male (m1-3) and female (f1-3) mice, in intra-plate duplicates.

MALE								FEMALE						
	**Brain level**							**Brain level**						
Time (h)		m1	m1	m2	m2	m3	m3		f1	f1	f2	f2	f3	f3
6	**0.40**	0.40	0.55	0.70	0.21	0.32	0.20	**0.11**	0.03	0.10	0.09	0.12	0.18	0.11
24	**4.22**	4.60	4.00	2.40	3.10	5.70	5.50	**1.08**	1.11	1.21	1.18	2.40	1.45	0.32
48	**5.13**	4.00	3.21	3.60	6.80	5.40	7.80	**2.05**	2.21	2.00	3.60	2.10	1.30	1.10
168	**0.06**	0.01	0.03	0.12	0.07	0.09	0.01	**0.038**	0.03	0.02	0.01	0.10	0.04	0.02
	**Serum level**							**Serum level**						
6	**10.19**	14.00	12.20	11.21	10.00	6.00	7.70	**3.38**	3.20	3.00	2.60	2.10	4.40	5.00
24	**2.48**	3.21	3.00	2.11	2.40	1.13	3.00	**2.10**	1.80	2.00	2.10	1.40	3.20	2.10
48	**0.79**	1.10	0.89	0.45	0.88	1.12	0.32	**0.45**	0.45	0.40	0.78	0.44	0.32	0.30
168	**0.07**	0.05	0.08	0.11	0.03	0.04	0.11	**0.07**	0.02	0.14	0.03	0.04	0.11	0.09

Table S6. Anti-miR levels (unconjugated) determined longitudinally in brain and serum samples: qPCR results for male (m1-3) and female (f1-3) mice, in intra-plate duplicates.

MALE								FEMALE						
	**Brain level**							**Brain level**						
Time (h)		m1	m1	m2	m2	m3	m3		f1	f1	f2	f2	f3	f3
6	0.05	0.05	0.07	0.09	0.03	0.04	0.03	**0.01**	0.00	0.01	0.01	0.02	0.02	0.01
24	0.30	0.33	0.29	0.17	0.22	0.41	0.39	**0.15**	0.14	0.11	0.15	0.30	0.18	0.04
48	0.23	0.18	0.15	0.16	0.31	0.25	0.35	**0.26**	0.10	0.09	0.16	0.10	0.06	0.05
168	0.02	0.02	0.02	0.02	0.02	0.02	0.02	**0.01**	0.03	0.00	0.00	0.01	0.01	0.00
	**Sera level**							**Sera level**						
6	1.27	1.75	1.53	1.40	1.25	0.75	0.96	**0.42**	0.40	0.38	0.33	0.26	0.55	0.63
24	0.31	0.40	0.38	0.26	0.30	0.14	0.38	**0.26**	0.23	0.25	0.26	0.18	0.40	0.26
48	0.53	0.37	0.30	0.15	0.29	0.37	0.11	**0.06**	0.15	0.13	0.26	0.15	0.11	0.10
168	0.02	0.02	0.03	0.04	0.01	0.01	0.04	**0.01**	0.01	0.05	0.01	0.01	0.04	0.03

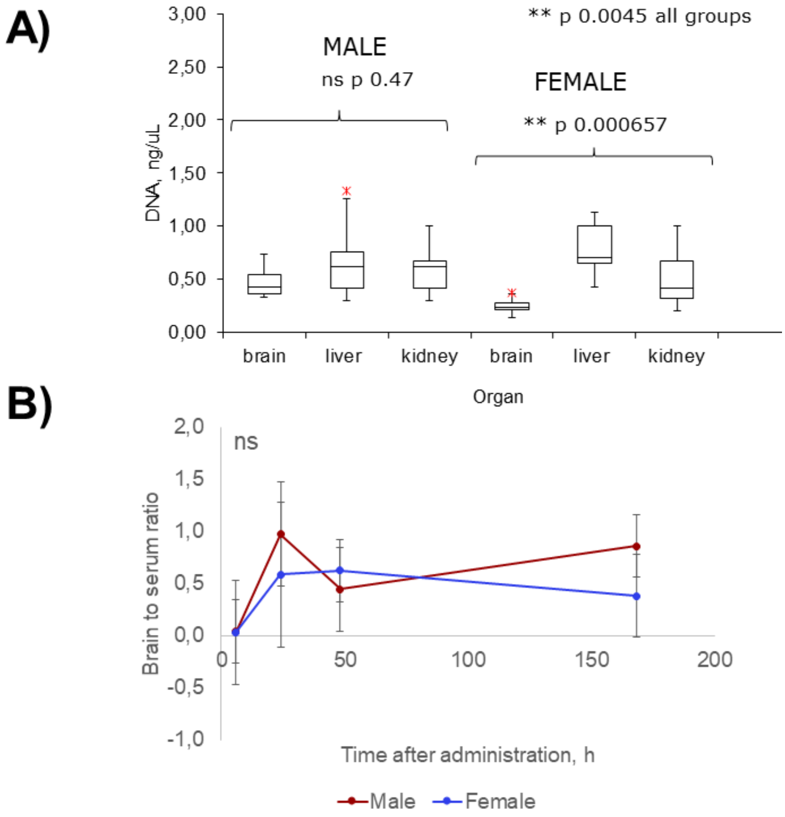

Figure S14. Results for biodistribution study of unconjugated anti-miR 125b1: A) Boxplot with outliers for organ accumulation in male and female mice; and B) brain-to-serum ratios measured with qPCR (B). Ns= not significant; ** = 99% significance level. Analyses made with ANOVA. (B) Error bars show CV% for biological triplicates. Data presented for biological triplicates and intra-plate assay duplicates.

For TfR1 expression study, brain tissues of five male and five female untreated Balb/cJ mice were used. Tissue samples were processed and analyzed with qPCR using Bio-Rad PrimePCR product for mouse *TfR1/Tfrc*. RNA was extracted from three different areas per sample and brain area, taken in the dorsal direction. Samples from the same animal and brain area were then pooled; RNA was extracted with Qiagen RNAeasy kit following manufacturer's protocol and quantified using Nanodrop. A total of 150 ng RNA was treated with DNAse I enzyme to remove any potential genomic contamination, mixed with nuclease free water and 10x reaction buffer and incubated for 30 min in a T100 Thermal cycler (Bio-rad) at 37 °C. All samples were then treated with ethylenediaminetetraacetic acid (EDTA) for 10 min at 65 °C to stop the reaction. Complementary DNA was generated using the RevertAid H Minus First Strand cDNA Synthesis Kit, which consisted of random hexamer primer, oligo primer, 10 mM dNTP, 5x reaction buffer, and nuclease free water. This was mixed with Maxima H Enzyme mix and 100 ng of DNase-treated RNA and the samples run in the thermal cycler using the following thermal profile: 10 min at 25 °C, 15 min at 50 °C, and 5 min at 85 °C. The RT-qPCR was carried out by preparing a master mix containing the TaqMan Multiplex MasterMix and the Taqman primers/probes for *Tfrc* and

then adding 1.7 ng of sample into each well. The samples were run on a Roche Light Cycler with the thermal profile: 50 °C for 2 min and 95 °C for 20 s, followed by 40 cycles of 95 °C for 1 s and 60 °C for 20 s. After the run, the samples were cooled down to 20 °C. The relative mRNA expression was calculated using the ΔΔCT method and average of all measured samples as a normalization parameter.

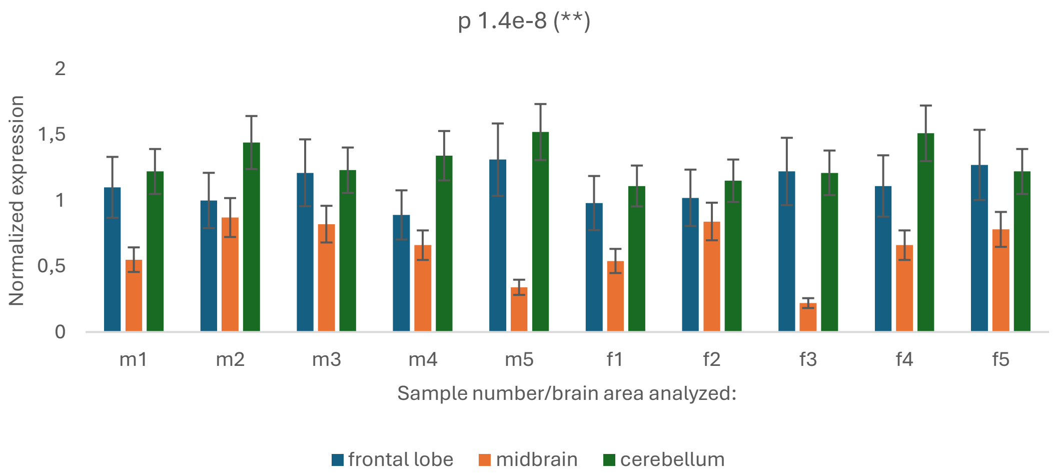

Figure S15. Normalized expression levels of TfR1 in healthy untreated Balb/cJ mice brains, determined with RT-qPCR; m1-5 males, f1-5 females. Analyses done in intra-plate duplicates, with mean CV=12% (shown as error bars on the graph). ** = 99% significance level.

9. Histopathology

Paraffin-embedded tissue slices were stained with Hematoxylin and Eosin (H&E), and imaged with Leica LS8 microscope using 20x objective.

Details on histopathological assessment for each organ and treatment group are given in the following section.

For fluorescence in situ hybridization (FISH), tissue has been fixed, permeabilized with Triton X-100 (0.1% for 15 minutes), blocked with 1%BSA for 2 hours and stained at +4 °C overnight using 4',6-diamidino-2-phenylindole (DAPI) or antibody dilutions as suggested by manufacturers.

Summary of Fluorescent Channel Assignments:

-staining 1:

- **DAPI**: Blue channel (450 nm emission), Thermo Fisher Scientific (DAPI, Catalog # D1306)

-staining 2:

- **a-CD31**: Green channel (Alexa Fluor 488, 519 nm emission), BioLegend (Anti-CD31, Catalog # 102502).
- **Neuron Marker**: Red channel (Alexa Fluor 555, 565 nm emission), MilliporeSigma (Anti-NeuN, Catalog # MAB377)

The images were captured using Leica LS8 microscope. Imaging has been done independently for DAPI (setting 1) and multiplex 2 setting (channels for Alexa Fluor 488, 555 and Cy7).

Magnification has been adjusted to assess the tissue morphology of the brain (blood vessels; neurons); while detecting signal of Cy7-labelled conjugates. Different background colors of the images reflect the required changes in gain parameter of the microscope adjusted to capturing individual images.

a)

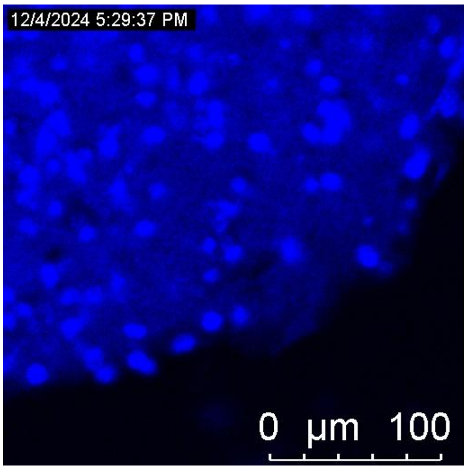

b)

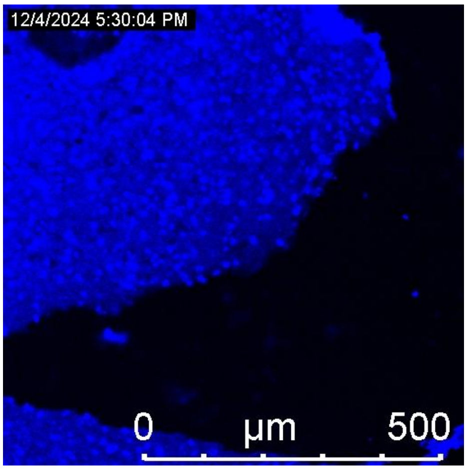

c)

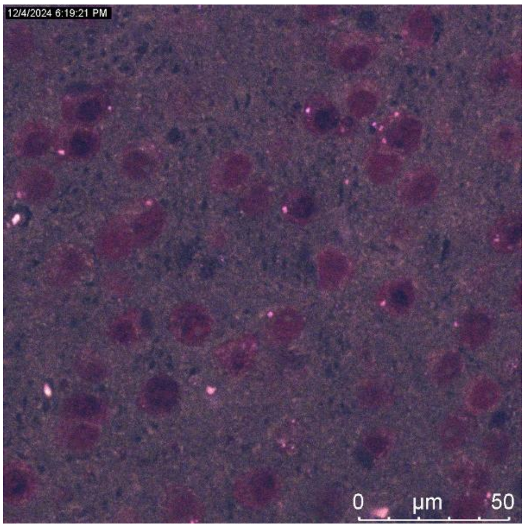

Figure S16. Fluorescence in situ hybridization (FISH) images of male Balb/cJ mice treated with C1-Cy7 20 mg/kg (additional data). Brain tissue for mouse 1 and 2 stained with 4',6-diamidino-2-phenylindole (DAPI) (A and B, respectively); Brain tissue images from mouse 2 stained with a-CD31 and a-NeuN; visualized with multiplex setting 2 (C). Cy7 is seen in white; CD31 (blood vessels) and NeuN (neurons) are visualized in dark blue and purple, respectively. Data for mouse 1 is given in the main paper.

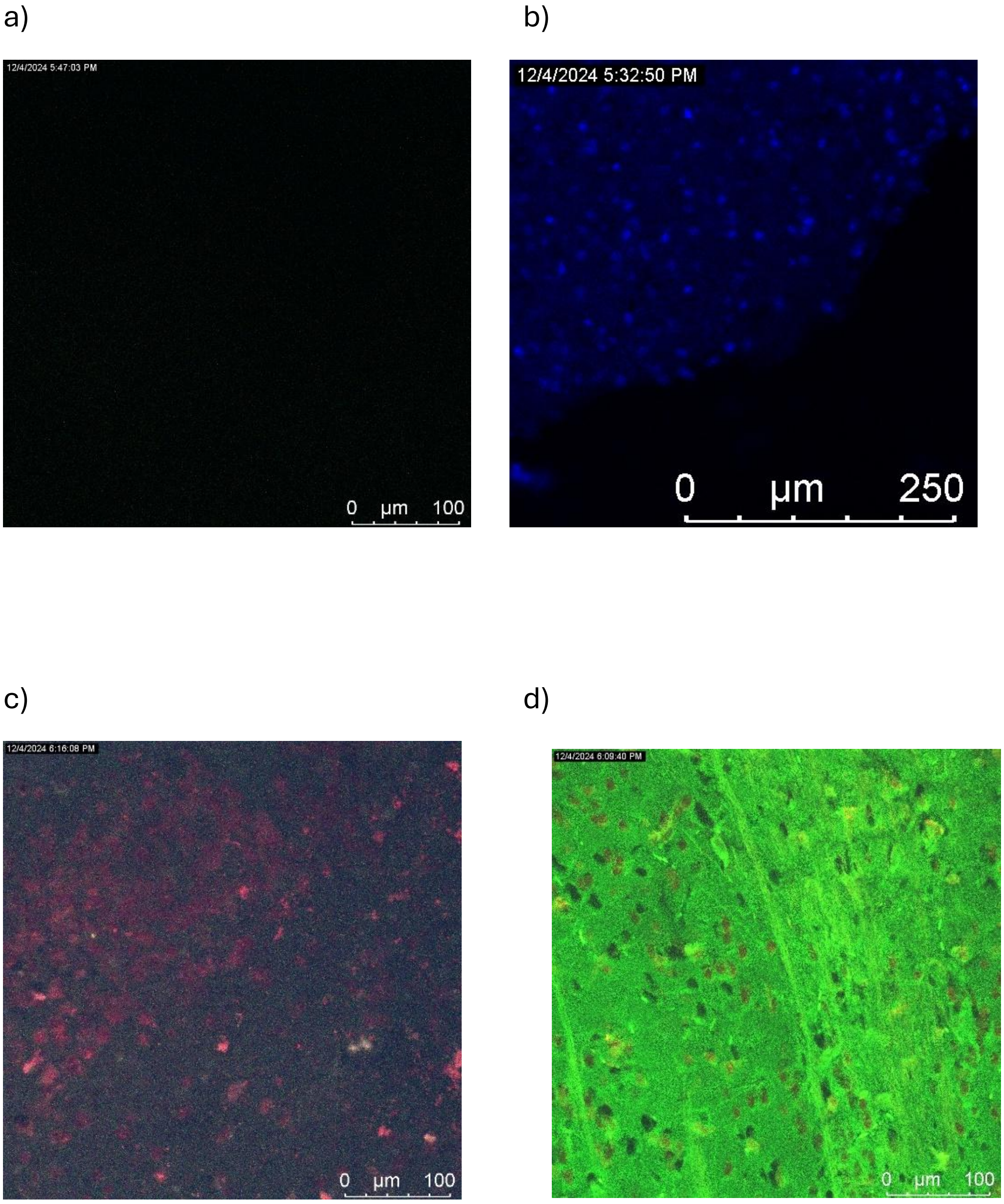

Figure S17. Fluorescence in situ hybridization (FISH) images of brain tissue obtained from control mice: A) unstained slide treated with PBS imaged with multiplex setting 2; Tissue of mouse treated with TAT Cy7 20 mg/kg imaged at (4',6-diamidino-2-phenylindole) DAPI (B) and multiplex setting 2 (C). D) Stained brain tissue of mouse treated with PBS imaged with multiplex setting 2. Red and dark green/black are stained neurons and blood vessels, respectively.

9.1 Pathological Features in Kidney and Liver

Kidney pathology of glomeruli is seen as hypertrophy, mesangial expansion, sclerosis (indicated by increased matrix deposition), or glomerular basement membrane thickening.[36-38] Glomerular pathology scoring was done as follows:

- 0: Normal glomeruli.
- 1: Mild glomerular hypertrophy or sclerosis (<25% of glomeruli affected).
- 2: Moderate hypertrophy or sclerosis (25-50%).
- 3: Severe hypertrophy or sclerosis (>50%).

Pathology of kidney tubules is seen as tubular dilation, atrophy, necrosis, or casts. Scoring was done as follows:

- 0: Normal tubules.
- 1: Mild tubular dilation or degeneration.
- 2: Moderate dilation, atrophy, or necrosis.
- 3: Severe necrosis or widespread atrophy.

Last, we evaluated assess for inflammation (presence of immune cells), fibrosis (collagen deposition), or edema.

Scoring was done as follows:

- 0: Normal interstitium.
- 1: Mild interstitial inflammation or fibrosis.
- 2: Moderate inflammation or fibrosis.
- 3: Severe fibrosis, inflammation, or edema.

Minor pathology affecting kidney glomeruli and pointing on inflammation is seen in males received C1-Cy7; TAT-Cy7 is affecting negatively kidney in both females and males with rather high score (4 total).

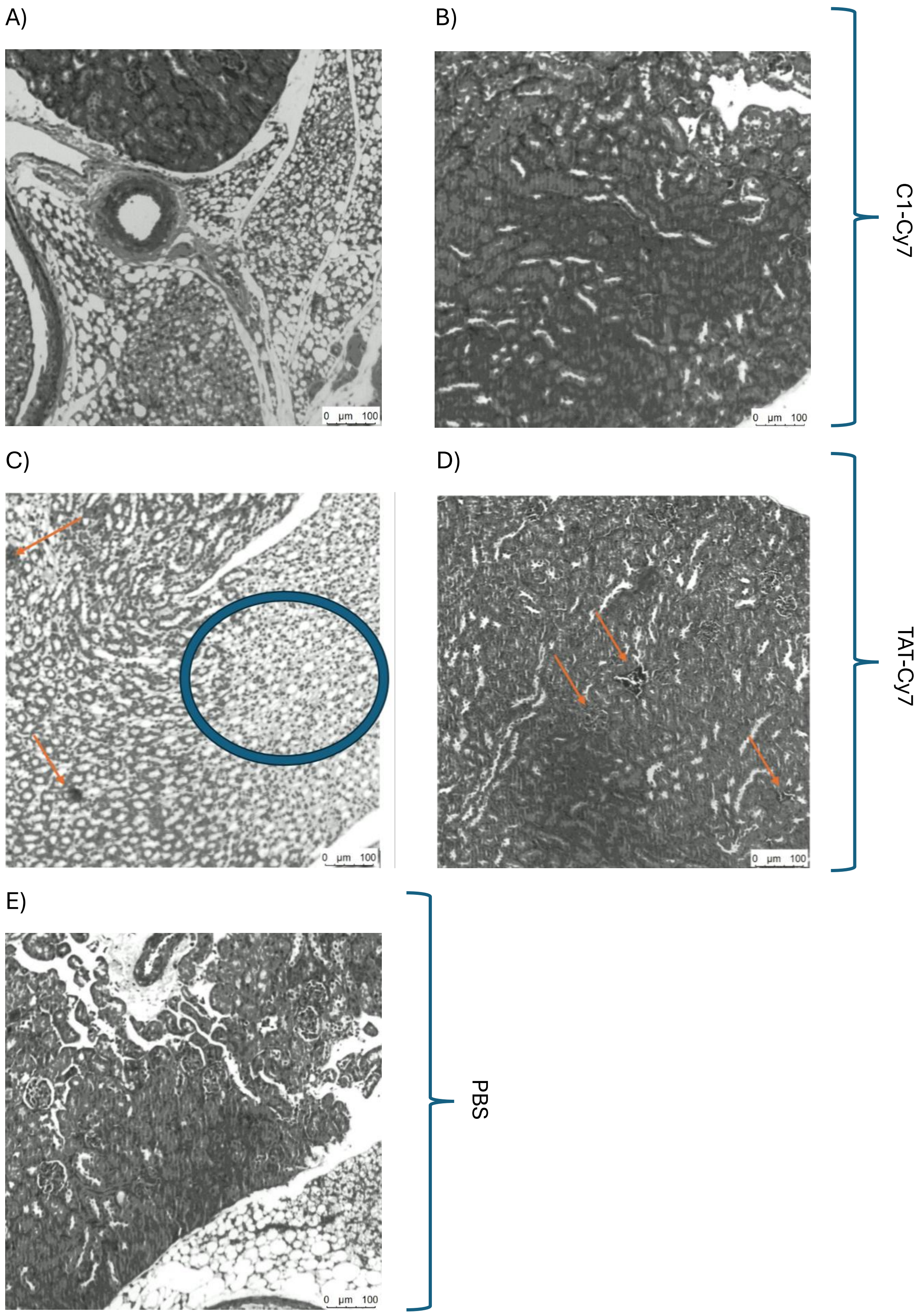

Figure S18. Light micrograph images of H&E stained kidney tissues from female mice treated with C1-Cy7 (A,B), TAT-Cy7 (C,D)and phosphate buffer saline (PBS) (E). Figs C,D: matrix deposition (dark areas; shown with orange arrows) and presence of immune cells (shown in blue circles) detected. n=2 per study group.

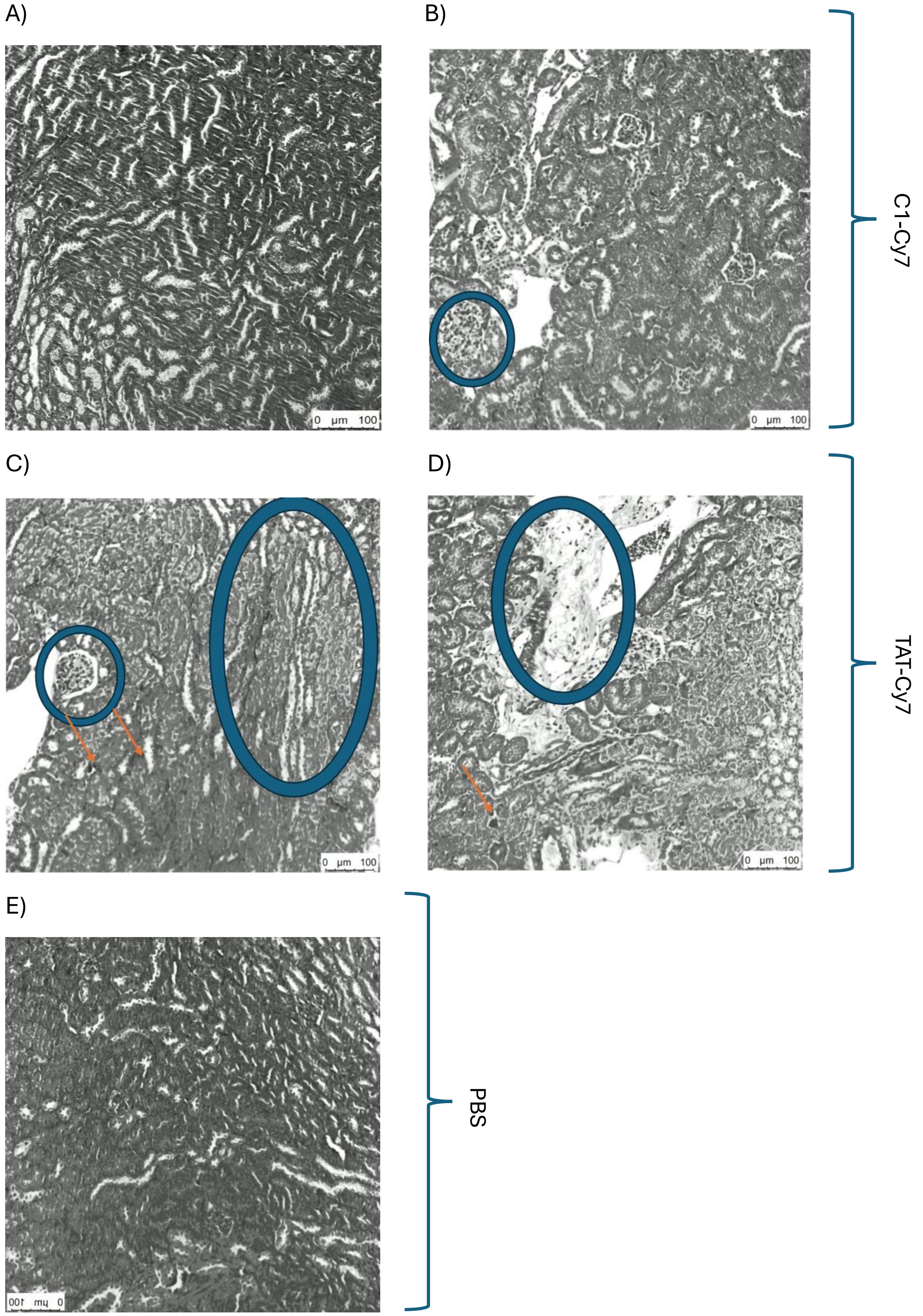

Figure S19. Light micrograph images of H&E stained kidney tissues from male mice treated with C1-Cy7 (A,B), TAT-Cy7 (C,D) and phosphate buffer saline PBS (E). Figs A-D: matrix deposition (dark areas, shown with orange arrows), and presence of fibrotic tissue and immune cells (in blue circles) detected to different extent. n=2 per study group.

Table S7. Kidney pathology scores for mice treatment groups, contrasted to healthy controls. n=2 per study group.

Study group/Pathology	Glomeruli	Tubules	Inflammation and fibrosis	Total score
F/C1-Cy7	0	0	0	0
F/TAT-Cy7	2	0	2	4
M/C1-Cy7	0.5	0	1	1.5
M/TAT-Cy7	1	1	2	4

F = female; M = male

Pathology of liver was assessed for hepatocytes by examining for ballooning degeneration, apoptosis, necrosis, and fatty changes (steatosis).[39,40]

Scoring was done as follows:

- 0: Normal hepatocytes.
- 1: Mild degeneration or steatosis (affecting <5% of hepatocytes).
- 2: Moderate changes (5-25%).
- 3: Severe changes (>25%).

We also revised for disruption in lobular architecture, such as fibrosis or bridging necrosis.

Scoring was done as follows:

- 0: Normal architecture.
- 1: Mild disruption or fibrosis.
- 2: Moderate disruption with fibrotic septa.
- 3: Severe architectural distortion with extensive fibrosis.

Only mild pathology is detected in males receiving TAT. All other groups have no changes in liver.

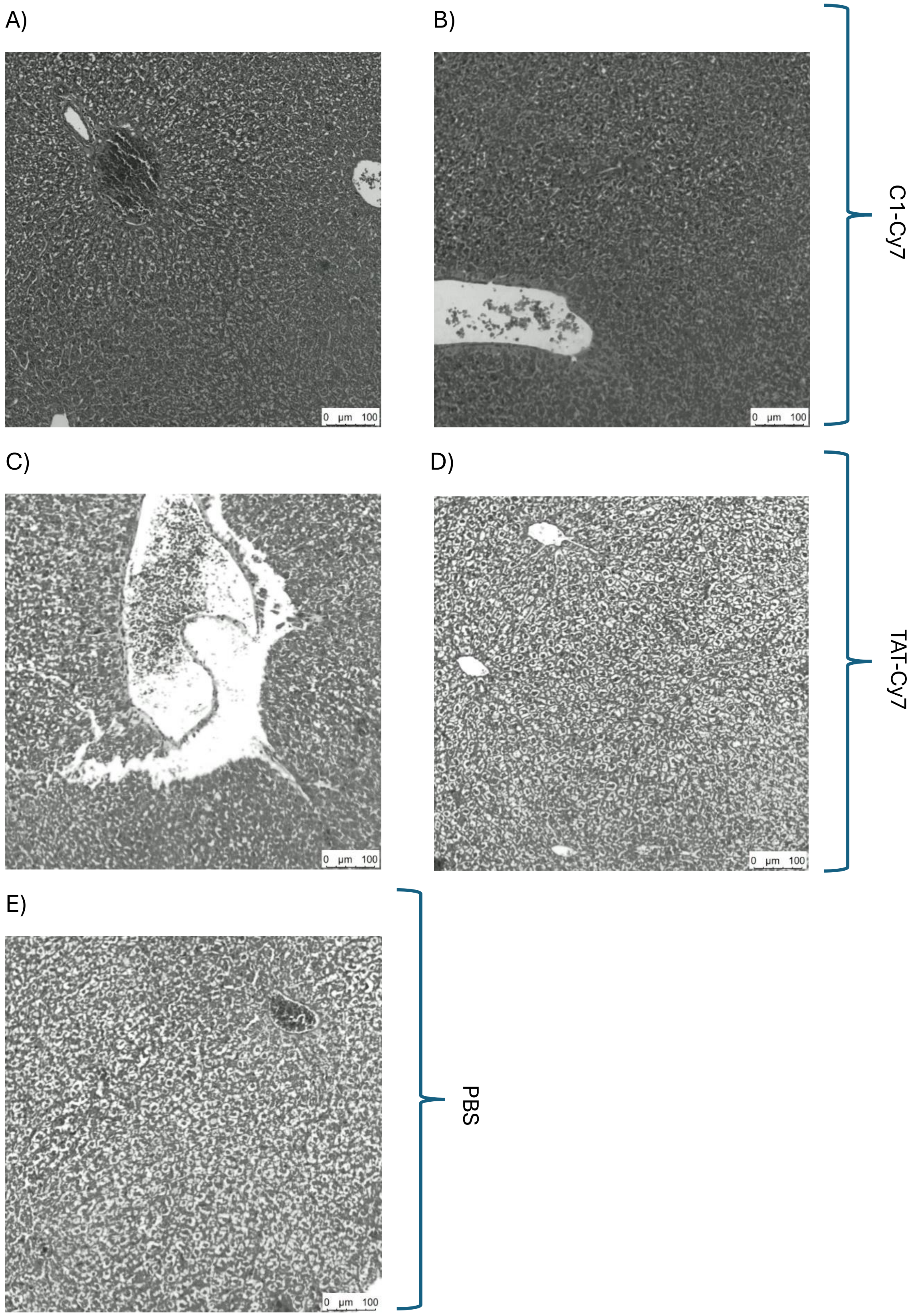

Figure S20. Light micrograph images of H&E stained liver tissues from female mice treated with C1-Cy7 (A,B), TAT-Cy7 (C,D) and phosphate buffer saline (PBS) (E). n=2 per study group.

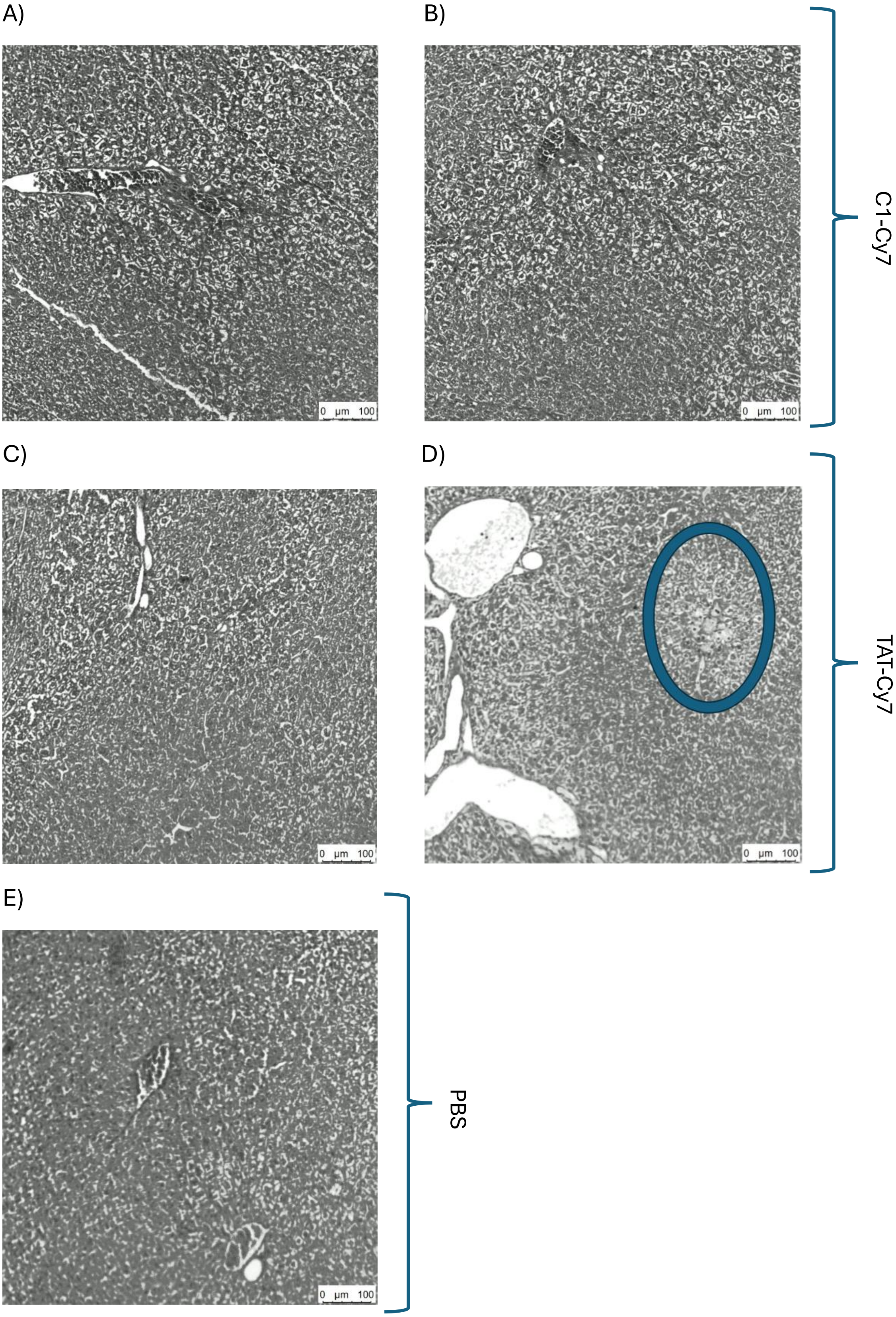

Figure S21. Light micrograph images of H&E stained liver tissues from male mice treated with C1-Cy7 (A,B), TAT-Cy7 (C,D) and phosphate buffer saline (PBS) (E). C-D) immune cell infiltration. n=2 per study group.

Table S8. Liver pathology scores for mice treatment groups, contrasted to healthy controls. N=2 per study group.

Study group/Pathology	Hepatocyte structure	Lobular fibrosis	Immune cell infiltration	Total score
F/C1-Cy7	0	0	0	0
F/TAT-Cy7	0	0	0	0
M/C1-Cy7	0	0	0	0
M/TAT-Cy7	0	0	1	1

F = female; M = male.

9.2 Pathology of lungs and spleen

Spleen pathology evaluation included assessment of following features: [41,42]

1. White Pulp:

Lymphoid hyperplasia/atrophy. Hyperplasia indicates an increase in the number of lymphocytes, while atrophy indicates a reduction.

Germinal Center Formation: Evaluate the presence and size of germinal centers within the white pulp, which indicates active immune responses.

2. Red Pulp:

- Hemosiderin Deposition: presence of hemosiderin, a breakdown product of hemoglobin, which appears as brown granules in macrophages. This may indicate increased red blood cell destruction or altered iron metabolism.

- Congestion: dilated blood vessels filled with erythrocytes, which may indicate circulatory disturbances or inflammation.

- Extramedullary Hematopoiesis (EMH): clusters of immature blood cells (megakaryocytes, myeloid cells) in the red pulp, indicating hematopoiesis outside the bone marrow.

3. Overall splenic architecture:

- Disruption of Architecture: changes in the normal organization of the spleen, which can indicate fibrosis, necrosis, or neoplastic infiltration.

Scoring was done as follows:

- 0: Normal

- 1: Mild changes (e.g., slight lymphoid hyperplasia, mild congestion)

- 2: Moderate changes (e.g., moderate hyperplasia, hemosiderin deposition, moderate congestion)

- 3: Severe changes (e.g., marked hyperplasia/atrophy, extensive EMH, severe congestion, fibrosis)

Minor pathology was detected in females received TAT (germinal centers and/or infiltrates in white pulp).

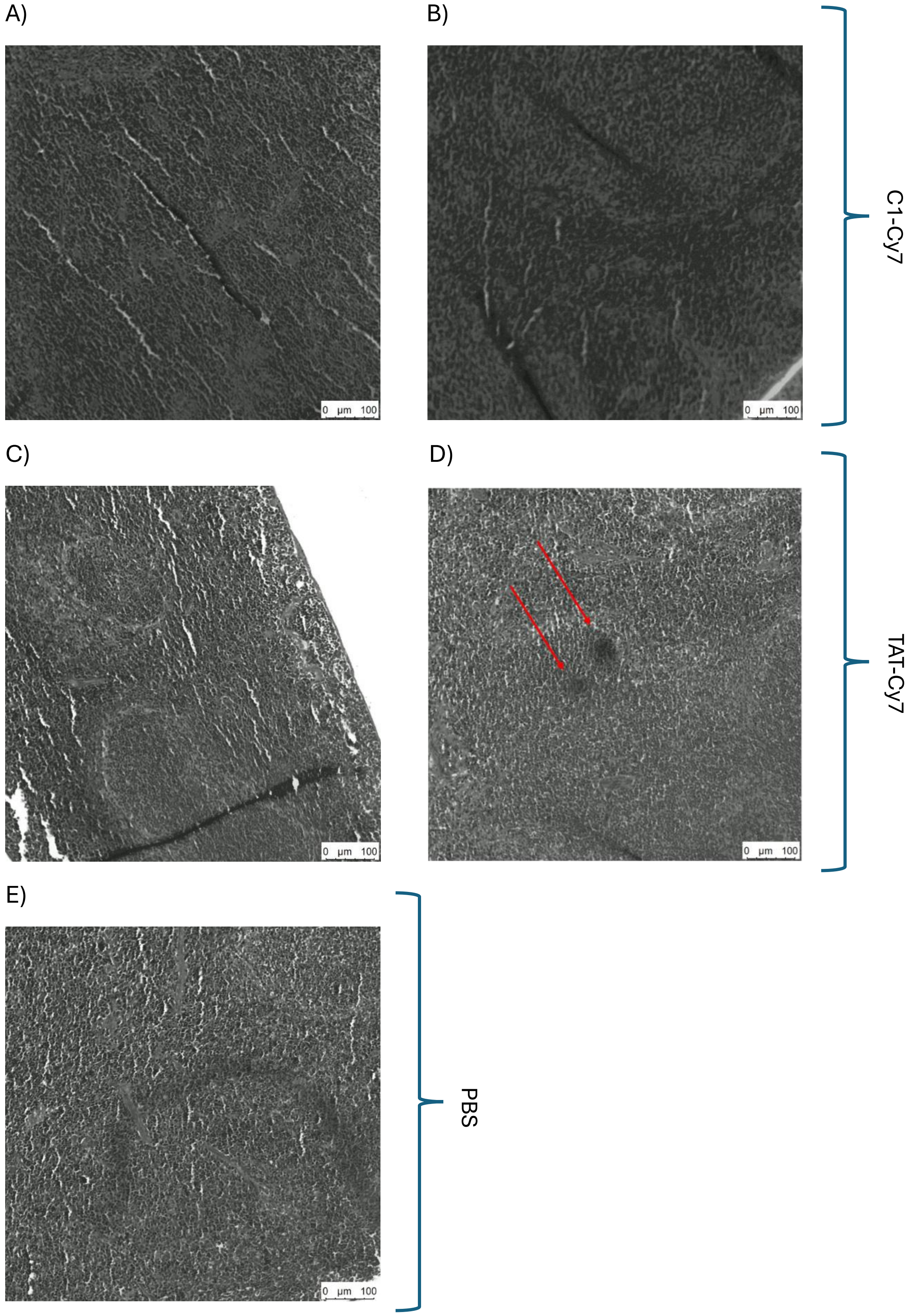

Figure S22. Light micrograph images of H&E stained spleen tissues from female mice treated with C1-Cy7 (A,B), TAT-Cy7 (C,D) and phosphate buffer saline (PBS) (E). Figure D, germinal center/infiltrates indicated in white pulp with red arrows. n=2 per study group.

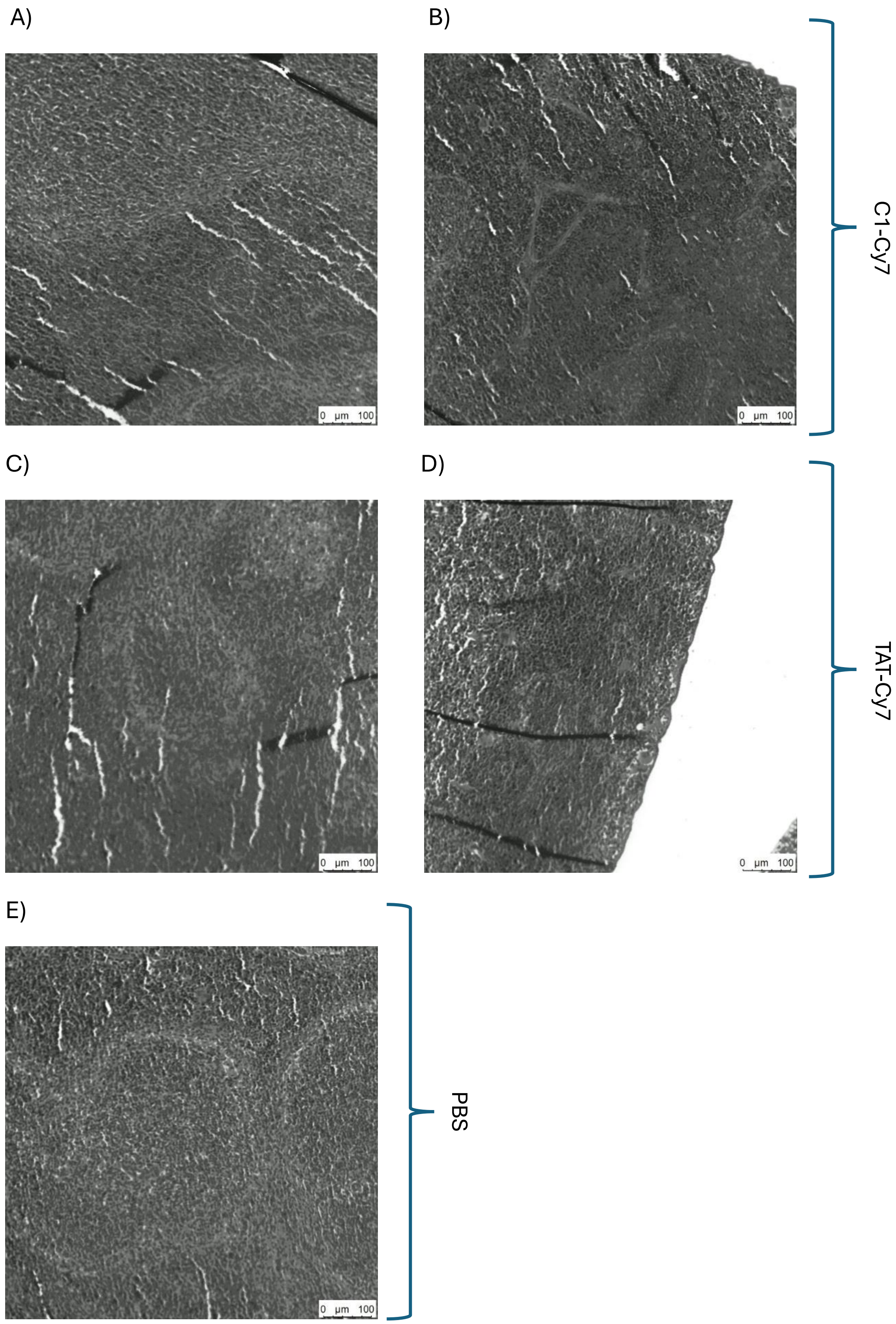

Figure S23. Light micrograph images of H&E stained spleen tissues from male mice treated with C1-Cy7 (A,B), TAT-Cy7 (C,D) and phosphate buffer saline (PBS) (E). n=2 per study group.

Table S9. Spleen pathology scores for mice treatment groups, contrasted to healthy controls. n=2 per study group.

Study group/Pathology	White pulp	Red pulp	Architecture	Total score
F/C1-Cy7	0	0	0	0
F/TAT-Cy7	1	0	0	1
M/C1-Cy7	0	0	0	0
M/TAT-Cy7	0	0	0	0

F = female; M = male.

Pathology of lungs was assessed for the following features:[43,44]

1. Alveoli:

- Emphysema: enlargement of alveolar spaces with destruction of alveolar walls.

- Edema: fluid accumulation within alveolar spaces.

- Inflammation: presence of immune cells (neutrophils, macrophages) within alveolar spaces, indicating conditions like pneumonia.

2. Bronchi and Bronchioles:

- Bronchitis/Bronchiolitis: inflammation of the bronchial and bronchiolar walls, characterized by thickening of the walls and infiltration of inflammatory cells.

- Hyperplasia: epithelial cell hyperplasia or metaplasia in the bronchi/bronchioles, which can indicate chronic irritation or infection.

3. Vascular Changes:

- Pulmonary Hypertension: thickening of the walls of pulmonary arteries, which can indicate increased blood pressure within the lung vasculature.

- Congestion: engorgement of blood vessels, indicating circulatory disturbances.

4. Interstitial Changes:

- Fibrosis: presence of collagen deposition in the interstitium, which thickens the alveolar septa and disrupts gas exchange.

- Inflammatory Infiltrate: immune cells within the interstitial spaces, which may indicate interstitial lung disease.

Scoring was done as follows:

- 0: Normal lung architecture with no significant changes.

- 1: Mild changes (e.g., mild alveolar inflammation, slight congestion).

- 2: Moderate changes (e.g., moderate bronchitis, interstitial inflammation, or fibrosis).

- 3: Severe changes (e.g., widespread emphysema, severe bronchitis/bronchiolitis, marked fibrosis).

Pathology of lungs was found in males and females, in a different fashion. Females had minor effect of C1-Cy7 on alveoli; TAT-Cy7 affected negatively alveoli and induced interstitial fibrosis.

In males, bronchi and vascular pathology were observed for C1-Cy7. TAT-Cy7 led to infiltration in lungs.

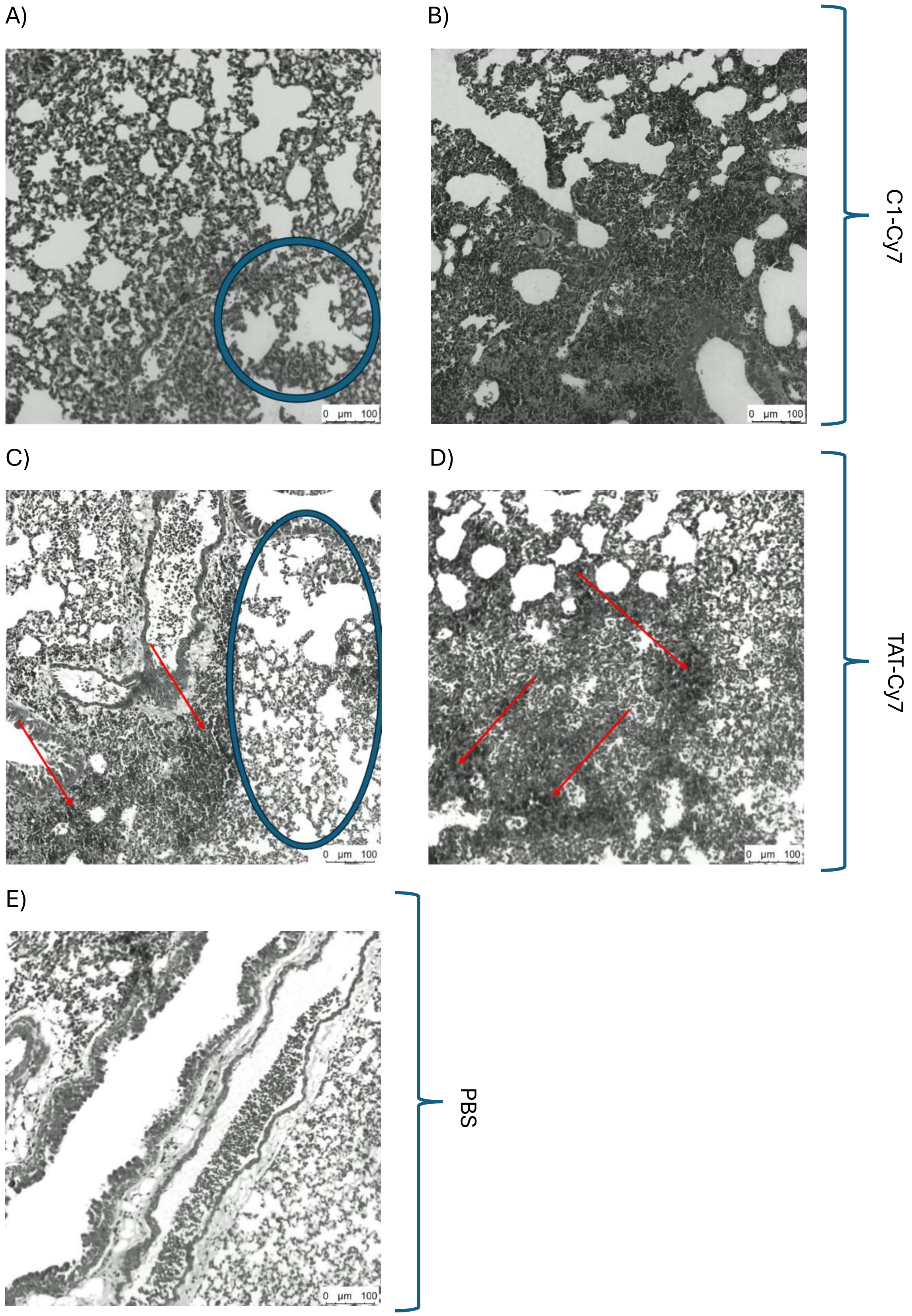

Figure S24. Light micrograph images of H&E stained lung tissues from female mice treated with C1-Cy7 (A,B), TAT-Cy7 (C,D) and phosphate buffer saline (PBS) (E). Alveoli

destruction (shown in blue circle) and fibrosis (shown with red arrows), indicated in Figures A,C,D. n=2 per study group.

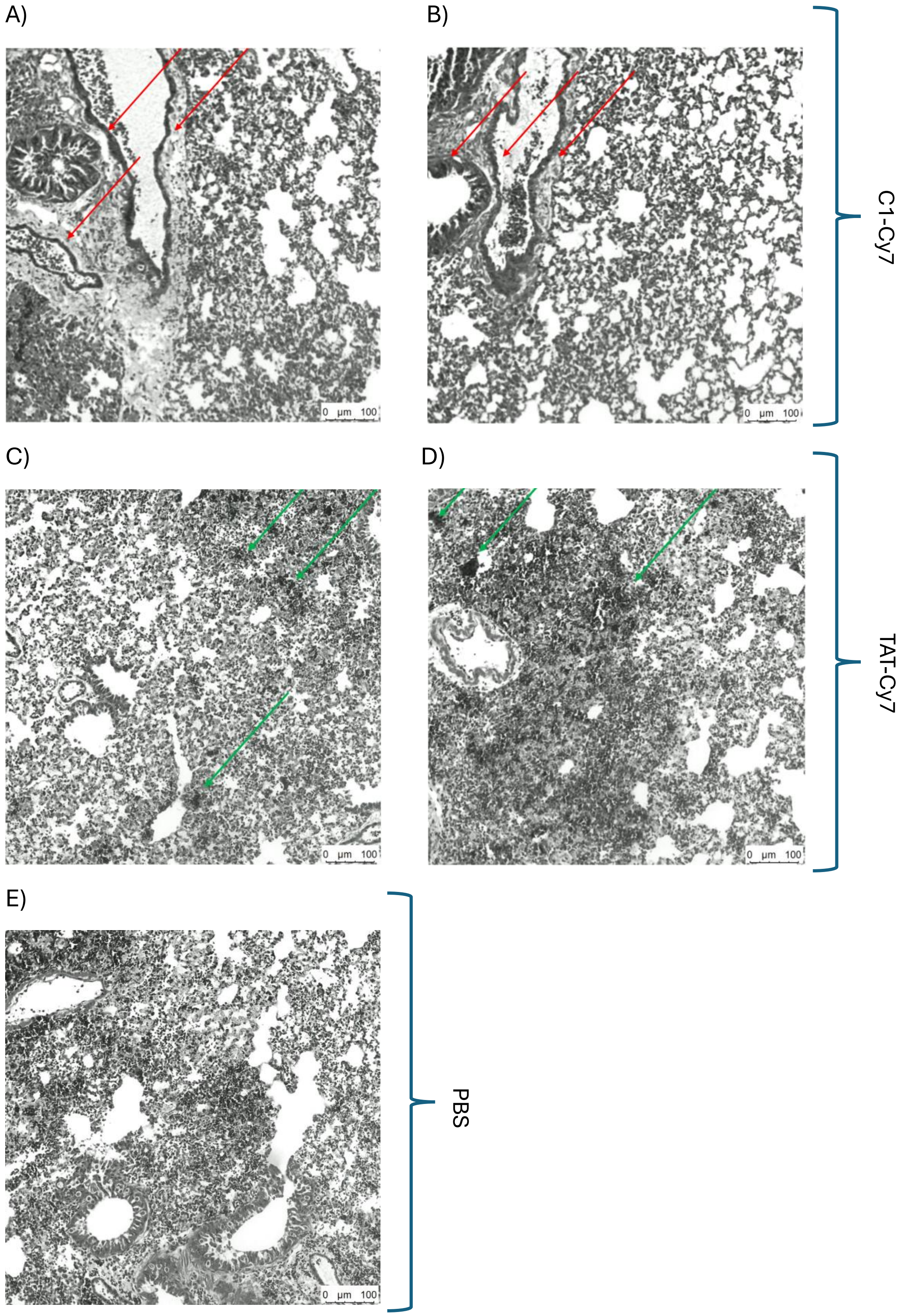

Figure S25. Light micrograph images of H&E stained lung tissues from male mice treated with C1-Cy7 (A,B), TAT-Cy7 (C,D) and phosphate buffer saline (PBS) (E). Wall thickening shown with red arrows; infiltrations shown with green arrows (A-D). n=2 per study group.

Table S10. Lung pathology scores for mice treatment groups, contrasted to healthy controls. n=2 per study group.

Study group/Pathology	Alveoli	Bronchi	Vascular	Interstitial	Total score
F/C1-Cy7	1	0	0	0	1
F/TAT-Cy7	2	0	0	3	5
M/C1-Cy7	0	1	1	0	2
M/TAT-Cy7	0	0	0	3	3

F = female; M = male.

9.3 Pathology assessment of brain

Brain tissue for each mouse was sectioned in three Ventral-Dorsal oriented levels:

-L1 mainly showing cortex;

-L2 mid Ventral level;

-L3 deep Ventral level.

H&E staining was performed followed by light micrographs obtained for three areas:

-Frontal lobes

-Midbrain

-Cerebellum

Levels and areas of brain sections used in this study are illustrated in Fig S26.[45-47] In total each mouse has had 9 brain sections analyzed.

A)

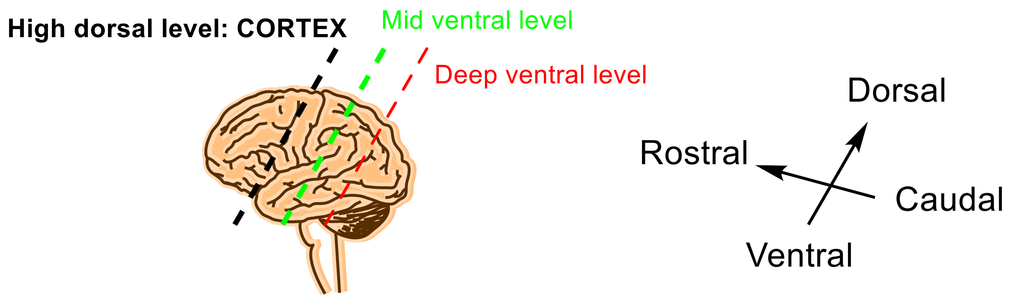

B)

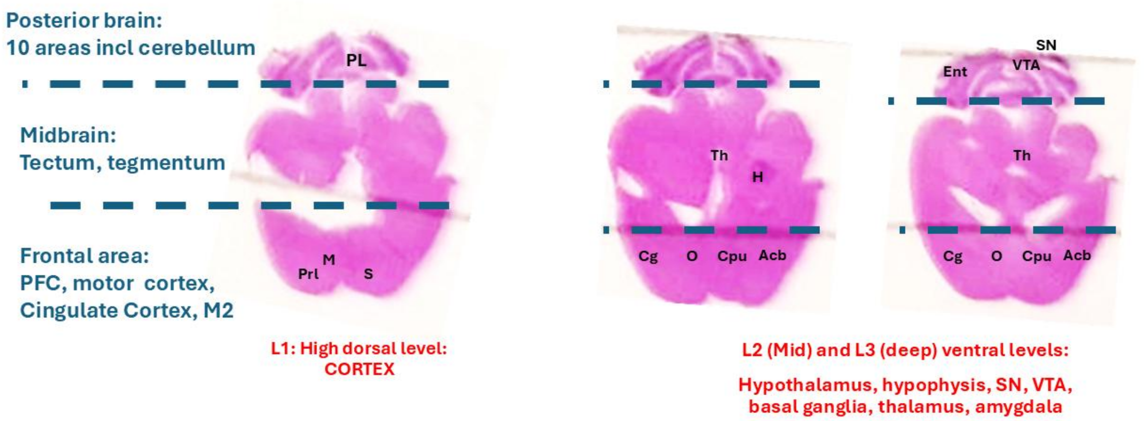

Figure S26. Levels and areas of the mouse brain analyzed in this study: A) Lateral view of brain areas analyzed; B) Analyzed mouse brain sections and levels with key areas indicated: Acb, nucleus accumbens; Cg, cingulate cortex; Cpu, caudate-putamen; Ent, entorhinal cortex; H, hypothalamus; M, motor cortex; O, orbital cortex; PL, posterior lobe; PrL, prelimbic cortex; S, somatosensory cortex; SN, substantia nigra; Th, thalamus; VTA, ventral tegmental area.

Analyzing and scoring pathology in H&E-stained mouse brain tissue involves identifying specific cell types in different brain regions and understanding how these regions are affected by disease or experimental conditions. Performed analyses and scoring approach are given below.

L1 Cortex

Pathology Analysis:

- Neuronal Density: number of neurons per unit area, noting any reductions that might indicate neurodegeneration.

- Cell Morphology: neuronal and glial morphology for signs of atrophy, swelling, or nuclear changes.

- Gliosis: the extent of reactive gliosis (increase in glial cells in response to injury).

Mid Ventral Level (L2)

Pathology Analysis:

- Neuronal Loss: neuronal dropout, particularly in key nuclei like the thalamus.

- Vacuolization: vacuoles in the neuropil, which might indicate neurodegeneration or metabolic issues.

- Gliosis: increased glial cell proliferation, particularly around areas of damage.

Deep Ventral Level (L3)

Pathology Analysis:

- Neuronal Degeneration: motor neurons in areas like the brainstem and basal ganglia.

- Axonal Damage: signs of axonal degeneration or demyelination.

- Inflammatory Infiltrates: presence of immune cells indicative of neuroinflammation.

In addition, in frontal lobes white matter myelination patterns and axonal integrity was analyzed. Focus was paid to the severity of atrophy, gliosis, and any cortical thinning.

In midbrain, infiltration of immune cells has been accounted for.

In cerebellum, density and morphology of Purkinje cells was assessed.

Applied scale for all pathology types has been as follows:

0: Normal, no pathology observed.

- 1: Mild changes, minimal impact on tissue architecture; number of neurons

- 2: Moderate changes, noticeable impact on cell density or structure

- 3: Severe changes, significant disruption of tissue structure.

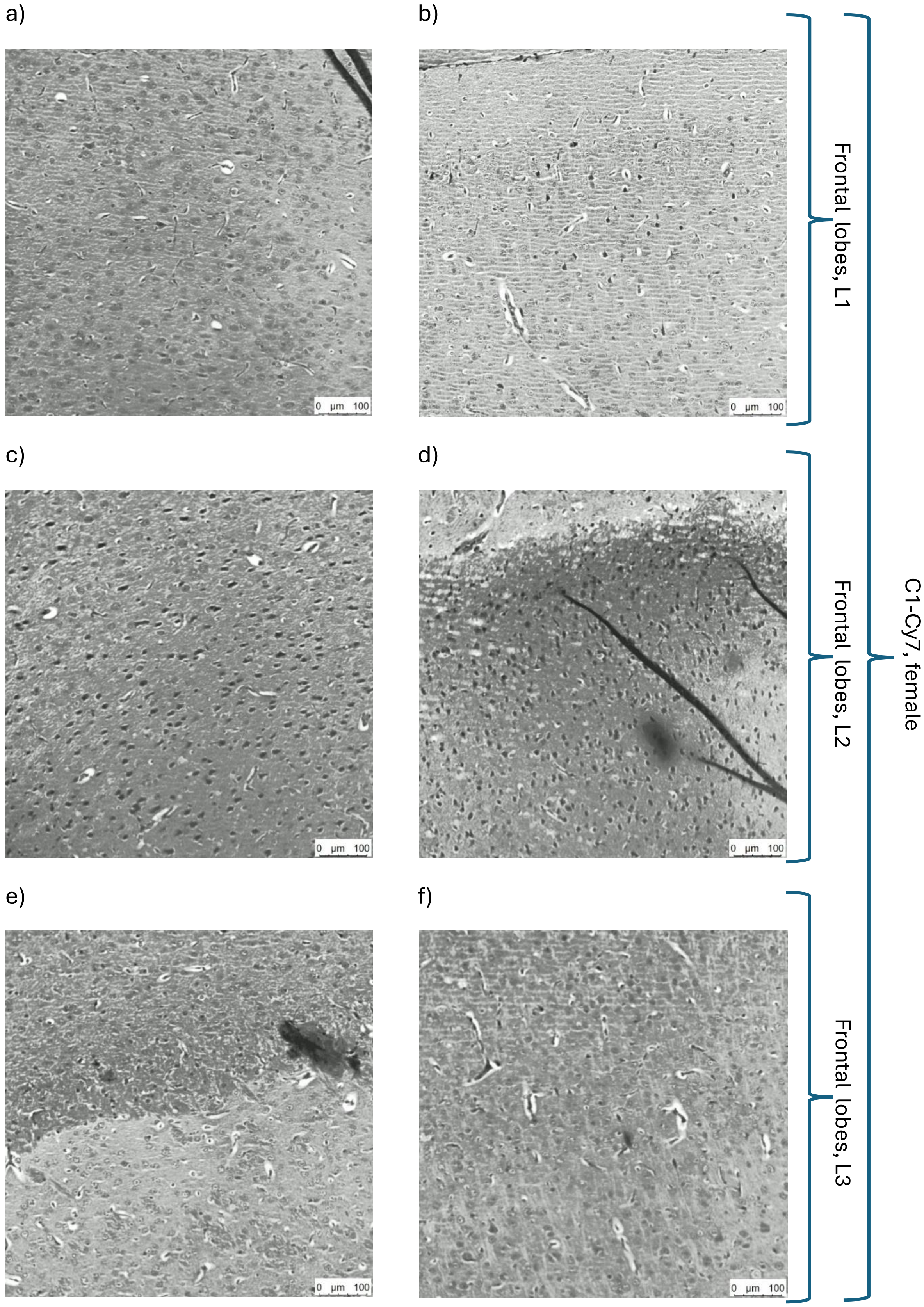
a)
b)
c)
d)
e)
f)
0 µm 100
Frontal lobes, L1
Frontal lobes, L2
Frontal lobes, L3
C1-Cy7, female

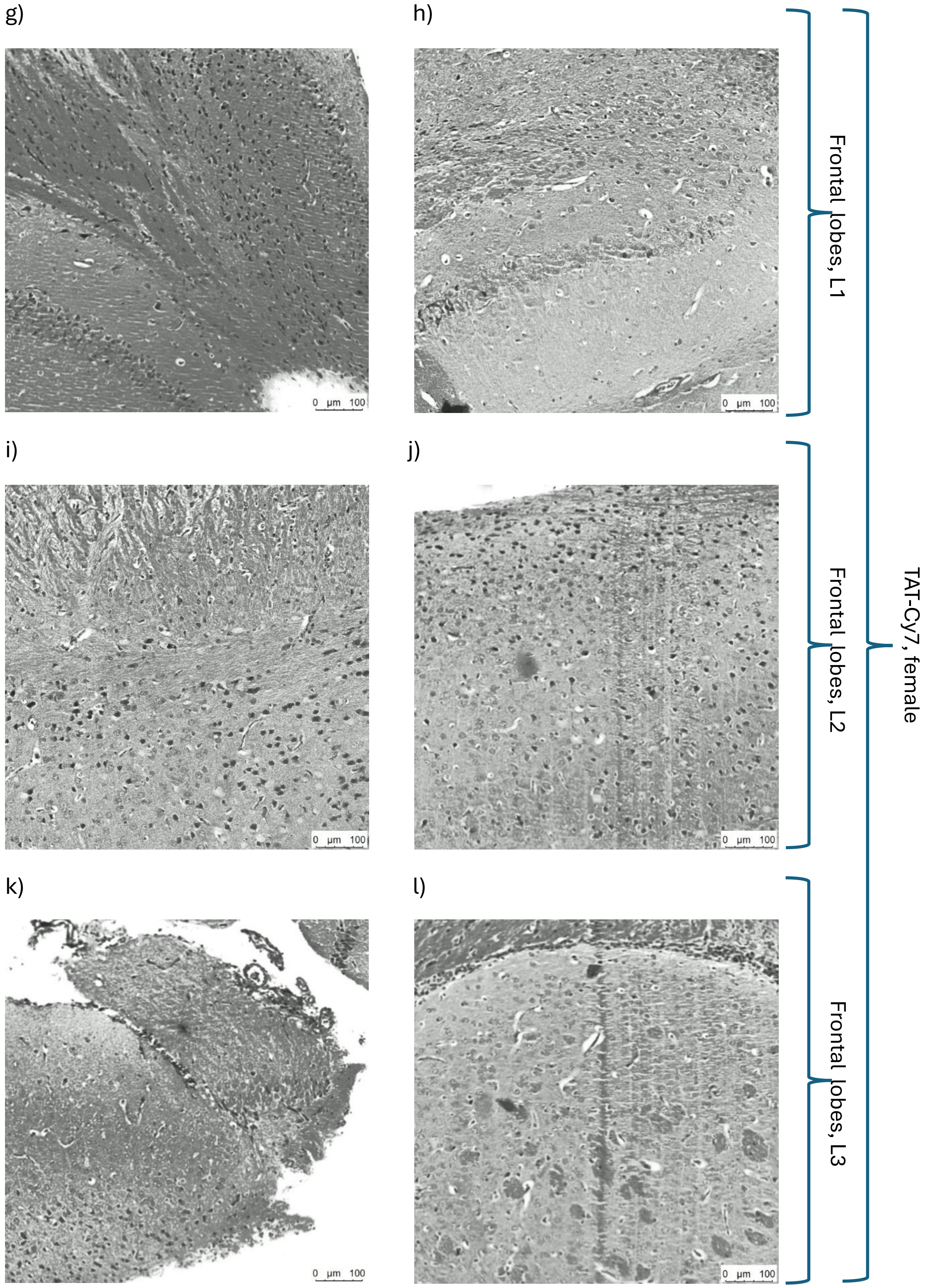
g)
h)
i)
j)
k)
l)
Frontal lobes, L1
Frontal lobes, L2
Frontal lobes, L3
TAT-Cy7, female

m) Frontal lobes, L1

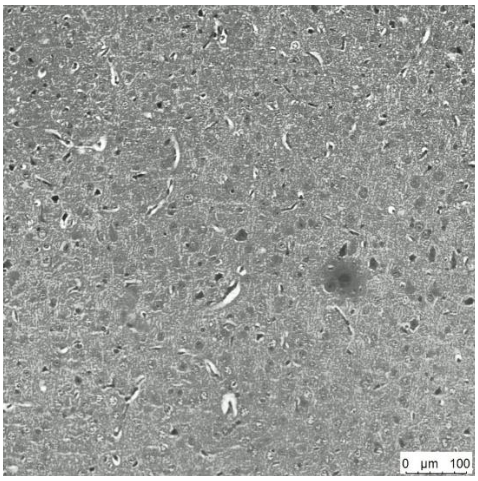

n) Frontal lobes, L2

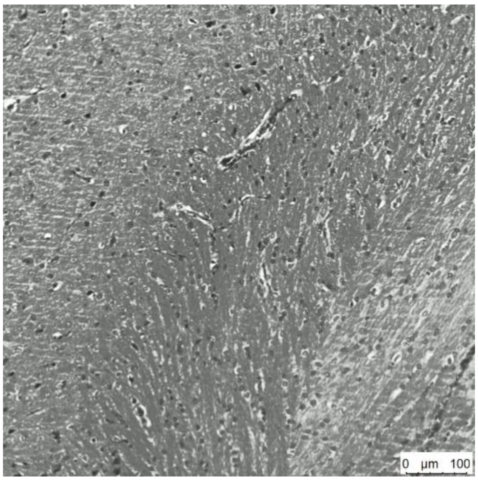

o) Frontal lobes, L3

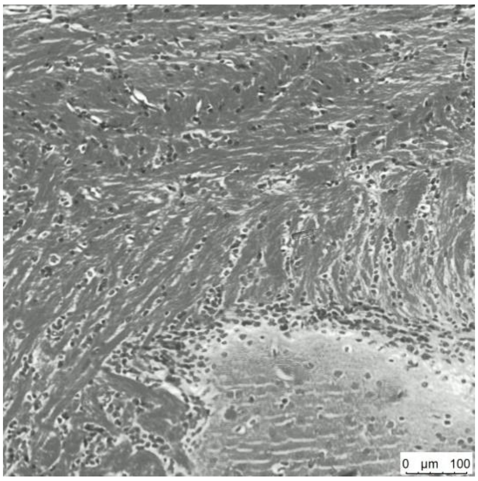

PBS, female

Figure S27. Light micrograph images of H&E stained brain tissues from female mice treated with C1-Cy7, TAT-Cy7 and phosphate buffer saline (PBS) (control); frontal lobes; C1-Cy7, L1: a),b); L2, c),d); L3, e),f); TAT-Cy7, L1, g),h); L2,i),j); L3, k), l); PBS, L1, m); L2, n); L3, o). n=2 per study group.

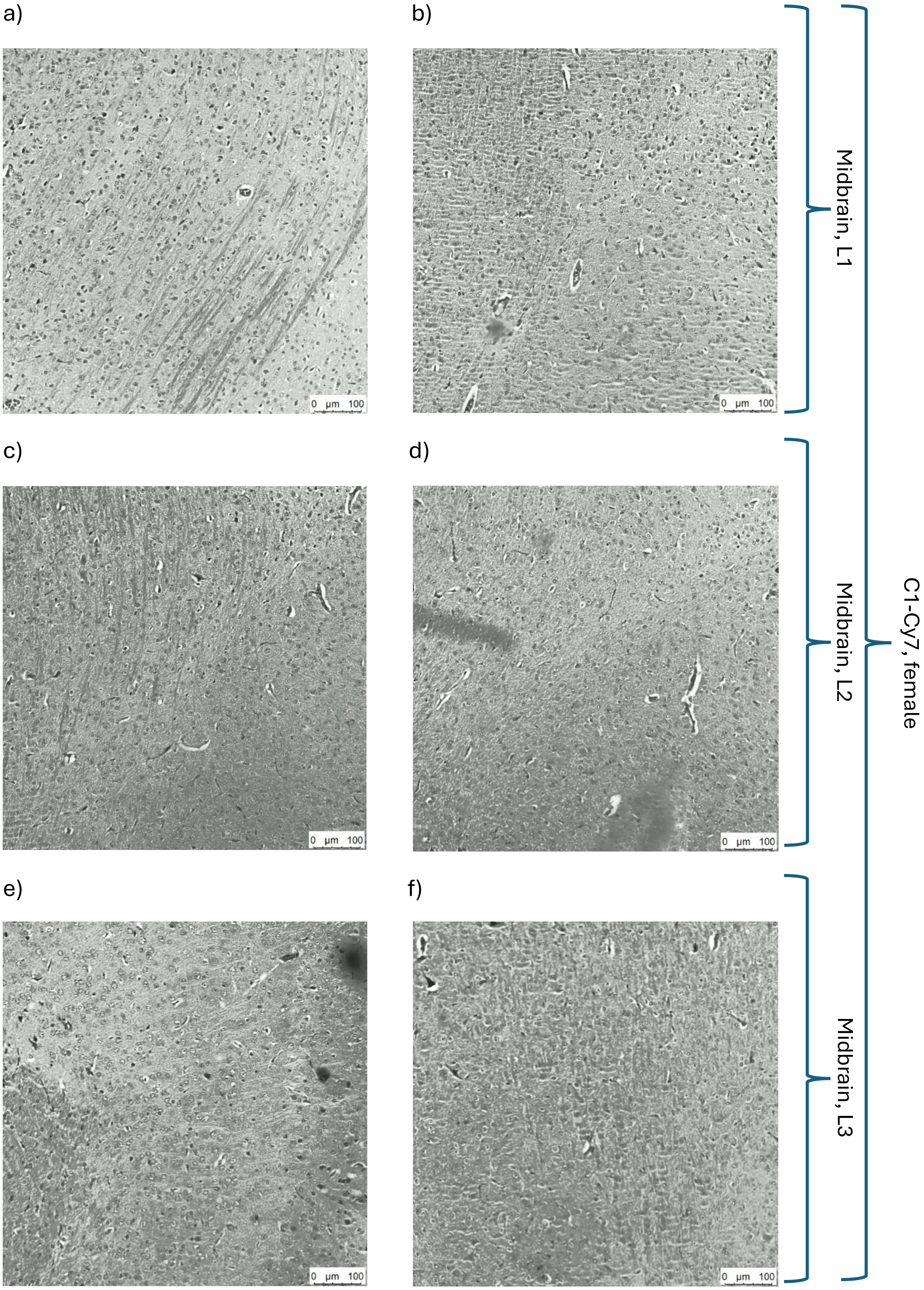
a)
b)
c)
d)
e)
f)
0 µm 100
Midbrain, L1
Midbrain, L2
Midbrain, L3
C1-Cy7, female

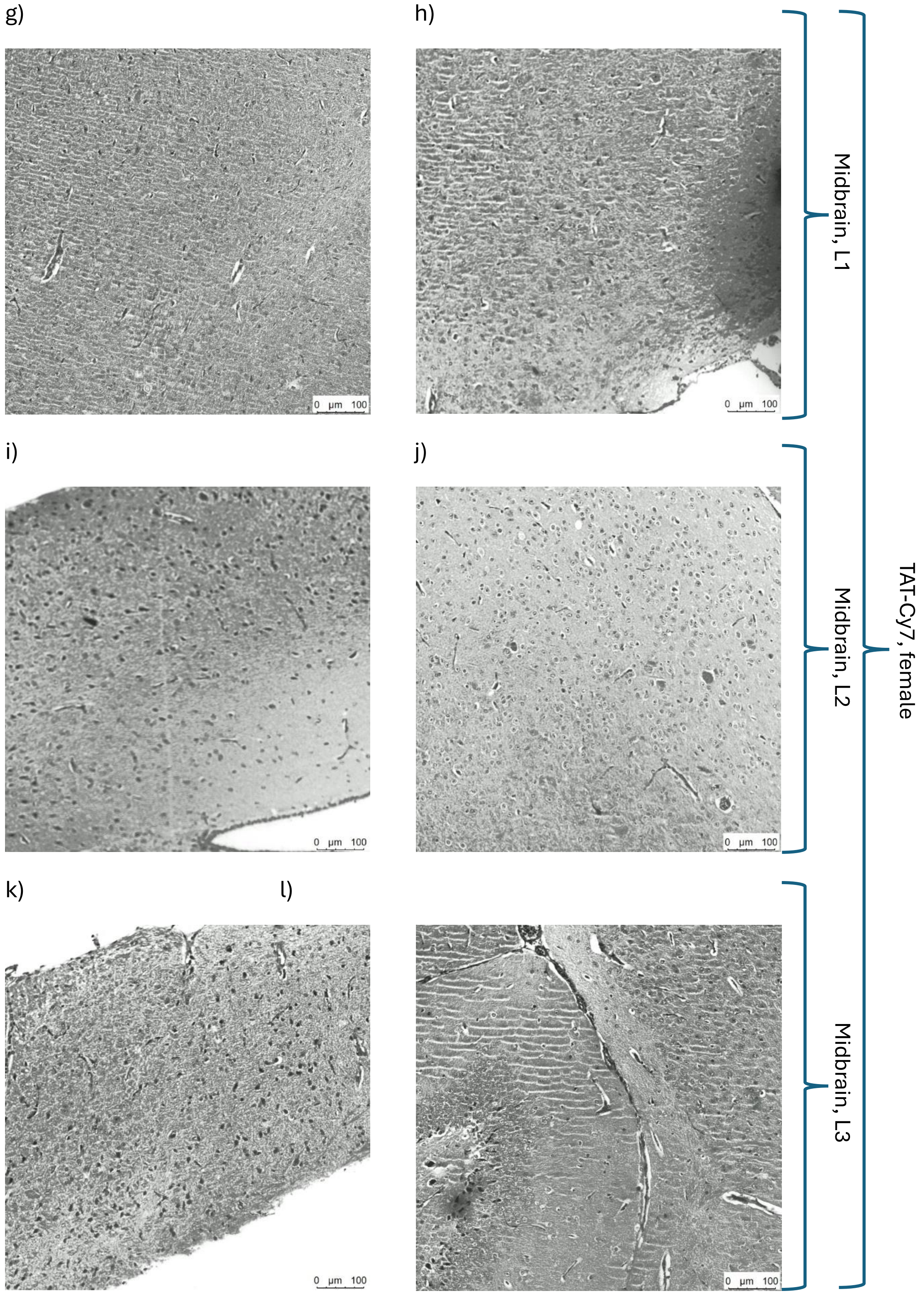
g)
h)
0 µm 100
Midbrain, L1
i)
j)
Midbrain, L2
k)
l)
Midbrain, L3
TAT-Cy7, female

m) Midbrain, L1

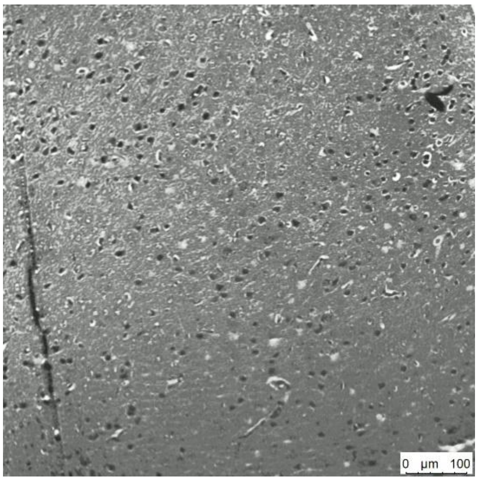

n) Midbrain, L2

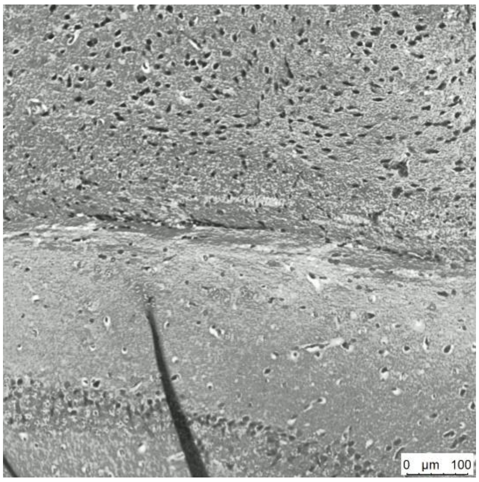

o) Midbrain, L3

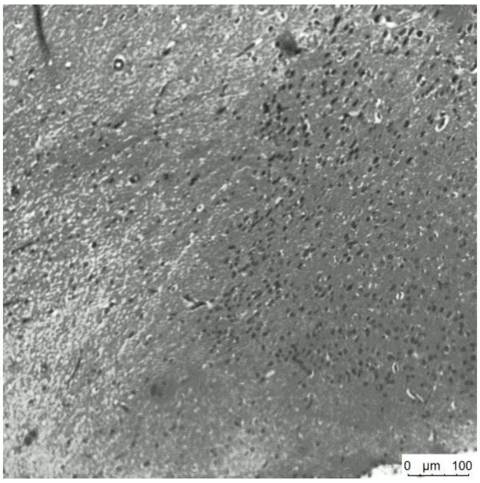

Figure S28. Light micrograph images of H&E stained brain tissues from female mice treated with C1-Cy7, TAT-Cy7 and phosphate buffer saline (PBS) (control); midbrain; C1-Cy7, L1: a),b); L2, c),d); L3, e),f); TAT-Cy7, L1, g),h); L2,i),j); L3, k), l); PBS, L1, m); L2, n); L3, o). n=2 per study group.

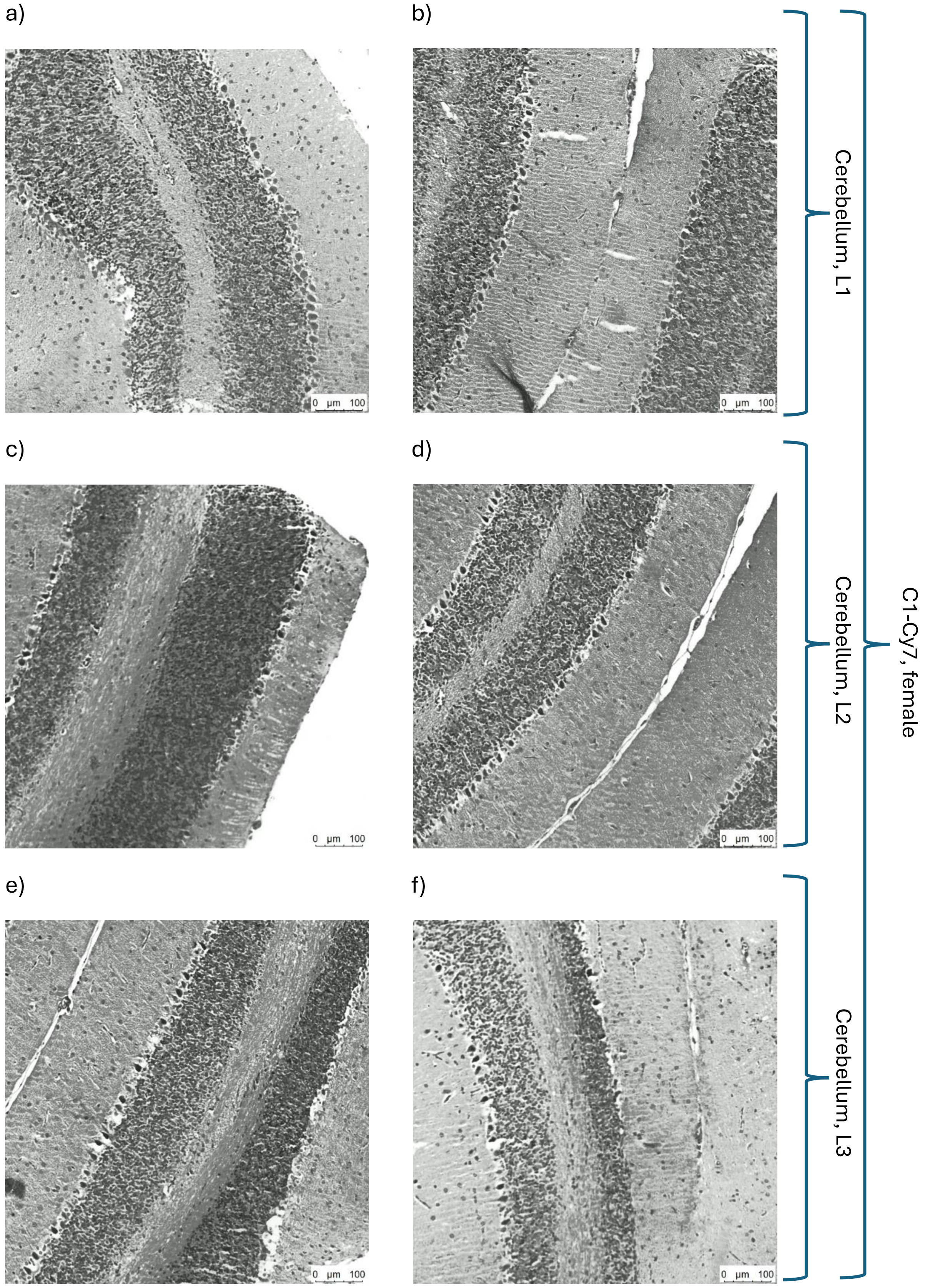
a)
b)
c)
d)
e)
f)
0 µm 100
Cerebellum, L1
Cerebellum, L2
Cerebellum, L3
C1-Cy7, female

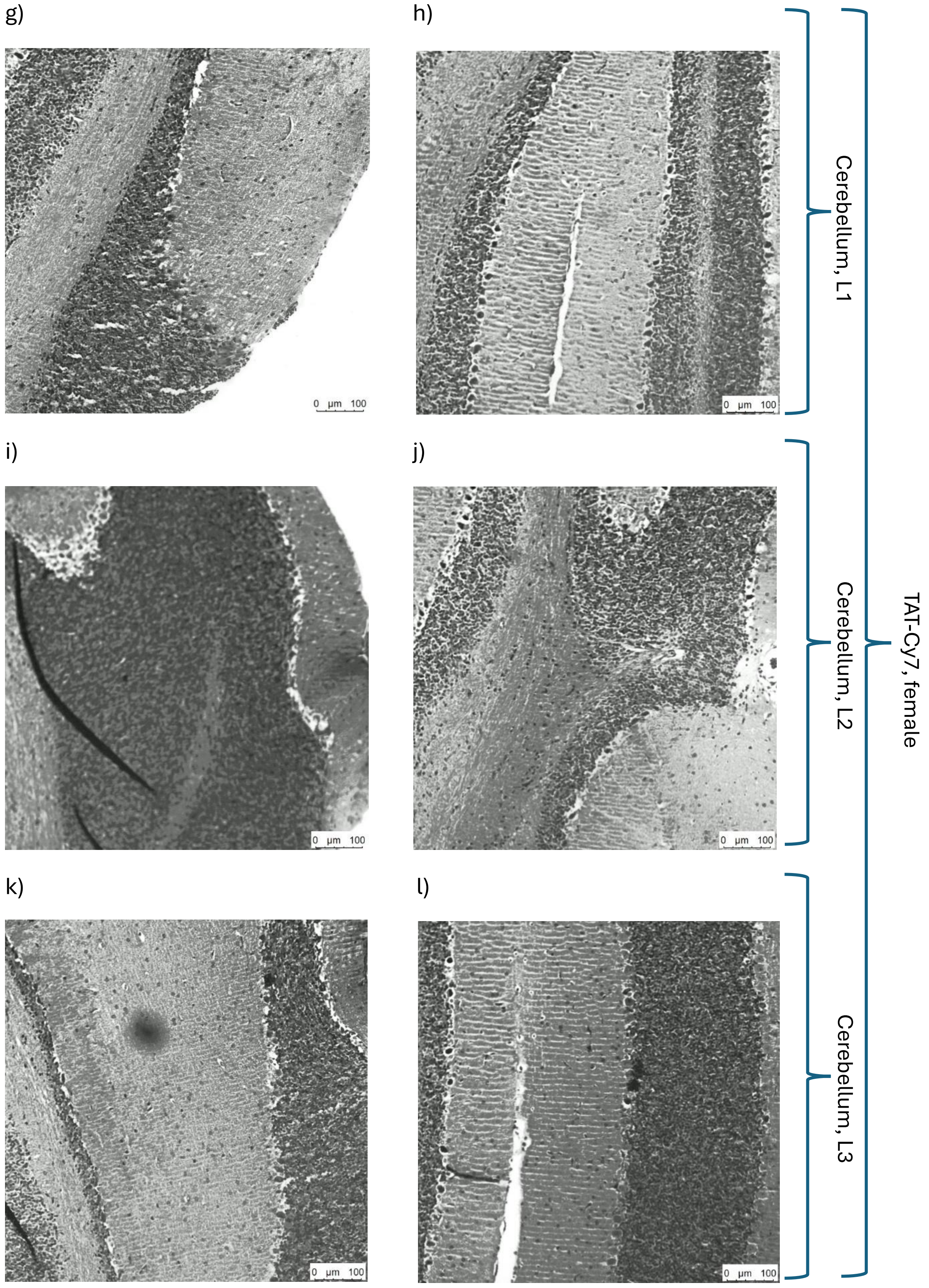
g)
h)
i)
j)
k)
l)
0 µm 100
Cerebellum, L1
Cerebellum, L2
Cerebellum, L3
TAT-Cy7, female

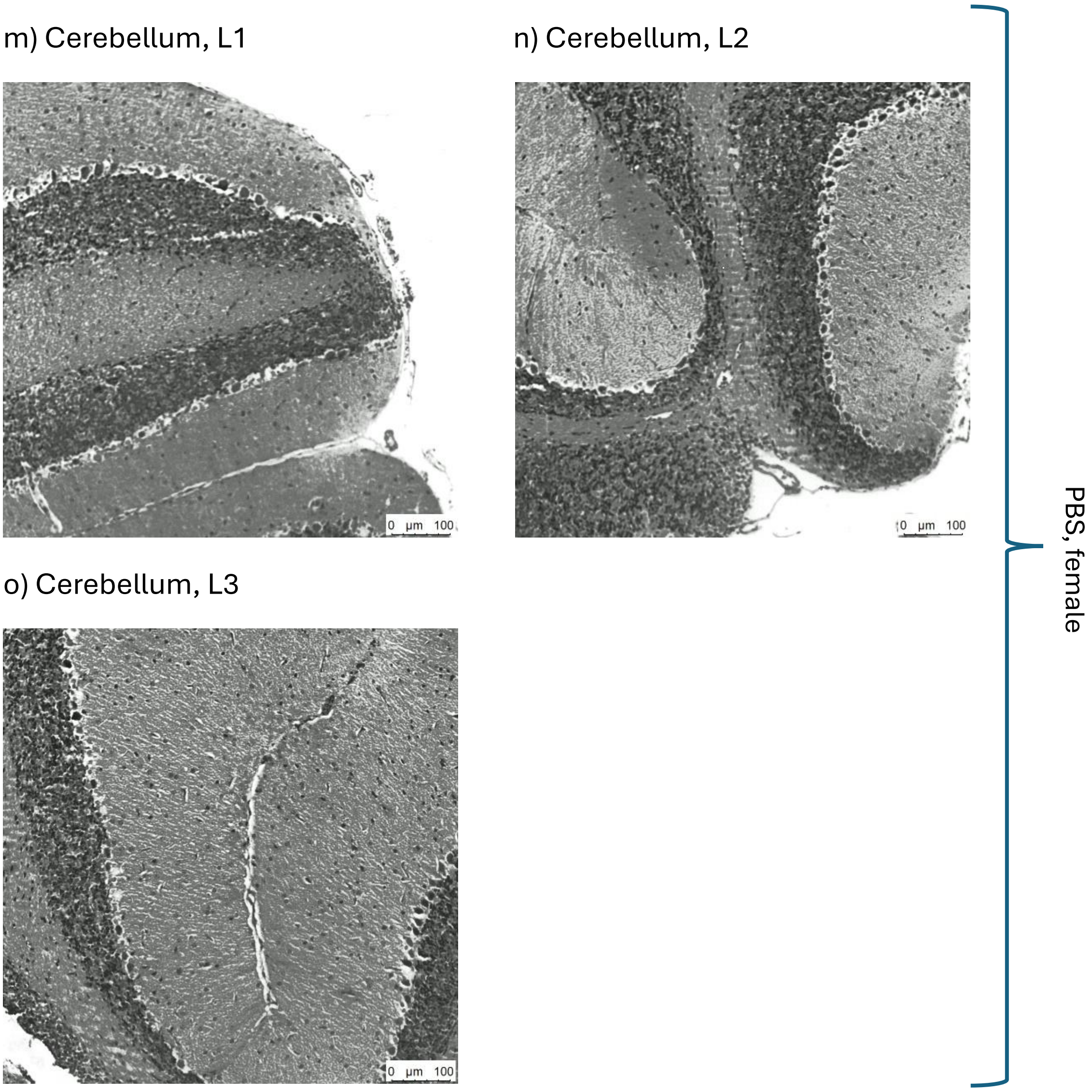

Figure S29. Light micrograph images of H&E stained brain tissues from female mice treated with C1-Cy7, TAT-Cy7 and PBS (control); cerebellum; C1-Cy7, L1: a),b); L2, c),d); L3, e),f); TAT-Cy7, L1, g),h); L2,i),j); L3, k), l); PBS, L1, m); L2, n); L3, o). n=2 per study group.

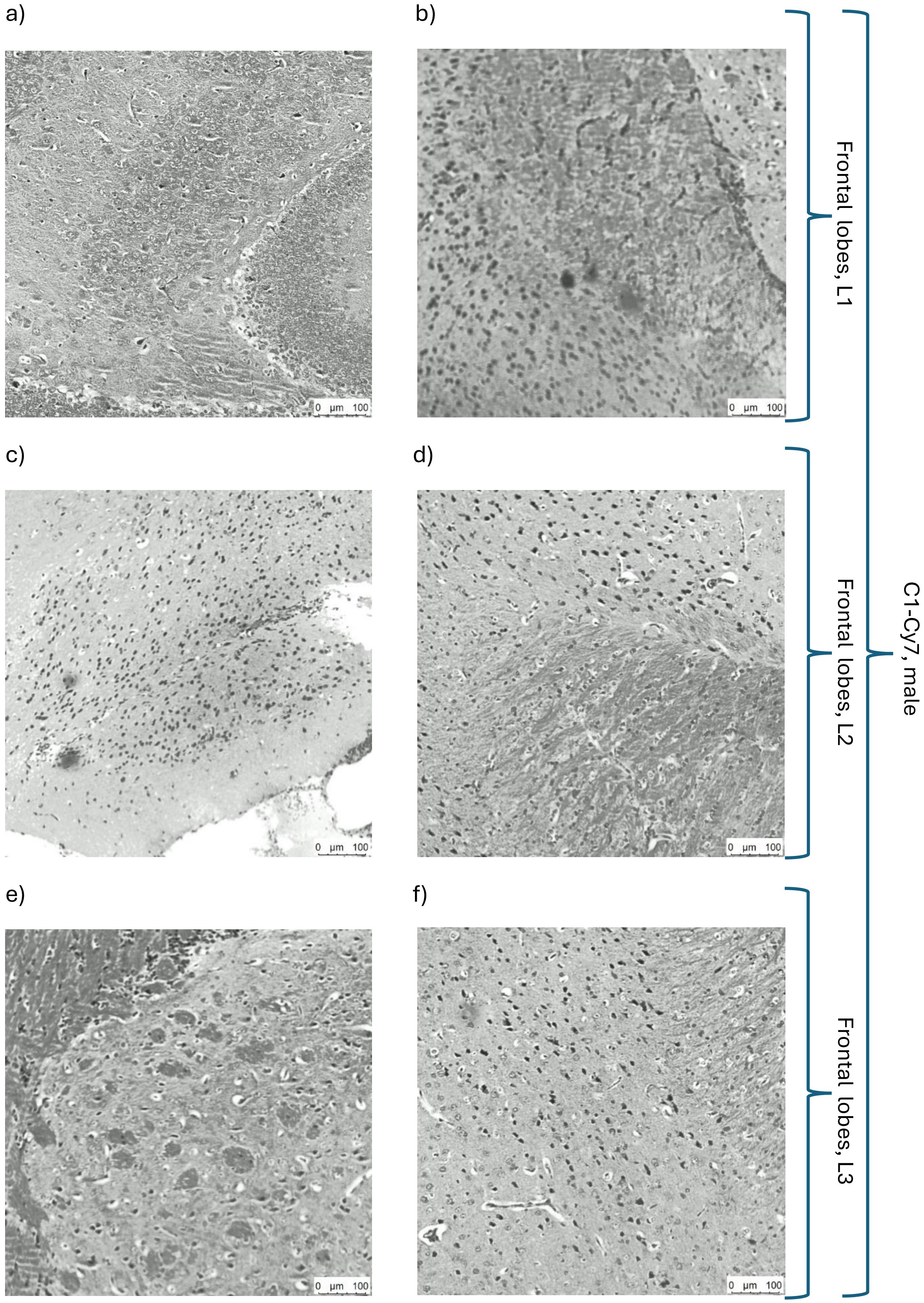
a)
b)
c)
d)
e)
f)
0 µm 100
Frontal lobes, L1
Frontal lobes, L2
Frontal lobes, L3
C1-Cy7, male

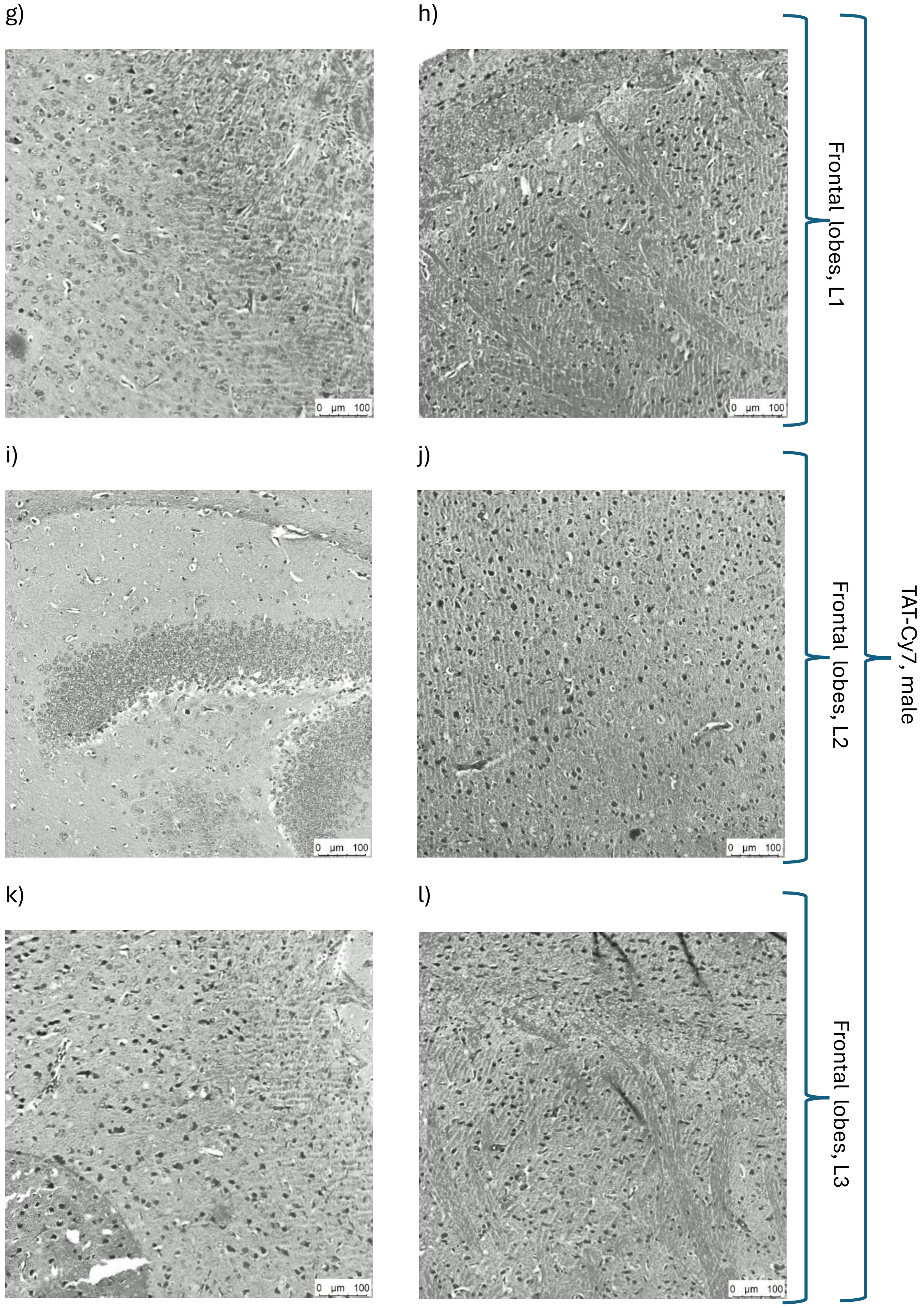
g)
h)
i)
j)
k)
l)
0 µm 100
Frontal lobes, L1
Frontal lobes, L2
Frontal lobes, L3
TAT-Cy7, male

m) Frontal lobes, L1

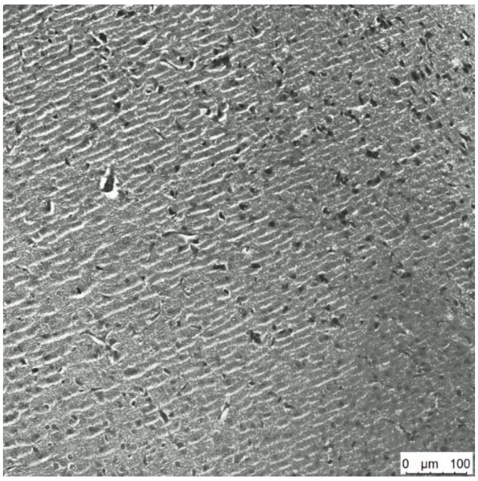

n) Frontal lobes, L2

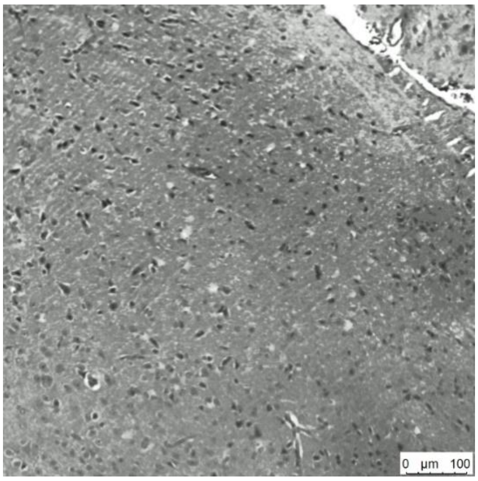

o) Frontal lobes, L3

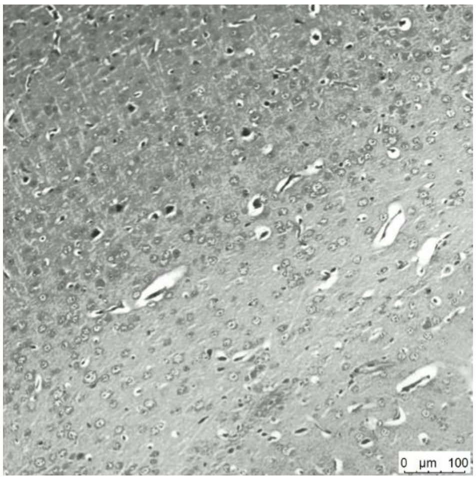

PBS, male

Figure S30. Light micrograph images of H&E stained brain tissues from male mice treated with C1-Cy7, TAT-Cy7 and phosphate buffer saline (PBS) (control); frontal lobes; C1-Cy7, L1: a),b); L2, c),d); L3, e),f); TAT-Cy7, L1, g),h); L2,i),j); L3, k), l); PBS, L1, m); L2, n); L3, o). n=2 per study group.

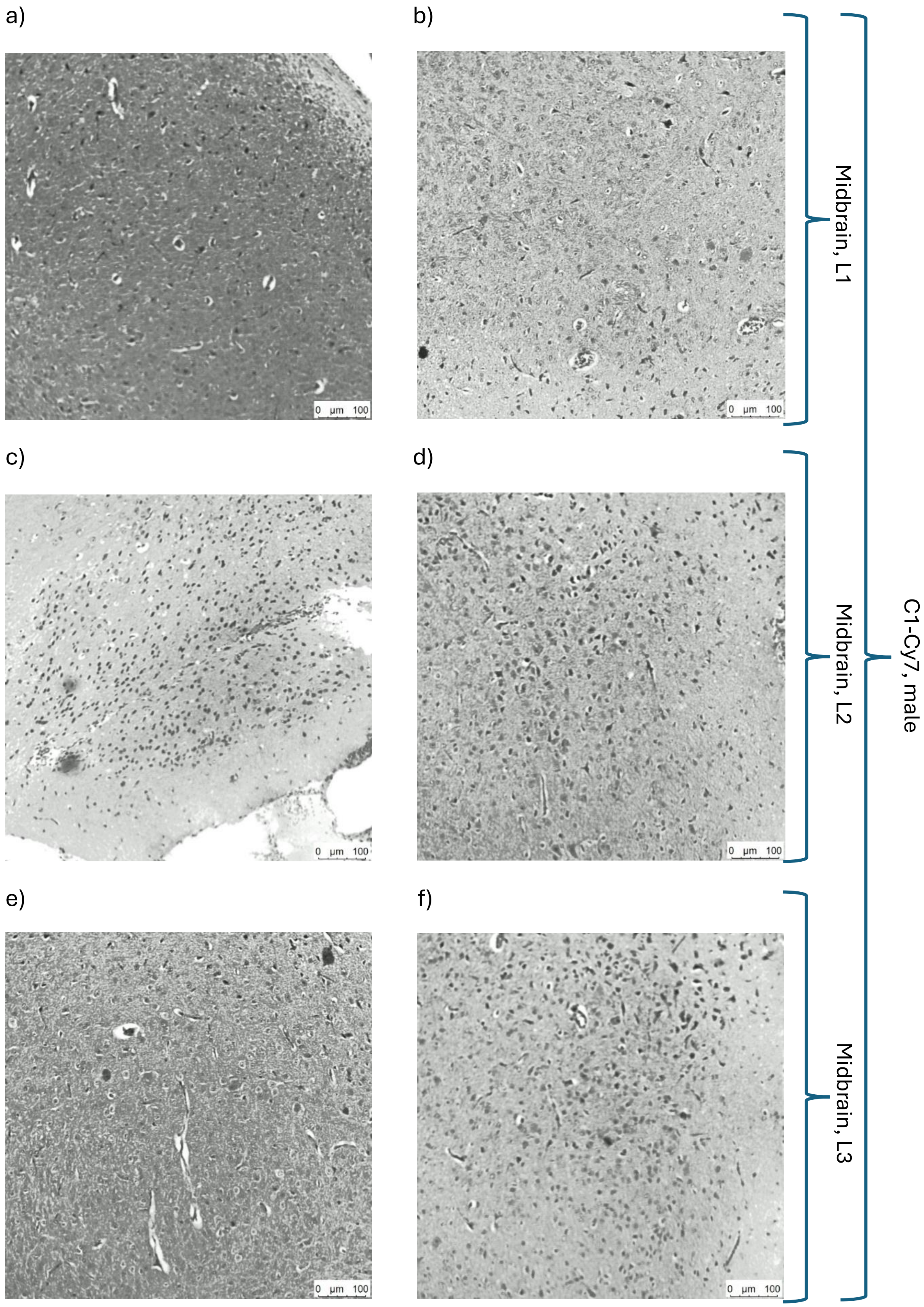
a)
b)
c)
d)
e)
f)
0 µm 100
Midbrain, L1
Midbrain, L2
Midbrain, L3
C1-Cy7, male

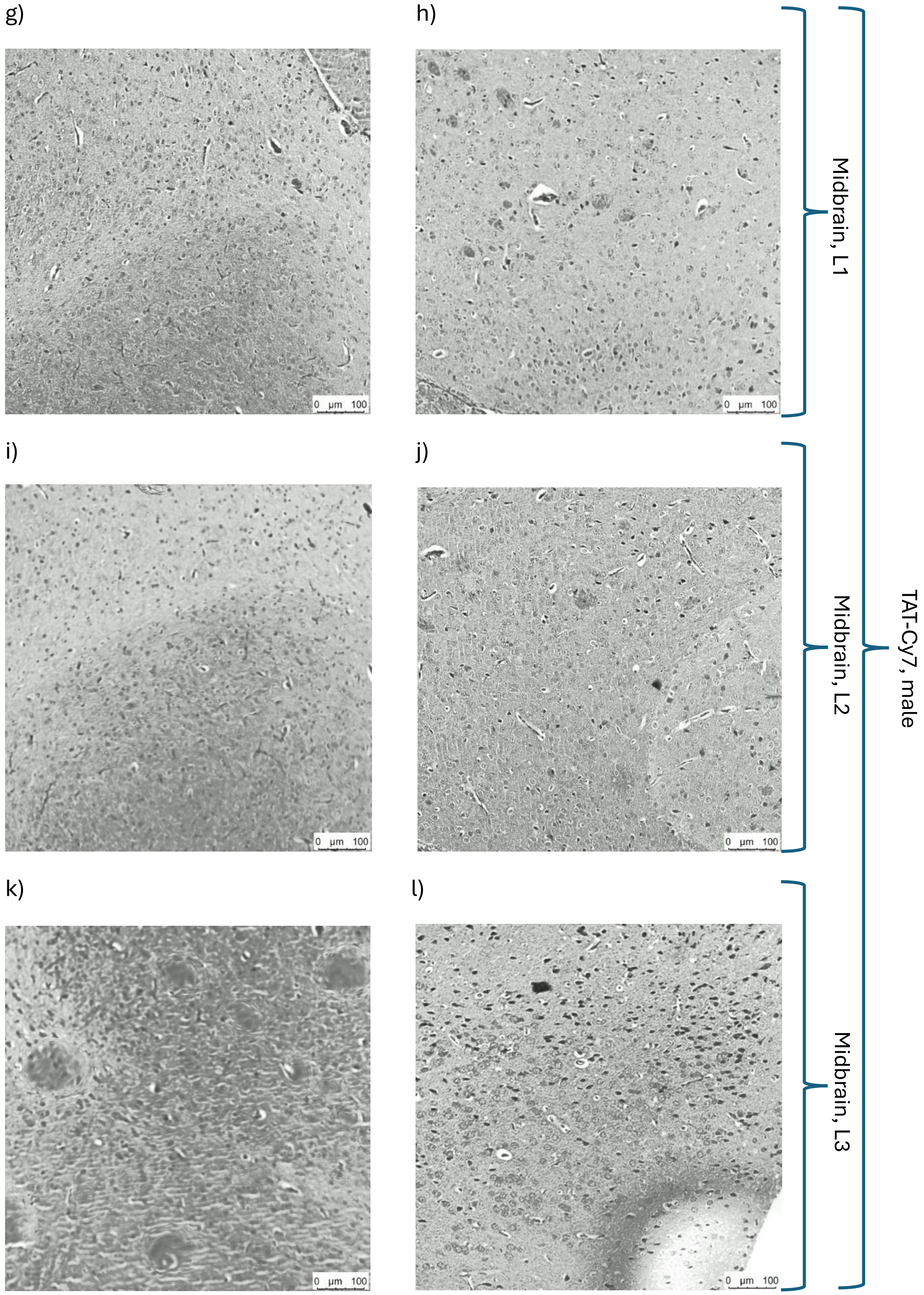
g)
h)
i)
j)
k)
l)
0 µm 100
Midbrain, L1
Midbrain, L2
Midbrain, L3
TAT-Cy7, male

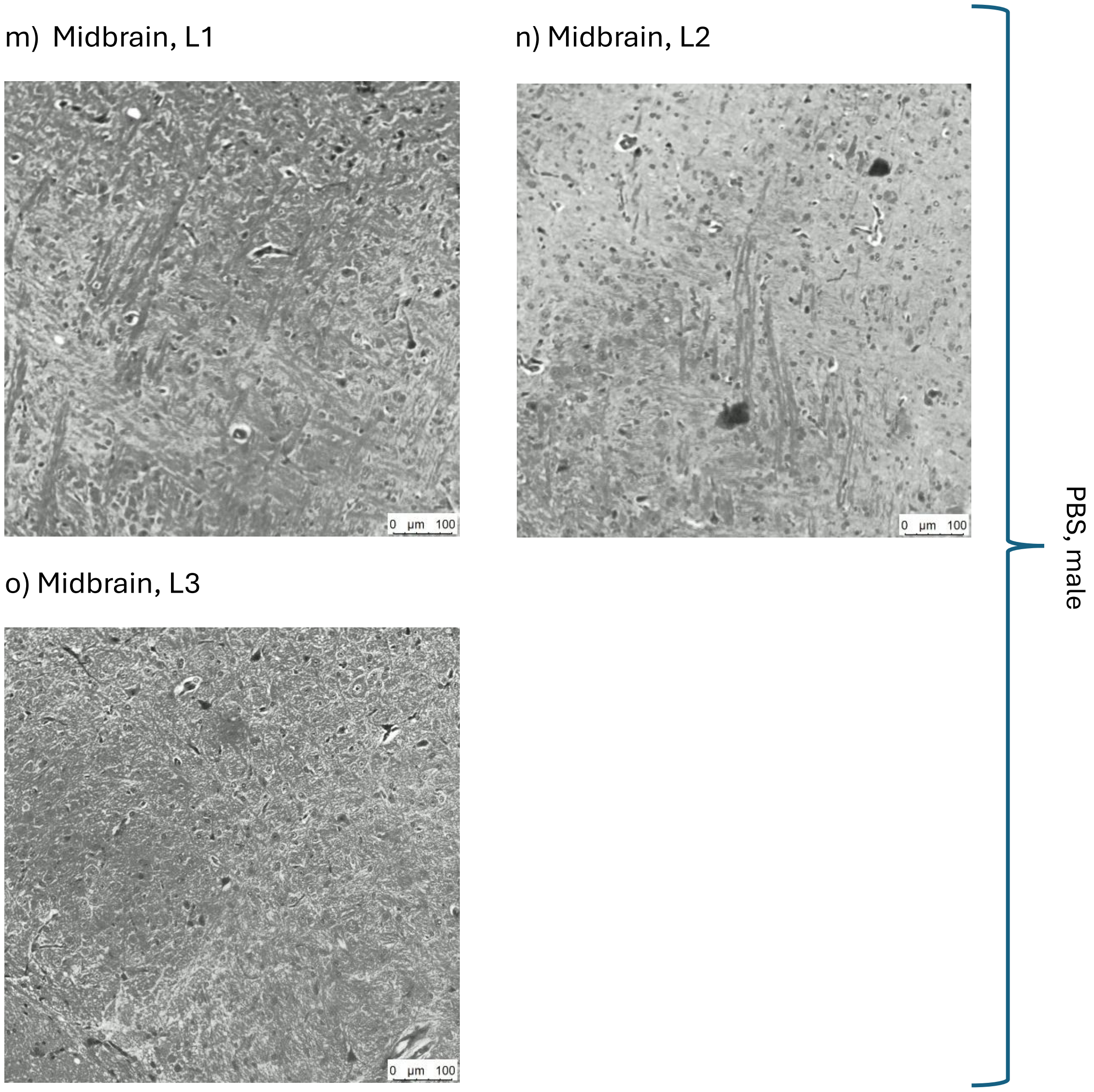

Figure S31. Light micrograph images of H&E stained brain tissues from male mice treated with C1-Cy7, TAT-Cy7 and phosphate buffer saline (PBS) (control); midbrain; C1-Cy7, L1: a),b); L2, c),d); L3, e),f); TAT-Cy7, L1, g),h); L2,i),j); L3, k), l); PBS, L1, m); L2, n); L3, o). n=2 per study group.

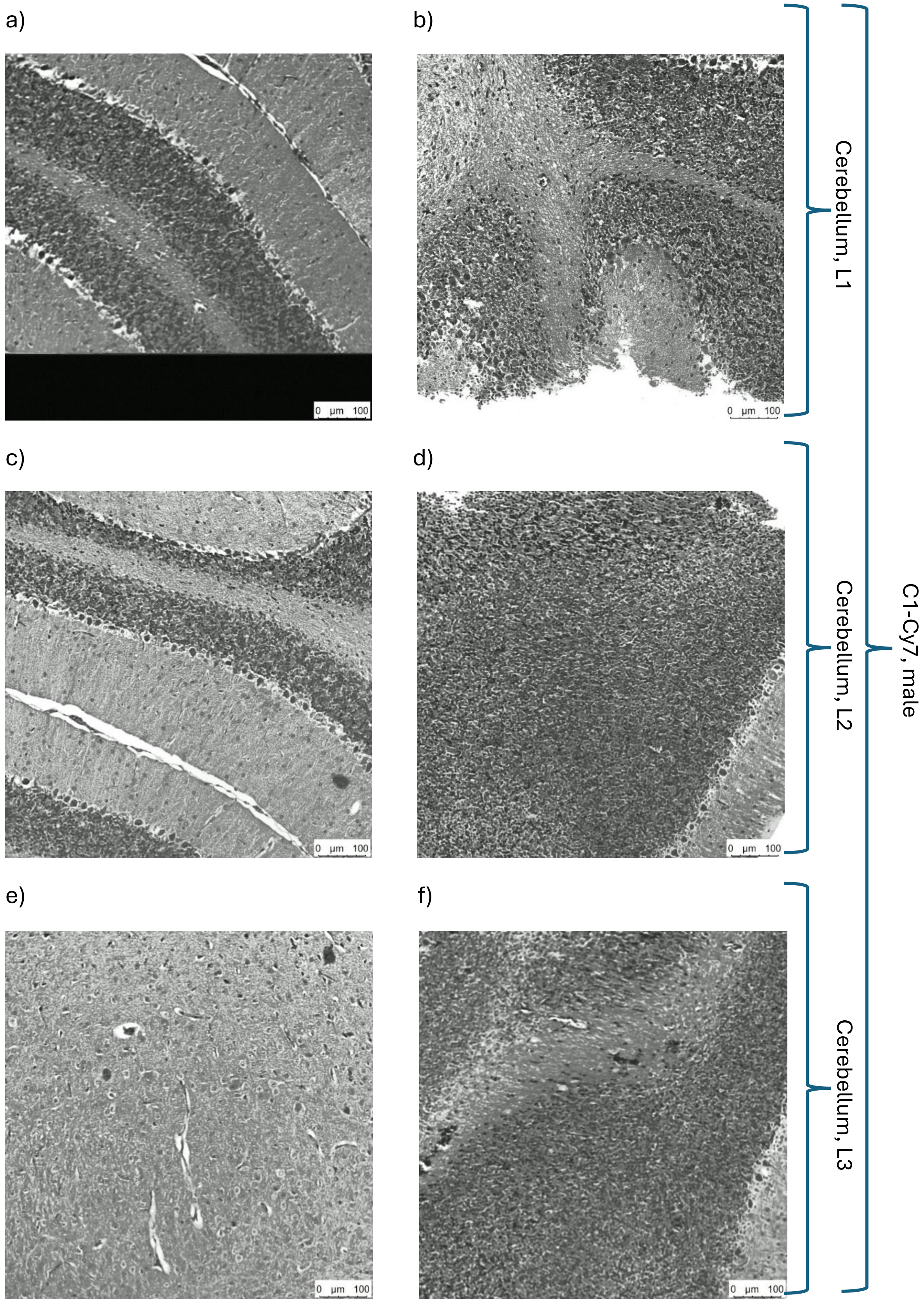

a)
b)
c)
d)
e)
f)
0 µm 100
Cerebellum, L1
Cerebellum, L2
Cerebellum, L3
C1-Cy7, male

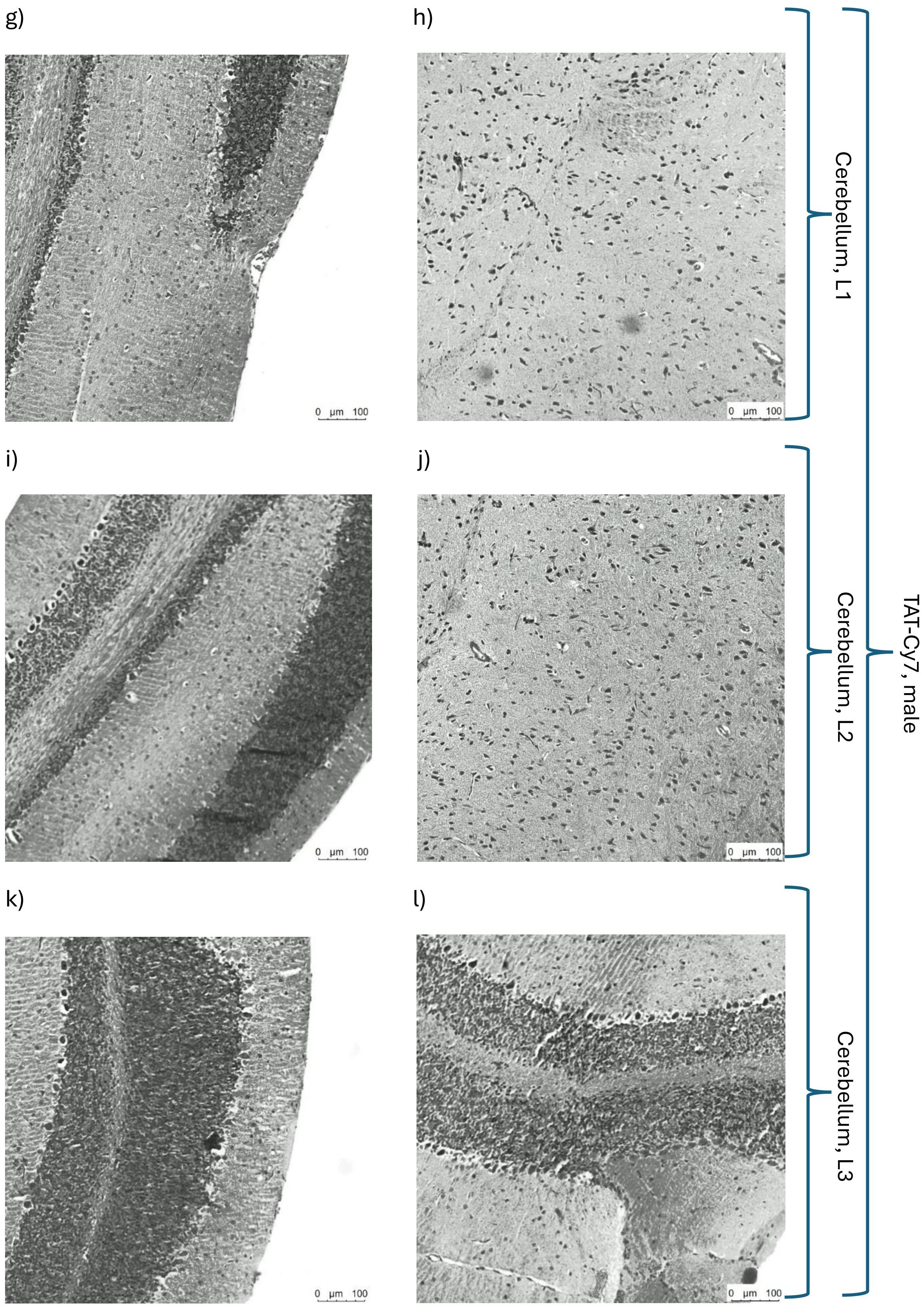
g)
h)
i)
j)
k)
l)
0 µm 100
Cerebellum, L1
Cerebellum, L2
Cerebellum, L3
TAT-Cy7, male

m) Cerebellum, L1

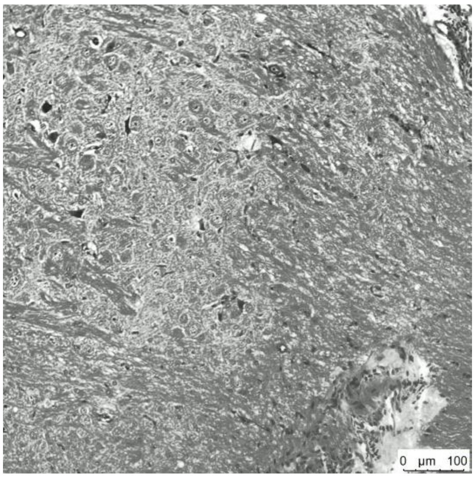

n) Cerebellum, L2

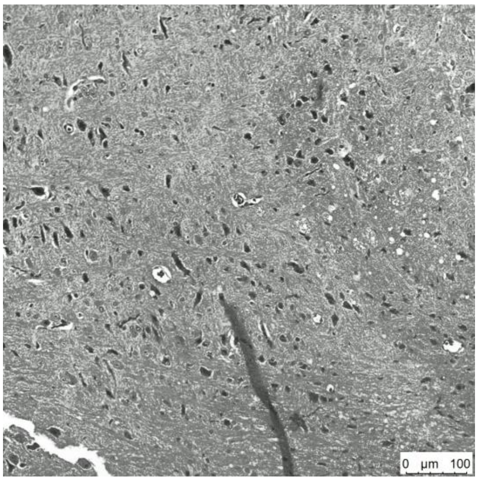

o) Cerebellum, L3

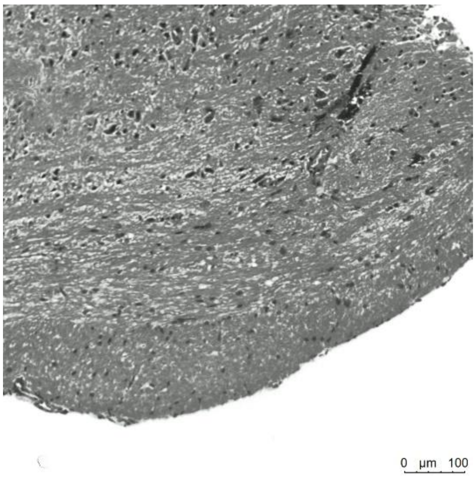

PBS, male

Figure S32. Light micrograph images of H&E stained brain tissues from male mice treated with C1-Cy7, TAT-Cy7 and phosphate buffer saline (PBS) (control); cerebellum; C1-Cy7, L1: a),b); L2, c),d); L3, e),f); TAT-Cy7, L1, g),h); L2,i),j); L3, k), l); PBS, L1, m); L2, n); L3, o). n=2 per study group.

Table S11. Brain pathology scores for mice treatment groups – analysis of gliosis and cell infiltrates. n=2 per study group.

Study group/Pathology	L1	L2	L3	Total score
F/C1-Cy7	0	0	0	0
F/TAT-Cy7	0	0	0	0
M/C1-Cy7	0	0	0	0
M/TAT-Cy7	0	0	1	1

F = female; M = male. L1-L3 are dorsal-ventral orientation levels.

Table S12. Analysis of cell count per cm^2 in H&E strained brain tissue samples of mice. n=3 per study group.

Mice/Treatment group		Number of cells/cm^2		Ratio treated/control		
**Females**						
	L1f	L2f	L3f	L1f	L2f	L3f
C1-Cy7	6	12	10	1	1.5	1
TAT-Cy7	3	6	9	0.5	0.75	0.9
PBS	6	8	10			
	L1m	L2m	L3m	L1m	L2m	L3m
C1-Cy7	10	7	5	1.0	0.9	0.8
TAT-Cy7	3	8	8	0.3	1.0	1.3
PBS	10	8	6			
	L1c	L2c	L3c	L1c	L2c	L3c
C1-Cy7	7	5	4	1.2	0.8	0.7
TAT-Cy7	6	6	6	1.0	1.0	1.0
PBS	6	6	6			
**Males**				Ratio treated/control		
	L1f	L2f	L3f	L1f	L2f	L3f
C1-Cy7	10	10	7	1.0	1.0	1.0
TAT-Cy7	10	10	7	1.0	1.0	1.0
PBS	10	10	7			
	L1m	L2m	L3m	L1m	L2m	L3m
C1-Cy7	6	6	8	1.2	1.2	2.0
TAT-Cy7	5	5	2	1.0	1.0	0.5
PBS	5	5	4			
	L1c	L2c	L3c	L1c	L2c	L3c
C1-Cy7	20	20	10	2.5	2.5	1.0
TAT-Cy7	10	8	6	1.3	1.0	0.6
PBS	8	8	10			

10. Statistical analyses

Data was analyzed in R. Data normality was validated with Shapiro-Wilk test. ANOVA was applied for study group comparison (R).

11. References

[35] A. Herrera, J. Zhou, M.-S. Song, J. J. Rossi, *Methods Mol Biol* **2023**, *2666*, 317–346.

[36] C. L. Scudamore, Ed. , *A Practical Guide to the Histology of the Mouse*, Wiley, **2014**.

[37] K. E. Ibrahim, M. G. Al-Mutary, A. O. Bakhiet, H. A. Khan, *Molecules* **2018**, *23*, 1848.

[38] S. Yamaguchi, R. Sedaka, C. Kapadia, J. Huang, J.-S. Hsu, T. F. Berryhill, L. Wilson, S. Barnes, C. Lovelady, Y. Oduk, R. M. Williams, E. A. Jaimes, D. A. Heller, T. Saigusa, *Sci Rep* **2024**, *14*, 15140.

[39] C. Zhang, Y. Teng, F. Li, W. Ho, X. Bai, X. Xu, X.-Q. Zhang, *ACS Nano* **2023**, *17*, 14852–14870.

[40] S. Zhu, J. Zhang, L. Zhang, W. Ma, N. Man, Y. Liu, W. Zhou, J. Lin, P. Wei, P. Jin, Y. Zhang, Y. Hu, E. Gu, X. Lu, Z. Yang, X. Liu, L. Bai, L. Wen, *Adv Healthc Mater* **2017**, *6*, DOI 10.1002/adhm.201601252.

[41] A. W. Suttie, *Toxicol Pathol* **2006**, *34*, 466–503.

[42] Y. Goltsev, N. Samusik, J. Kennedy-Darling, S. Bhate, M. Hale, G. Vazquez, S. Black, G. P. Nolan, *Cell* **2018**, *174*, 968-981.e15.

[43] T. K. Niethamer, C. T. Stabler, J. P. Leach, J. A. Zepp, M. P. Morley, A. Babu, S. Zhou, E. E. Morrisey, *Elife* **2020**, *9*, DOI 10.7554/eLife.53072.

[44] T.-V. Hoang, C. Nardiello, D. E. Surate Solaligue, J. A. Rodríguez-Castillo, P. Rath, K. Mayer, I. Vadász, S. Herold, K. Ahlbrecht, W. Seeger, R. E. Morty, *J Anat* **2018**, *232*, 472–484.

[45] M. Poxleitner, S. H. L. Hoffmann, G. Berezhnoy, T. M. Ionescu, I. Gonzalez-Menendez, F. C. Maier, D. Seyfried, W. Ehrlichmann, L. Quintanilla-Martinez, A. M. Schmid, G. Reischl, C. Trautwein, A. Maurer, B. J. Pichler, K. Herfert, N. Beziere, *J Neuroinflammation* **2024**, *21*, 129.

[46] H. Ngo-Thanh, T. D. Thuy, K. Suzue, W. Kamitani, H. Yokoo, K. Isoda, C. Shimokawa, H. Hisaeda, T. Imai, *Food Chem Toxicol* **2021**, *151*, 112132.

[47] H. Yamaguchi, J. Shen, *Methods Mol Biol* **2013**, *1004*, 91–113.
